# Clines on the seashore: The genomic architecture underlying rapid divergence in the face of gene flow

**DOI:** 10.1002/evl3.74

**Published:** 2018-08-07

**Authors:** Anja M. Westram, Marina Rafajlović, Pragya Chaube, Rui Faria, Tomas Larsson, Marina Panova, Mark Ravinet, Anders Blomberg, Bernhard Mehlig, Kerstin Johannesson, Roger Butlin

**Affiliations:** ^1^ Department of Animal and Plant Sciences University of Sheffield UK; ^2^ Current address: IST Austria Am Campus 1 3400 Klosterneuburg Austria; ^3^ Department of Marine Sciences University of Gothenburg 40530 Gothenburg Sweden; ^4^ Department of Physics University of Gothenburg 41296 Gothenburg Sweden; ^5^ Department of Marine Sciences ‐ Tjärnö University of Gothenburg 45296 Strömstad Sweden; ^6^ CEES (Centre for Ecological and Evolutionary Synthesis) University of Oslo Oslo 0316 Norway; ^7^ Department of Chemistry and Molecular Biology University of Gothenburg 40530 Gothenburg Sweden

**Keywords:** clines, hybrid zones, inversions, local adaptation, molluscs, speciation

## Abstract

Adaptive divergence and speciation may happen despite opposition by gene flow. Identifying the genomic basis underlying divergence with gene flow is a major task in evolutionary genomics. Most approaches (e.g., outlier scans) focus on genomic regions of high differentiation. However, not all genomic architectures potentially underlying divergence are expected to show extreme differentiation. Here, we develop an approach that combines hybrid zone analysis (i.e., focuses on spatial patterns of allele frequency change) with system‐specific simulations to identify loci inconsistent with neutral evolution. We apply this to a genome‐wide SNP set from an ideally suited study organism, the intertidal snail *Littorina saxatilis*, which shows primary divergence between ecotypes associated with different shore habitats. We detect many SNPs with clinal patterns, most of which are consistent with neutrality. Among non‐neutral SNPs, most are located within three large putative inversions differentiating ecotypes. Many non‐neutral SNPs show relatively low levels of differentiation. We discuss potential reasons for this pattern, including loose linkage to selected variants, polygenic adaptation and a component of balancing selection within populations (which may be expected for inversions). Our work is in line with theory predicting a role for inversions in divergence, and emphasizes that genomic regions contributing to divergence may not always be accessible with methods purely based on allele frequency differences. These conclusions call for approaches that take spatial patterns of allele frequency change into account in other systems.

**Impact Summary**

Adaptive divergence and speciation may often occur under gene flow. A key question in evolutionary biology is: What mechanisms allow divergent selection to succeed despite this opposition by gene flow? Analyzing hybrid zones can help in answering this question. We use data from the marine snail *Littorina saxatilis*, combining genome‐wide sequence data with hybrid zone analysis, a genome assembly, a genetic map and simulations to distinguish loci affected by selection from neutral loci. We identify many loci that are inconsistent with neutral evolution, many of which are located in three large putative genomic rearrangements that we report here for the first time. We also find that many non‐neutral SNPs show relatively low levels of differentiation, and discuss potential reasons, including polygenic adaptation, loose linkage to selected loci, and balancing selection within populations. Our results demonstrate the power of combining modeling with genomic data on individuals from intensive hybrid zone sampling.

Adaptive divergence is a key process generating biodiversity: it causes intraspecies genetic and phenotypic structure and may ultimately lead to speciation (Schluter 2001; Nosil 2012). However, gene flow counteracts divergence (Lenormand 2002), as weakly locally adapted alleles may be “swamped” and recombination may break up locally favorable allele combinations (Felsenstein 1981). Nevertheless, numerous taxa evolve and maintain divergence in the face of gene flow (Pinho and Hey 2010; Butlin et al. 2014; Ravinet et al. 2017, 2018). This requires selection pressures that are strong enough to overcome the homogenizing effects of gene flow. In addition, theory predicts that adaptive divergence might be facilitated by genomic architectures that are well‐suited to resist gene flow (Garant et al. 2006; Smadja and Butlin 2011). Such architectures reduce the potential for recombination to break up locally favorable allele combinations (Smadja and Butlin 2011) and include loci with large phenotypic effects, clusters of divergently selected loci and chromosomal rearrangements containing multiple selected loci (Kirkpatrick and Barton 2006; Faria and Navarro 2010; Yeaman and Whitlock 2011; Rafajlović et al. 2016).

Empirical work detailing the genomic architectures and selection pressures associated with adaptive divergence is still limited to a relatively small number of systems, and may suffer from bias. Most studies so far have applied genome scans, identifying loci with elevated levels of differentiation between populations (e.g., F_ST_). While many important insights have been obtained, standard F_ST_ scans suffer from several caveats. F_ST_ is not always a good indicator of divergent selection, as it is affected by confounding factors including drift, demographic history, and background selection (Noor and Bennett 2009; Cruickshank and Hahn 2014; Ravinet et al. 2017). Moreover, even aside from confounding factors, loci contributing to divergence may not necessarily be expected to show strongly elevated differentiation. For example, if a large number of loci underlies a trait, divergence can be achieved by an increased covariance of allelic effects, while allele frequency differences remain relatively small (Le Corre and Kremer 2012; Yeaman 2015). As another example, divergently selected loci might be affected by balancing selection within populations at the same time. This may be expected especially in genomic regions where chromosomal rearrangements (e.g., inversions) segregate. A possible reason for this is as follows: recombination between inverted and ancestral haplotypes is not possible or strongly reduced. Therefore, the two haplotypes may accumulate different sets of recessive deleterious mutations, and/or different sets of universally adaptive alleles, over time. This may lead to increased fitness in heterozygote individuals, generating balancing selection. This effect is not mutually exclusive with different karyotypes being favored in different habitats, and can therefore lead to divergence without fixation. These examples (polygenic and balancing selection) show that using F_ST_ to detect divergently selected genomic regions may bias against certain genomic architectures. In addition, the nature of the divergent selection pressures often remains obscure when genome scans are used, because linking outlier loci to specific phenotypes or environmental factors is difficult.

Hybrid zone analysis offers a promising approach that may contribute to solving these problems (Harrison and Larson 2016; Ravinet et al. 2017). Its key feature is a difference in sampling scheme: Rather than using distinct spatially separate samples, it involves samples from the continuum between diverging populations across an environmental transition. Allele frequencies at divergently selected loci are expected to change clinally (i.e., gradually), reflecting the antagonism between divergent selection and gene flow (Barton and Hewitt 1985; Barton and Gale 1993). The slope at the cline center is expected to reflect the strength of divergent selection (Slatkin 1973; Barton and Hewitt 1985; Barton and Gale 1993). Consequently, cline analysis should allow for the identification of divergently selected loci that do not show high F_ST_ (but do have steep clines).

In addition, hybrid zones can help to establish phenotype‐genotype‐selection links that are impossible to obtain from outlier scan data alone. The centers of spatial clines for genotypes and phenotypes are expected to co‐locate with the environmental transition driving divergence; associations between divergent traits and causative genomic variants are expected within the hybrid zone, allowing for the identification of genomic regions involved in adaptation (e.g., Lindtke et al. 2013).

Hybrid zone analysis has traditionally been applied to highly divergent populations where clines formed after secondary contact, and has often used relatively small numbers of genetic markers (e.g., Szymura and Barton 1986). In contrast, hybrid zone analysis on a genome‐wide scale is just beginning (Vines et al. 2016; Gompert et al. 2017; Stankowski et al. 2017), and has not been widely applied to systems with extensive gene flow during the course of divergence. A key requirement is to establish the expectation for neutral loci: i.e., to identify the distribution of cline parameters for loci unlinked to selected loci under a realistic demographic model. Only then is it possible to identify non‐neutral loci deviating from this expectation.

We have studied a hybrid zone between two ecotypes of the marine snail *Littorina saxatilis*. In this species, divergent ecotypes have evolved in multiple locations across Europe despite ongoing gene flow (i.e., primary divergence) (Panova et al. 2006; Butlin et al. 2014). In our sampling area in Sweden, the “Crab ecotype” occupies boulder fields inhabited by predatory crabs; the “Wave ecotype” lives on steep cliffs exposed to heavy wave action (Johannesson et al. 2010). The Crab ecotype is much larger, thicker‐shelled, and more wary than the Wave ecotype (Johannesson et al. 2010). It is clear that both crab predation and wave exposure contribute to divergence (Johannesson 1986; Boulding et al. 2017; Le Pennec et al. 2017), but their relative importance remains uncertain. Crab and Wave ecotypes also differ strongly with regard to shell color. There is good evidence for selection on shell colors in this habitat (Johannesson and Butlin 2017), but the exact source and mechanism of selection is unclear.

Assortative mating and habitat choice may contribute to reproductive isolation between ecotypes (Johannesson et al. 2010). Despite these reproductive barriers, hybrid zones have formed at the (typically sharp) environmental transitions between cliffs and boulder fields (Panova et al. 2006; Hollander et al. 2015). Hybrid zones are narrow (tens of meters), as *L. saxatilis* is ovoviviparous (gives birth to juvenile snails rather than laying eggs) and lacks a pelagic larval stage, reducing dispersal (Reid 1996).

We sampled, genotyped and phenotyped snails across a hybrid zone on the Swedish west coast to explore the potential of analyzing a primary hybrid zone with genomic data. Specifically, we aimed to: (1) develop an approach to identify loci under direct or linked divergent selection using cline analysis, and ask whether evidence for divergent selection is necessarily associated with high levels of differentiation; (2) test how loci influenced by divergent selection are distributed across the genome, and whether they form clusters; and finally, (3) test how phenotypes and allele frequencies change in space, to identify selective axes and other factors influencing cline patterns.

## Methods

### SAMPLING, HABITAT, AND PHENOTYPES

**Figure 1:**
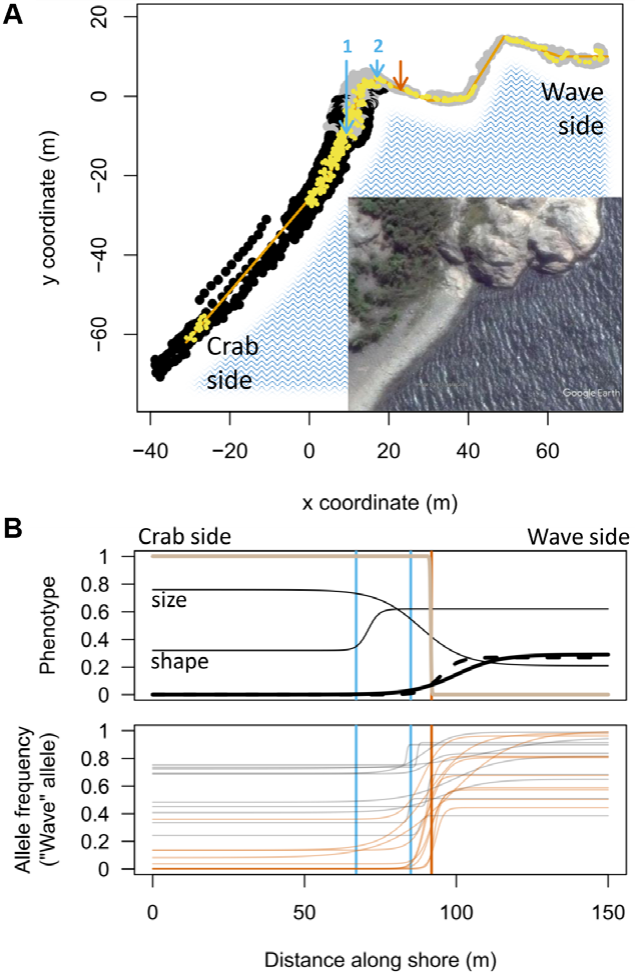
A) Map of the sampled shore area. Habitat points in the boulder field are shown in black, and points on bedrock in grey. Each sampled snail is represented by a yellow point, and the one‐dimensional path through the sampled area is indicated in orange. There are two main habitat transitions: arrow 1, from the boulder field to the rock platform; and arrow 2, from the rock platform to the steep cliff. The orange arrow indicates the average center of all non‐neutral clines (see Fig. 4). Note that the two large sampling gaps in the “Crab” and the “Wave” area represent intentional breaks in the sampling, while the small gap coinciding with the average cline center (orange arrow) represents a gap in the snail distribution. Insert: satellite image from Google Earth (Image © 2017 DigitalGlobe). B) Examples of phenotypic and genetic clines. The x‐axis represents the path through the sampled transect shown in (A). Vertical lines indicate the positions of the arrows in (A). The top panel shows five different phenotypic clines. Thick lines represent frequencies of different colors/patterns (beige, black, and banded); thin lines represent size and shape (scaled to vary between 0 and 1. Note that for analyses, scaling was done so that it ensured an increase from Crab to Wave. In this figure the size cline is reverted to show that the Crab ecotype is larger). The second panel shows examples of genetic clines, with grey curves representing clines consistent with neutrality and orange curves representing non‐neutral clines.

We sampled 600 snails from a transect of ∼150 m at Ängklåvebukten (“ANG”, 58.8697°, 11.1197°) on the Swedish west coast in June 2013 (Fig. 1A). For each snail, we recorded its exact position in three dimensions and photographed its shell before preserving tissue in ethanol. To allow one‐dimensional cline fitting, snail positions were reduced to a path along the shore following the center of the snail distribution. A line consisting of 11 straight segments and following the center of the sampling area was adjusted using a custom R script to minimize the mean squared distance of sample (x,y) coordinates from the line (orange line in Fig. 1A). The nearest position on this line was found for each snail and the cumulative distance from the north end of the transect (“Crab” environment) to this point was used in cline fitting. We also recorded habitat features (boulder vs. cliff substrate) at 1,663 points on the transect.

Shell color and pattern were classified from the photographs; shell size and shape were obtained from 15 landmarks (Ravinet et al. 2016). Quantitative phenotypes (size and shape) were rescaled to range between 0 and 1, for ease of comparison among traits and with SNP clines, such that the most extreme Crab ecotype individual had a score of 0 and the most extreme Wave ecotype individual had a score of 1. Clines were fitted to these phenotypes and to color/pattern morph frequencies using custom R scripts (Supporting Information Appendix, Methods S1).

### REFERENCE GENOME ASSEMBLY AND LINKAGE MAP CONSTRUCTION

We assembled a *L. saxatilis* draft reference genome based on sequencing data from a single Crab ecotype individual (N50 44,284 bp; NG50 [based on genome size 1.35 Gbp] 55,450 bp; maximum scaffold length 608,273 bp; total number of contigs 388,619; Supporting Information Appendix, Methods S2, Tables S1–S3). We also generated a linkage map for *L. saxatilis*, using a single Crab ecotype F1 family sequenced with the same capture sequencing probes used for the hybrid zone analysis (details in Supporting Information Appendix, Methods S3). We obtained 17 linkage groups (LGs), as expected from the *L. saxatilis* karyotype (Janson 1983a; Rolán‐Alvarez et al. 1996), between 45.5 and 88.8 cM (centimorgan) long. Total map length was 1011.9 cM and resolution was ∼0.2 cM. Therefore, most map positions are associated with multiple scaffolds or contigs (and SNPs).

### DNA EXTRACTION, CAPTURE SEQUENCING, AND BIOINFORMATICS

DNA was extracted from a piece of foot tissue using a CTAB protocol (Panova et al. 2016) for 373 sexually mature individuals from the transect sample. Targeted capture sequencing (Illumina) was applied with 40,000 probes, randomly distributed across the genome (Supporting Information Appendix, Methods S4). Reads were filtered and mapped to the *L. saxatilis* genome assembly using a custom pipeline (Supporting Information Appendix, Methods S4). Either SNP calls or allelic read depths were used in subsequent analyses, retaining only SNPs within 1,000 bp of a SNP included in our linkage map.

### CLINE ANALYSIS AND SIMULATIONS

After further filtering for departure from Hardy–Weinberg expectations, sex differences, and allele‐frequency patterns, we fitted clines for each SNP using read‐depth data rather than relying on genotype calls. We fitted several cline models (simple sigmoid clines, left‐tailed clines, right‐tailed clines, two‐tailed clines; equations in Derryberry et al. (2014)) using maximum likelihood estimation (bbmle package in R, function mle2, Bolker and R Development Core Team 2012). Akaike's Information Criterion (AIC) was used to select the best model, with ∆AIC > 4. For details, see Supporting Information Appendix, Methods S5.

To distinguish neutral clines from clines indicating the direct effect of divergent selection, or its indirect effect on linked loci, we used simulations tailored closely to our system (for details see Supplementary Document S1). Very briefly, we simulated individuals in a system of primary divergence for 4,000 generations. We constructed individual‐based simulations of a chain of 152 demes, each deme assumed to be 1 m wide, with a change of environment after deme 85 (so that the position of the simulated environmental transition corresponded to the observed one). Individuals were diploid and carried sets of loci under divergent selection (*n* = 200), as well as unlinked neutral loci. Wherever possible, parameters were chosen based on empirical estimates. In particular, the total selection coefficient was *s* = 0.7 (Janson 1983b) and dispersal distance was estimated from the elevation in LD at the cline center (Supplementary Information Appendix, Methods S6). Simulation output was analyzed with the same scripts used for observational data. Simulations are described in full in Supplementary Document S1.

### ASSOCIATION, HERITABILITY, AND LINKAGE DISEQUILIBRIUM ANALYSES

For 106,599 SNPs passing filters, imputation of missing genotypes was performed using LinkImpute (Money et al. 2015). An association analysis was performed for all measured traits using the egscore() function from the GenABEL R package (Aulchenko et al. 2007), which implements the EIGENSTRAT method (Price et al. 2006). For continuous traits, the software HEIDI (Kostem and Eskin 2013) was used to estimate the overall heritability and to partition heritability among chromosomes. For LGs 6, 14, and 17, we also partitioned the contributions of large blocks enriched in non‐neutral SNPs (“nnBlocks”; see below) and the rest of the LG.

The data set used for association mapping was also used to calculate LD between SNPs within nnBlocks and outside nnBlocks on the same linkage groups (LG6, LG14, and LG17) using the *genetics* package in R (https://CRAN.R-project.org/package=genetics).

For further details see Supporting Information Appendix, Methods S7.

## Results

### SHORE STRUCTURE AND PHENOTYPIC PATTERNS

We obtained 600 snails from a 152 m transect along the shore (Fig. 1). The transect covers two habitat transitions: one from the crab‐inhabited boulder field to a rock platform, and one from the rock platform to the near‐vertical, wave‐exposed cliff (Fig. 1A, “1” and “2”, respectively). As the rock platform is subject to increased wave exposure, but accessible to crabs, “Crab” and “Wave” selection pressures change somewhat independently. All measured phenotypic traits (shell shape, centroid size, and four colors [beige, dark beige, black, banded]) varied clinally along the transect (Fig. 1B; Supporting Information Appendix, Figs. S1 and S2, Tables S4 and S5).

### IDENTIFICATION OF NON‐NEUTRAL SNPS AND EXTENT OF DIFFERENTIATION

**Figure 2:**
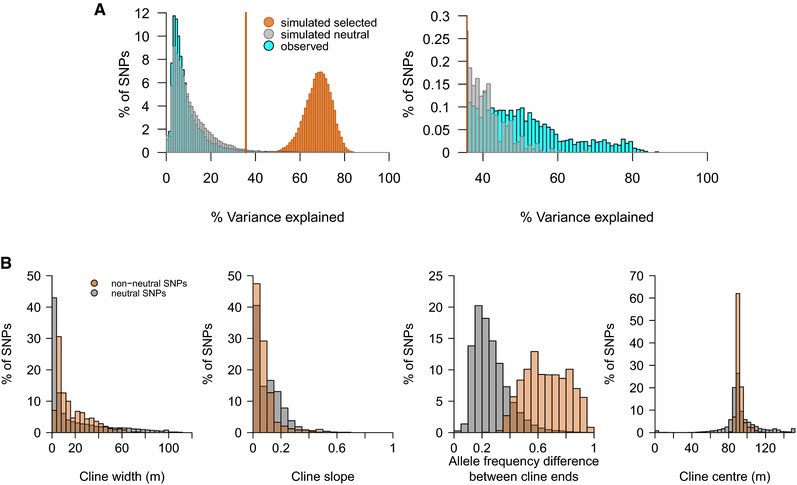
Variation in cline parameters. A) Comparison of variance explained (var.ex) between simulated selected SNPs (light orange), simulated neutral SNPs (grey), and observed SNPs (light blue). The orange line indicates the 99% quantile of the simulated neutral distribution. The right plot is restricted to var.ex values above this threshold, and shows observed and simulated neutral distributions only, to highlight the difference in the distribution tails. B) Observed neutral (grey) and non‐neutral SNPs (i.e., var.ex above threshold in A; orange). Note that there were many more neutral than non‐neutral SNPs; here, values in each class are expressed as percentages of all SNPs in that class.

After mapping the capture sequencing reads of the 373 genotyped individuals from the hybrid zone against the reference genome, we obtained 146,671 SNPs on 11,775 contigs passing filters. Spatial patterns at 75,562 SNPs (51.5%) were better explained by a model of clinal change than by a model with constant allele frequency, based on an AIC difference of at least 4 (hereafter “clinal SNPs;” Supporting Information Appendix, Table S6). For these, we estimated cline width, slope (calculated as the product of the allele frequency difference between cline ends and the inverse of the cline width), center, and allele frequencies at the “Crab” and “Wave” ends of the cline (Supplementary Information Appendix, Methods S5). The variance explained by the cline models (var.ex) was generally low (under 20% for the vast majority of SNPs, Fig. 2A, light blue). It is likely that many of these clinal SNPs are neutral, with clines generated by isolation‐by‐distance combined with a genome‐wide reduction of gene flow across the habitat transition(s). To distinguish neutral clines from those indicating selection, we compared the observed clines with neutral and selected simulated clines. As expected, all simulated selected SNPs showed steep clines, and the cline model explained a large proportion of the variance in the read count data (high var.ex; Fig. 2A). Of the simulated neutral SNPs, 66.8% showed clines; however, cline slopes and var.ex were clearly lower than for simulated selected SNPs (Fig. 2A). In contrast to the simulated selected SNPs, most SNPs in our observed dataset are probably not under direct selection, and the observed data are noisier than the simulated data. For these reasons, we did not expect observed SNPs affected by divergent selection to show var.ex values as high as those found for simulated selected SNPs. However, the comparison between simulated neutral and selected SNPs does indicate that the var.ex can be used as a criterion to identify SNPs that are inconsistent with neutrality and may be affected by divergent selection. We therefore identified observed non‐neutral SNPs as those with var.ex above a threshold defined by the 99^th^ percentile of simulated neutral loci (threshold = 35.69; Fig. 2A). Some of these 1,891 putatively non‐neutral SNPs (1.4% of all SNPs included in the cline fitting) are likely to be affected by divergent selection, while some are likely to be false positives. Overall, 226 SNPs had higher var.ex than observed at all in the neutral simulated data (Fig. 2A).

Non‐neutral SNPs showed wider clines, increased allele frequency differences between cline ends, and a closer association of cline centers with the habitat transitions compared to SNPs consistent with neutrality (Fig. 2B). A greater average cline width of non‐neutral SNPs was explained by the fact that the full set of clinal SNPs (Fig. 2B) contained many SNPs with width estimates at the lower boundary allowed during model fitting (1 m; see Supporting Information Appendix, Methods S5, Table S7). These narrow clines may often be spurious: they were associated with low var.ex estimates and were therefore not among the clines identified as non‐neutral. All non‐neutral SNPs showed greater than average allele frequency differences (closely related to F_ST_) between cline ends, but differences were often moderate (Fig. 2B) (in contrast to observations for simulated selected SNPs).

### CLUSTERING IN THE GENOME

**Figure 3:**
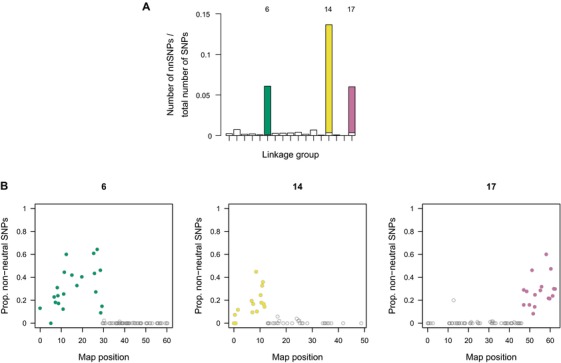
(A) Proportion of SNPs that were non‐neutral in each of the 17 LGs (sorted from 1 to 17 on the x‐axis). SNPs within three large regions of high linkage disequilibrium (“nnBlocks”) are shown in color. B) Variation in the proportion of non‐neutral SNPs among map positions in the three LGs with large numbers of non‐neutral SNPs. nnBlocks are again indicated in colour. All 17 LGs are shown in Supporting Information Appendix, Fig. S4.

Assigning all SNPs variable in the hybrid zone to the closest genetic map position (if within 1,000 bp of a genetic map position), we tested for clustering of non‐neutral SNPs at different genomic scales (Supporting Information Appendix, Methods S8) by applying permutation tests. We found striking clustering at the level of linkage groups: three LGs (6, 14, and 17) contained about three quarters of all non‐neutral SNPs (Fig. 3A). There was also significant clustering by map position within these linkage groups, as well as in LG3, and also by 10 cM intervals in LGs 2, 4, 6, 9, 14, and 17. Significant clustering of non‐neutral SNPs was also observed within contigs, but only below 100 bp (Supporting Information Appendix, Fig. S3).

Notably, LGs 6, 14, and 17 each contained a single region with elevated proportions of non‐neutral SNPs, measuring between 12.5 and 29.5 cM in length (Fig. 3B). Genotypes for non‐neutral SNPs within these regions were correlated (Supporting Information Appendix, Fig. S5). The mean linkage disequilibrium (LD) was greater between SNPs within contigs, and declined more slowly with recombination distance within these regions compared to the remaining parts of LGs 6, 14, and 17 (Supporting Information Appendix, Table S8). In each case, the effect was stronger in one ecotype than the other, probably reflecting levels of polymorphism for an underlying chromosomal rearrangement. In the following, we refer to these blocks of high LD and high concentration of non‐neutral SNPs as nnBlocks (non‐neutral blocks).

**Figure 4:**
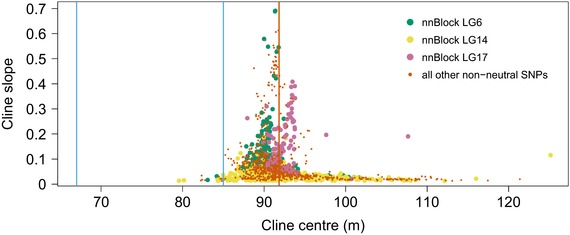
Distribution of cline slopes versus cline centers of non‐neutral SNPs. Non‐neutral SNPs in nnBlocks are shown in colors analogous to previous figures. All other non‐neutral SNPs are shown in orange. The vertical orange line indicates the average of all non‐neutral cline centers. Blue lines indicate habitat transitions as in Fig. 1.

Each of the three nnBlocks had a characteristic pattern of cline slope and differentiation. Whereas LG14 was characterized by high F_ST_ values and relatively shallow slopes, LG6 showed both high F_ST_ and steep slopes and LG17 showed only moderate F_ST_, but many SNPs with very steep slopes (Fig. 4; Supporting Information Appendix, Fig. S6B).

### GENOTYPE–PHENOTYPE‐ENVIRONMENT ASSOCIATIONS

None of the non‐neutral SNPs had cline centers near the habitat transition from boulder field to rock platform (67.0 m; arrow 1 in Fig. 1). Instead, centers were concentrated close to the transition from rock platform to steep cliff (i.e., the transition to a crab‐free area at 85.0 m, arrow 2 in Fig. 1) (Fig. 4). However, the correspondence between non‐neutral cline centers and habitat transition was not perfect, as the average cline center was displaced to 91.8 m (Fig. 4). The average cline center corresponded to a gap in our sampling (orange arrow in Fig. 1A), which reflects a gap in snail distribution, potentially due to an unusually smooth cliff surface without cracks. This shift is unlikely to be an artifact of fitting clines in an area with uneven sampling density because no shift was observed in the simulated data, which mimicked the observed sampling distribution. SNPs within nnBlocks on LG6 and LG17 clustered together, but at slightly different average positions, while cline centers of the LG14 nnBlock were more widely spread (Fig. 4).

No significant single‐locus associations were found for the studied quantitative traits, using the GenABEL package (Aulchenko et al. 2007), despite high heritabilities (size: 0.25 [0.19–0.30], shape: 0.61 [0.38–0.84]; mean [95% confidence interval]). Among the qualitative traits, significant associations were seen for the colors beige and black, and for the banded pattern (Supporting Information Appendix, Fig. S7, Table S9). Only a single color‐associated SNP passed filters for the cline analysis; this one did not show a significant cline (Supporting Information Appendix, Table S9).

Partitioning of the contribution of each chromosome to the overall heritability, using HEIDI (Kostem and Eskin 2013), suggested a concentration of effects on a subset of linkage groups, including those with nnBlocks. For size, six linkage groups had non‐zero contributions, including large effects of LG6 and LG12. For shape, effects were more widespread but LGs 6, 9, 14, and 17 made the largest contributions. When the contributions of LGs 6, 14, and 17 were further partitioned, all or most (>70%) of the effect was attributable to the nnBlocks (Supporting Information Appendix, Fig. S8, Table S10).

### RESULTS WITH ALTERNATIVE SIMULATION PARAMETERS

The cline patterns observed in the simulations, and consequently the var.ex threshold used for identification of non‐neutral SNPs in the observed data, depend on the input parameter values used for the simulations. We performed additional simulations to test sensitivity to input parameter combinations, and to test a model of secondary contact (detailed methods and results in Supplementary Document S1). Our main conclusions are robust to changes in the var.ex threshold under realistic parameter combinations (see figures comparable to Figs. 2, 3, 4, but using the lowest and highest var.ex thresholds obtained across all simulations, in Supporting Information Appendix, Figs. S9–S12; Table S11 for proportions of neutral and non‐neutral SNPs).

## Discussion

Hybrid zone analysis has a long history in the study of divergence and speciation (Barton and Hewitt 1985; Szymura and Barton 1986; Harrison 1993), and has more recently been recommended as a promising approach in combination with high‐throughput genomic data (Abbott et al. 2013; Gompert et al. 2017; Ravinet et al. 2017). This method may not only improve detection of genomic regions affected by divergent selection, but may also facilitate the identification of genomic regions under more complex patterns of selection and the understanding of genotype‐phenotype‐selection links. However, exploiting these opportunities requires extensive sampling and genotyping, plus improved methods of data analysis (Gompert et al. 2012; Lindtke et al. 2012; Parchman et al. 2013; Vines et al. 2016; Stankowski et al. 2017). The simulation approach developed here advances the use of hybrid zones to detect and interpret genomic regions underlying divergence.

### IDENTIFICATION OF NON‐NEUTRAL SNPs AND EXTENT OF DIFFERENTIATION

Clinal patterns proved to be pervasive across the genome. This is not surprising given restricted gene flow between ecotypes and is likely to be the case also in other empirical systems (e.g., Stankowski et al. 2017). Our simulations, informed by our prior knowledge about the demographic history of *L. saxatilis*, showed that significant clines often occur at neutral loci that are not linked to any selected loci. Observing clinal variation is clearly not sufficient evidence to infer divergent selection. However, the simulations provided a means to discriminate loci influenced by direct or linked selection from this background of expected clines for neutral SNPs. This strategy used more information than a typical F_ST_ outlier scan (by including spatial coordinates as well as by using a larger number of samples), and detected a large number of non‐neutral SNPs across the *L. saxatilis* genome.

Non‐neutral SNPs generally showed smaller var.ex and smaller F_ST_ estimates than the simulated selected SNPs. This was expected given that all simulated selected SNPs were under direct selection, while the observed dataset may contain SNPs influenced by various types of selection pressures and strengths of direct or indirect selection. Nevertheless, it is notable that fixed differences between ecotypes were extremely rare in the observed data, and levels of differentiation were generally low. There are several possible explanations for this result. First, it could be explained simply by the presence of SNPs that are linked to selected variants, but not under direct selection. Such SNPs may appear as non‐neutral, while not showing high differentiation because recombination weakens their association with the causal variant. As we used a reduced‐representation dataset, SNPs under direct selection are likely rare in our data, while linked divergent selection may explain patterns at many SNPs. However, it is unlikely that linkage can fully explain the observed patterns, as many non‐neutral SNPs did show steep clines. With increasing recombination distance from a selected locus, not only F_ST_ but also cline slope should decrease.

Selection on polygenic traits may also contribute to this pattern of low differentiation. It has been shown that with polygenic architectures underlying divergent traits, differentiation may not be pronounced (Le Corre and Kremer 2012; Yeaman 2015); in *L. saxatilis*, many traits contributing to divergence (e.g., shell size and thickness) are likely to be highly polygenic.

An additional possible explanation relevant for a subset of our dataset is a combination of divergent and balancing selection. If different optima at the two cline ends are combined with balancing selection (e.g., heterozygote advantage) for the same locus, fixation will be prevented at least at one cline end. This scenario could generate steep clines despite relatively low F_ST_. As a simple example, imagine a biallelic locus, with one allele favored in the Crab habitat and the other allele favored in the Wave habitat. If the allele favored in the Wave habitat is lethal in homozygous form, but heterozygotes are strongly favored in this habitat, then a polymorphism will be maintained in the Wave habitat, preventing fixation.

While prevalent balancing selection across the genome might seem unlikely, many non‐neutral SNPs in our system appear to reside in genomic rearrangements, probably inversions (see below) (Fig. 3). As noted above, some theoretical models predict balancing selection on inversions, and this has been supported by observational evidence (Wellenreuther et al. 2017). Balancing selection on inversions is therefore a possible explanation for the low differentiation of some of the non‐neutral SNPs we observed, specifically for the SNPs in the LG17 nnBlock, which showed particularly low differentiation (Fig. S6B).

As an example of a differentiation‐based outlier scan, which we expected to bias against regions affected by divergent selection but with limited differentiation, we ran a BayeScan (Foll and Gaggiotti 2008) analysis and compared it with our cline analysis (Supplementary Information Appendix, Methods S9, Fig. S13). The results show a strong overlap between analyses (∼70% of outlier SNPs were identical under our settings), owing to the fact that both analyses identify loci of high differentiation. However, the BayeScan outlier analysis was systematically biased against the low‐differentiation SNPs detected by the cline analysis. For example, the nnBlock on LG17, which showed strong patterns of LD and strong evidence of selection, could not be detected with the BayeScan analysis under standard settings, and many SNPs in this region remained undetectable even under lenient settings (Supplementary Information Appendix, Fig. S13). This is not an issue of sample size (which was reduced in the outlier scan, as only individuals from the cline ends could be used): Even if the number of individuals used for the F_ST_ outlier scan was increased, these SNPs would remain undetectable due to their low levels of differentiation. These results highlight that some architectures underlying adaptive divergence may be undetectable with differentiation‐based outlier scans, but can be identified based on patterns of clinal allele frequency change.

While custom simulation combined with hybrid zone data is a powerful approach to detecting genomic regions under selection, it shares some limitations with other approaches (Ravinet et al. 2017). Cline patterns, like patterns of F_ST_, may be affected by the genomic distribution of recombination rates and gene density (Martin et al. 2016; Burri 2017). In addition, also for cline analysis a threshold must be defined above which SNPs are considered “non‐neutral”; this threshold reflects a trade‐off between false‐positive and false‐negative rates. Further data, e.g. from experiments, will therefore be necessary to test candidate loci. Nevertheless, our results demonstrate how cline analysis can be used to improve the identification of loci affected by divergent selection and to understand the form of selection. It provides a promising approach that can be applied to genome‐wide data in many other systems.

### CLUSTERING IN THE GENOME

While there is empirical support for the prediction that divergence with gene flow leads to genomic clustering of selected loci (Samuk et al. 2017) or their concentration in chromosomal rearrangements (Twyford and Friedman 2015; Barth et al. 2017), there is also evidence for many loci scattered across the genome in some taxa (Jones et al. 2012; Renaut et al. 2013; Henning et al. 2017). For *L. saxatilis*, we have generated the first linkage map and combined it with the non‐neutral SNPs detected by cline analysis. We found non‐neutral SNPs to be widespread across the genome, as expected because multiple traits (many of them likely to be polygenic) contribute to divergence and change gradually across the hybrid zone (Johannesson et al. 2010; Hollander et al. 2015; Le Pennec et al. 2017). However, we did also find evidence for clustering of non‐neutral SNPs, both at the level of linkage groups and map positions. Specifically, three quarters of non‐neutral SNPs were located in only three large genomic regions (nnBlocks; 12.5 to 29.5 cM long) showing high levels of LD. These blocks cannot be explained by strong selection on many individual loci along the chromosome alone, as many individuals were heterozygous across whole blocks (Supporting Information Appendix, Fig. S5). Neither can they be explained by generally low recombination rates in these regions, as numerous recombination events occurred in the cross for the linkage map (Crab x Crab cross). The most likely explanations for the observed patterns are chromosomal rearrangements, probably inversions, with ecotypes differing in karyotype frequencies. Such rearrangements suppress recombination in heterokaryotypes, explaining why divergently selected SNPs and linked SNPs may be maintained in long blocks, but allow for normal recombination in homokaryotypes, consistent with recombination in the linkage map cross. The clustering of non‐neutral SNPs in rearrangements is in line with both theoretical (Navarro and Barton 2003; Kirkpatrick and Barton 2006; Faria and Navarro 2010) and empirical (Jones et al. 2012; Twyford and Friedman 2015) work demonstrating the role inversions may play in adaptive divergence and speciation by preventing recombination between alleles adapted to the same environment, or between these alleles and alleles contributing to other components of reproductive isolation.

However, as SNPs within a rearranged genomic region are not independent, our current dataset cannot provide information about the number of selected loci located within each rearranged region. In principle, just a single locus under divergent selection might generate the observed differentiation along a large genomic region. However, we find that multiple divergently selected traits are associated with the rearrangements, indicating that multiple loci are involved (see below).

Further work is needed to study the role and number of individual loci within the putative rearrangements, and to experimentally test the hypothesis of balancing selection, e.g. by testing for heterozygote advantage in lab populations. The potential interaction between divergent and balancing selection may also add a new angle to research on the role of inversions in speciation: In contrast to expectations from most existing models, inversions might impede the completion of speciation if balancing selection prevents fixation. This hypothesis requires additional work, both in terms of theoretical modeling and empirical tests in other taxa.

### GENOTYPE‐PHENOTYPE‐ENVIRONMENT ASSOCIATIONS

In hybrid zones, association analysis can be used to test which chromosomal regions contain loci underlying adaptive phenotypes. Of three mappable color traits (which also showed clinal changes across the hybrid zone), one (banded) showed strong associations near the boundary of the LG6 nnBlock, and two of the banding‐associated SNPs were located on the same contigs as multiple nnSNPs. We found no significant single‐locus association for shell size or shape, which may be influenced by multiple loci of small effect. However, when partitioning the variation among linkage groups and between regions within and outside nnBlocks, we found that the nnBlocks on LG6, 14, and 17 disproportionately contribute to variation in these quantitative traits. These associations are another piece of evidence indicating the importance of these putative genomic rearrangements for divergent adaptation.

One great advantage of hybrid zone analysis is that it can be used to make inferences about the patterns of selection in space. We found that the cline centers of most non‐neutral SNPs were located close to the transition from crab‐free to crab‐infested habitat. This suggests that crabs represent a strong selection pressure driving divergence between ecotypes, as indicated by previous experimental work (Johannesson 1986). In contrast, no non‐neutral cline centers coincided with the transition to wave‐exposed habitat. This is surprising given previous evidence for wave exposure as a selection pressure in this system, and given that the cline center for the shape phenotype (which is likely important for wave resistance (Le Pennec et al. 2017)) roughly co‐locates with this transition (Fig. 1). It is possible that shape variation is underlain by a relatively small number of loci, none of which was captured with our sequencing approach.

We observed that, even though most non‐neutral clines centered near the transition to the steep cliff, they were displaced into the Wave habitat. Dominance or epistasis may displace individual SNP clines; however, the concordant displacement of numerous SNP clines across the genome requires another explanation, as neither dominance nor epistasis is expected to affect all SNPs in similar ways. Instead, cline centers map to an area of low snail density (Fig. 1A). Since this area is close to a habitat transition, the observed patterns are consistent with locally asymmetric dispersal trapping clines in the density trough (Barton and Hewitt 1985). Therefore, the observed spatial patterns do not only give indications about the axes of divergent selection, but also reveal other possible forces affecting allele frequency patterns. None of this information would have been available with standard genome scan analyses, which reduce complex patterns of divergence down to a binary comparison (Stuart et al. 2017).

Overall, our analyses show that the cline‐based approach represents a significant improvement over genome‐scan methods because more information is available to distinguish signatures of selection, including forms of selection that are not simply divergent between habitats, from neutral variation. They reveal a clustered genetic architecture, dominated by large blocks of strong LD, and show how these genomic regions are associated with adaptive phenotypes and environmental transitions. Similar approaches can be applied productively in many other systems.

Associate Editor: S. Wright

## Supporting information


**TABLE S1.1** Summary statistics of the maximum‐likelihood results for simulated allele‐frequency data at neutrally evolving loci, with the number of individuals in each patch set to *N* =100.

**TABLE S1.2** Summary statistics of the maximum‐likelihood results for simulated allele‐frequency data at loci under selection, with the number of individuals in each patch set to *N* =100.

**TABLE S1.3** Percentiles of the variance explained by the maximum‐likelihood clinal fits for simulated neutral loci (null hypothesis), with the number of individuals in each patch set to *N* =100.

**TABLE S1.4** Same as in Tab. S1.1 but for two additional values of the dispersal distance *σ*: *σ* = 1.09 and *σ* = 1.70.

**TABLE S1.5** Same as in Tab. S1.4 but for loci under selection.

**TABLE S1.6** Same as in Tab. S1.3 but for two additional values of the dispersal distance *σ*: *σ* = 1.09 and *σ* = 1.70.

**TABLE S1.7** Same as in Tab. S1.1 but for two additional values of the local population size *N*: *N* = 50 and *N* = 200.

**TABLE S1.8** Same as in Tab. S1.7 but for loci under selection.

**TABLE S1.9** Same as in Tab. S1.3 but for two additional values of the local population size *N*: *N* = 50 and *N* = 200.

**Table S1**: Summary of sequencing libraries used for *Littorina saxatilis* genome assembly.

**Table S2**: Summary of scaffolded genome assembly.

**Table S3**: Summary of BUSCO analyses of scaffolded assembly.

**Table S4**: Parameter estimates for size and shape clines.

**Table S5**: Parameter estimates for colour clines.

**Table S6**: Different categories of SNPs based on cline analysis and simulations testing for neutrality.

**Table S7**: Initial values, lower bounds and upper bounds for maximum likelihood estimation of SNP clines.

**Table S8**: Linkage disequilibrium (absolute correlation coefficient) between SNPs on linkage groups with regions showing high concentrations of non‐neutral SNPs.

**Table S9**: SNPs significantly associated with colour traits according to GenABEL analysis.

**Table S10**: Contributions of linkage groups to the heritability of shell size and shape, estimated in HEIDI.

**Table S11**: Numbers and proportions of clinal SNPs that were considered neutral and non‐neutral.

**Fig. S1**: Fitted clines for transformed centroid size (adjusted to the mean shore height) for females (black) and males (green).
**Fig. S2**: Fitted cline for transformed shape (adjusted to a scaled size of 0.5) for females (black) and males (green).
**Fig. S3**: Number of SNP pairs in which both SNPs are non‐neutral, divided by the number of SNP pairs containing at least one non‐neutral SNP.
**Fig. S4**: Variation in the proportion of non‐neutral SNPs among map positions.
**Fig. S5**: Genotypes at all non‐neutral SNPs placed on the genetic map.
**Fig. S6**: Relationship between F_ST_ and cline slope.
**Fig. S7**: Manhattan plots for (A) banded pattern on the shell, (B) beige colour, and (C) black colour of the shell.
**Fig. S8**: Normalised contribution of each linkage group to shell size (A) and shape (B) variation using HEIDI.
**Fig. S9**: Histograms of cline parameters in neutral and non‐neutral SNPs, based on the 19.26 var.ex threshold (A) and the 47.48 var.ex threshold (B).
**Fig. S10**: Proportion of SNPs that were non‐neutral in each of the 17 LGs (LGs in order along the x‐axis).
**Fig. S11**: Variation in the proportion of non‐neutral SNPs among map positions based on the 19.26 var.ex threshold (A) and the 47.48 var.ex threshold (B).
**Fig. S12**: Distribution of cline slopes and centres of non‐neutral SNPs along the shore, based on the 19.26 var.ex threshold (A) and the 47.48 var.ex threshold (B).
**Fig. S13**: Comparison between cline analysis and BayeScan outlier analysis.
**Fig. S14**: Filtering of variant datasets after SNP calling.

**FIG. S1.1** Maximum‐likelihood estimates of cline centres for simulated allele‐frequency data under the primary divergence model with σ = 1.46.
**FIG. S1.2** Same as in Fig. S1.1, but for the secondary contact model.
**FIG. S1.3** Maximum‐likelihood estimates of the difference of allele frequencies at the two habitat ends for simulated allele‐frequency data under the primary divergence model with σ = 1.46.
**FIG. S1.4** Same as in Fig. S1.3, but for the secondary contact model.
**FIG. S1.5** Maximum‐likelihood estimates of cline width for simulated allele‐frequency data under the primary divergence model with σ = 1.46.
**FIG. S1.6** Same as in Fig. S1.5, but for the secondary contact model.
**FIG. S1.7** Variance explained by the maximum‐likelihood clinal fit for simulated allele‐frequency data under the primary divergence model with σ = 1.46.
**FIG. S1.8** Same as in Fig. S1.7, but for the secondary contact model.
**FIG. S1.9** Maximum‐likelihood estimates of cline slopes (a, c), and effective selection coefficients (per locus) inferred from the estimated slopes (b, d) for loci under selection. Shown are only results for loci designated as clinal under the primary divergence model with σ = 1.46.
**FIG. S1.10** Same as in Fig. S1.9, but for the secondary contact model.

## References

[evl374-bib-0001] Abbott, R. , D. Albach , S. Ansell , J. W. Arntzen , S. J. E. Baird , N. Bierne , et al. 2013 Hybridization and speciation. J. Evol. Biol. 26:229–246. 23323997 10.1111/j.1420-9101.2012.02599.x

[evl374-bib-0002] Aulchenko, Y. S. , S. Ripke , A. Isaacs , V. Duijn , and C. M 2007 GenABEL: an R library for genome‐wide association analysis. Bioinformatics 23:1294–1296. 17384015 10.1093/bioinformatics/btm108

[evl374-bib-0003] Barth, J. M. I. , P. R. Berg , P. R. Jonsson , S. Bonanomi , H. Corell , J. Hemmer‐Hansen , et al. 2017 Genome architecture enables local adaptation of Atlantic cod despite high connectivity. Mol. Ecol. 26:4452–4466. 28626905 10.1111/mec.14207

[evl374-bib-0004] Barton, N. H. , and K. S. Gale . 1993 Genetic analysis of hybrid zones. In: R. G. Harrison, ed. Hybrid zones and the evolutionary process. Oxford Univ. Press, New York.

[evl374-bib-0005] Barton, N. H. , and G. M. Hewitt . 1985 Analysis of hybrid zones. Annu. Rev. Ecol. Syst. 16:113–148.

[evl374-bib-0006] Bolker, B. , and R Development Core Team . 2012 bbmle: Tools for general maximum likelihood estimation. R Package Version. R Foundation for Statistical Computing, Vienna, Austria.

[evl374-bib-0007] Boulding, E. G. , M. J. Rivas , N. González‐Lavín , E. Rolán‐Alvarez , and J. Galindo . 2017 Size selection by a gape‐limited predator of a marine snail: Insights into magic traits for speciation. Ecol. Evol. 7:674–688. 28116062 10.1002/ece3.2659 PMC5243190

[evl374-bib-0008] Burri, R. 2017 Linked selection, demography and the evolution of correlated genomic landscapes in birds and beyond. Mol. Ecol. 26:3853–3856. 28749613 10.1111/mec.14167

[evl374-bib-0009] Butlin, R. K. , M. Saura , G. Charrier , B. Jackson , C. André , A. Caballero , et al. 2014 Parallel evolution of local adaptation and reproductive isolation in the face of gene flow. Evolution 68:935–949. 24299519 10.1111/evo.12329 PMC4261988

[evl374-bib-0010] Cruickshank, T. E. , and M. W. Hahn 2014 Reanalysis suggests that genomic islands of speciation are due to reduced diversity, not reduced gene flow. Mol. Ecol. 23:3133–3157. 24845075 10.1111/mec.12796

[evl374-bib-0011] Derryberry, E. P. , G. E. Derryberry , J. M. Maley , and R. T. Brumfield . 2014 hzar: Hybrid zone analysis using an R software package. Mol. Ecol. Resour. 14:652–663. 24373504 10.1111/1755-0998.12209

[evl374-bib-0012] Faria, R. , and A. Navarro . 2010 Chromosomal speciation revisited: rearranging theory with pieces of evidence. Trends Ecol. Evol. 25:660–669. 20817305 10.1016/j.tree.2010.07.008

[evl374-bib-0013] Felsenstein, J. 1981 Skepticism towards Santa Rosalia, or why are there so few kinds of animals? Evolution 124–138. 28563447 10.1111/j.1558-5646.1981.tb04864.x

[evl374-bib-0014] Foll, M. , and O. Gaggiotti . 2008 A genome‐scan method to identify selected loci appropriate for both dominant and codominant markers: a Bayesian perspective. Genetics 180:977–993. 18780740 10.1534/genetics.108.092221 PMC2567396

[evl374-bib-0015] Garant, D. , S. E. Forde , and A. P. Hendry . 2006 The multifarious effects of dispersal and gene flow on contemporary adaptation. Funct. Ecol. 21:434–443.

[evl374-bib-0016] Gompert, Z. , L. K. Lucas , C. C. Nice , J. A. Fordyce , M. L. Forister , and C. A. Buerkle . 2012 Genomic regions with a history of divergent selection affect fitness of hybrids between two butterfly species. Evolution 66:2167–2181. 22759293 10.1111/j.1558-5646.2012.01587.x

[evl374-bib-0017] Gompert, Z. , E. G. Mandeville , and C. A. Buerkle . 2017 Analysis of population genomic data from hybrid zones. Annu. Rev. Ecol. Evol. Syst. 48:207–229.

[evl374-bib-0018] Harrison, R. G. 1993 Hybrid zones and the evolutionary process. Oxford University Press, Oxford.

[evl374-bib-0019] Harrison, R. G. , and E. L. Larson . 2016 Heterogeneous genome divergence, differential introgression, and the origin and structure of hybrid zones. Mol. Ecol. 25:2454–2466. 26857437 10.1111/mec.13582 PMC4899261

[evl374-bib-0020] Henning, F. , G. Machado‐Schiaffino , L. Baumgarten , and A. Meyer . 2017 Genetic dissection of adaptive form and function in rapidly speciating cichlid fishes. Evolution 71:1297–1312. 28211577 10.1111/evo.13206

[evl374-bib-0021] Hollander, J. , J. Galindo , and R. K. Butlin . 2015 Selection on outlier loci and their association with adaptive phenotypes in *Littorina saxatilis* contact zones. J. Evol. Biol. 28:328–337. 25439395 10.1111/jeb.12564

[evl374-bib-0022] Janson, K. 1983a Chromosome number in two phenotypically distinct populations of *Littorina saxatilis* olivi, and in specimens of the *Littorina obtusata* (L.) species complex. J. Molluscan Stud. 49:224–227.

[evl374-bib-0023] Janson, K. 1983b Selection and migration in two distinct phenotypes of *Littorina saxatilis* in Sweden. Oecologia 59:58–61. 25024147 10.1007/BF00388072

[evl374-bib-0024] Johannesson, B. 1986 Shell morphology of *Littorina saxatilis* Olivi: The relative importance of physical factors and predation. J. Exp. Mar. Biol. Ecol. 102:183–195.

[evl374-bib-0025] Johannesson, K. , and R. K. Butlin . 2017 What explains rare and conspicuous colours in a snail? A test of time‐series data against models of drift, migration or selection. Heredity 118:21–30. 27649616 10.1038/hdy.2016.77 PMC5176118

[evl374-bib-0026] Johannesson, K. , M. Panova , P. Kemppainen , C. André , E. Rolán‐Alvarez , and R. K. Butlin . 2010 Repeated evolution of reproductive isolation in a marine snail: unveiling mechanisms of speciation. Philos. Trans. R. Soc. B Biol. Sci. 365:1735–1747. 10.1098/rstb.2009.0256 PMC2871885 20439278

[evl374-bib-0027] Jones, F. C. , M. G. Grabherr , Y. F. Chan , P. Russell , E. Mauceli , J. Johnson , et al. 2012 The genomic basis of adaptive evolution in threespine sticklebacks. Nature 484:55–61. 22481358 10.1038/nature10944 PMC3322419

[evl374-bib-0028] Kirkpatrick, M. , and N. Barton . 2006 Chromosome inversions, local adaptation and speciation. Genetics 173:419–434. 16204214 10.1534/genetics.105.047985 PMC1461441

[evl374-bib-0029] Kostem, E. , and E. Eskin . 2013 Improving the accuracy and efficiency of partitioning heritability into the contributions of genomic regions. Am. J. Hum. Genet. 92:558–564. 23561845 10.1016/j.ajhg.2013.03.010 PMC3617385

[evl374-bib-0030] Le Corre, V. , and A. Kremer . 2012 The genetic differentiation at quantitative trait loci under local adaptation. Mol. Ecol. 21:1548–1566. 22332667 10.1111/j.1365-294X.2012.05479.x

[evl374-bib-0031] Le Pennec, G. , R. K. Butlin , P. R. Jonsson , A. I. Larsson , J. Lindborg , E. Bergström , et al. 2017 Adaptation to dislodgement risk on wave‐swept rocky shores in the snail *Littorina saxatilis* . PLoS One 12:e0186901. 29059225 10.1371/journal.pone.0186901 PMC5653359

[evl374-bib-0032] Lenormand, T. 2002 Gene flow and the limits to natural selection. Trends Ecol. Evol. 17:183–189.

[evl374-bib-0033] Lindtke, D. , C. A. Buerkle , T. Barbará , B. Heinze , S. Castiglione , D. Bartha , et al. 2012 Recombinant hybrids retain heterozygosity at many loci: new insights into the genomics of reproductive isolation in *Populus* . Mol. Ecol. 21:5042–5058. 22989336 10.1111/j.1365-294X.2012.05744.x

[evl374-bib-0034] Lindtke, D. , S. C. González‐Martínez , D. Macaya‐Sanz , and C. Lexer . 2013 Admixture mapping of quantitative traits in *Populus* hybrid zones: power and limitations. Heredity 111:474. 23860234 10.1038/hdy.2013.69 PMC3833683

[evl374-bib-0035] Martin, S. H. , M. Möst , W. J. Palmer , C. Salazar , W. O. McMillan , F. M. Jiggins , et al. 2016 Natural selection and genetic diversity in the butterfly *Heliconius melpomene* . Genetics 203:525–541. 27017626 10.1534/genetics.115.183285 PMC4858797

[evl374-bib-0036] Money, D. , K. Gardner , Z. Migicovsky , H. Schwaninger , G.‐Y. Zhong , and S. Myles . 2015 Linkimpute: Fast and accurate genotype imputation for nonmodel organisms. G3 Genes Genomes Genet. 5:2383–2390. 10.1534/g3.115.021667 PMC4632058 26377960

[evl374-bib-0037] Navarro, A. , and N. H. Barton . 2003 Accumulating postzygotic isolation genes in parapatry: a new twist on chromosomal speciation. Evolution 57:447–459. 12703935 10.1111/j.0014-3820.2003.tb01537.x

[evl374-bib-0038] Noor, M. , and S. M. Bennett . 2009 Islands of speciation or mirages in the desert? Examining the role of restricted recombination in maintaining species. Heredity 103:439–444. 19920849 10.1038/hdy.2009.151 PMC2809014

[evl374-bib-0039] Nosil, P. 2012 Ecological speciation. Oxford University Press, Oxford.

[evl374-bib-0040] Panova, M. , H. Aronsson , R. A. Cameron , P. Dahl , A. Godhe , U. Lind , et al. 2016 DNA extraction protocols for whole‐genome sequencing in marine organisms Pp. 13–44 *in* Bourlat S. J., ed. *Marine Genomics*, methods in molecular biology. Springer, New York. 10.1007/978-1-4939-3774-5_2 27460368

[evl374-bib-0041] Panova, M. , J. Hollander , and K. Johannesson . 2006 Site‐specific genetic divergence in parallel hybrid zones suggests nonallopatric evolution of reproductive barriers. Mol. Ecol. 15:4021–4031. 17054500 10.1111/j.1365-294X.2006.03067.x

[evl374-bib-0042] Parchman, T. L. , Z. Gompert , M. J. Braun , R. T. Brumfield , D. B. McDonald , J. A. C. Uy , et al. 2013 The genomic consequences of adaptive divergence and reproductive isolation between species of manakins. Mol. Ecol. 22:3304–3317. 23441849 10.1111/mec.12201

[evl374-bib-0043] Pinho, C. , and J. Hey . 2010 Divergence with gene flow: models and data. Annu. Rev. Ecol. Evol. Syst. 41:215–230.

[evl374-bib-0044] Price, A. L. , N. J. Patterson , R. M. Plenge , M. E. Weinblatt , N. A. Shadick , and D. Reich . 2006 Principal components analysis corrects for stratification in genome‐wide association studies. Nat. Genet. 38:904. 16862161 10.1038/ng1847

[evl374-bib-0045] Rafajlović, M. , A. Emanuelsson , K. Johannesson , R. K. Butlin , and B. Mehlig . 2016 A universal mechanism generating clusters of differentiated loci during divergence‐with‐migration. Evolution 70:1609–1621. 27196373 10.1111/evo.12957 PMC5089645

[evl374-bib-0046] Ravinet, M. , R. Faria , R. K. Butlin , J. Galindo , N. Bierne , M. Rafajlović , et al. 2017 Interpreting the genomic landscape of speciation. J. Evol. Biol. 30:1450–1477. 28786193 10.1111/jeb.13047

[evl374-bib-0047] Ravinet, M. , A. Westram , K. Johannesson , R. Butlin , C. André , and M. Panova . 2016 Shared and nonshared genomic divergence in parallel ecotypes of *Littorina saxatilis* at a local scale. Mol. Ecol. 25:287–305. 26222268 10.1111/mec.13332

[evl374-bib-0048] Ravinet, M. , K. Yoshida , S. Shigenobu , A. Toyoda , A. Fujiyama , and J. Kitano . 2018 The genomic landscape at a late stage of stickleback speciation: High genomic divergence interspersed by small localized regions of introgression. PLoS Genet. 14:e1007358. 29791436 10.1371/journal.pgen.1007358 PMC5988309

[evl374-bib-0049] Reid, D. G. 1996 Systematics and evolution of *Littorina*. Ray Society, London.

[evl374-bib-0050] Renaut, S. , C. J. Grassa , S. Yeaman , B. T. Moyers , Z. Lai , N. C. Kane , et al. 2013 Genomic islands of divergence are not affected by geography of speciation in sunflowers. Nat. Commun. 4:1827. 23652015 10.1038/ncomms2833

[evl374-bib-0051] Rolán‐Alvarez, E. , I. Buño , and J. Gosalvez . 1996 Sex is determined by sex chromosomes in *Littorina saxatilis* (Olivi) (Gastropoda, Prosobranchia). Hereditas 124:261–268.

[evl374-bib-0052] Samuk, K. , G. L. Owens , K. E. Delmore , S. E. Miller , D. J. Rennison , and D. Schluter . 2017 Gene flow and selection interact to promote adaptive divergence in regions of low recombination. Mol. Ecol. 26:4378–4390. 28667780 10.1111/mec.14226

[evl374-bib-0053] Schluter, D. 2001 Ecology and the origin of species. Trends Ecol. Evol. 16:372–380. 11403870 10.1016/s0169-5347(01)02198-x

[evl374-bib-0054] Slatkin, M. 1973 Gene flow and selection in a cline. Genetics 75:733–756. 4778791 10.1093/genetics/75.4.733 PMC1213045

[evl374-bib-0055] Smadja, C. M. , and R. K. Butlin . 2011 A framework for comparing processes of speciation in the presence of gene flow. Mol. Ecol. 20:5123–5140. 22066935 10.1111/j.1365-294X.2011.05350.x

[evl374-bib-0056] Stankowski, S. , J. M. Sobel , and M. A. Streisfeld . 2017 Geographic cline analysis as a tool for studying genome‐wide variation: a case study of pollinator‐mediated divergence in a monkeyflower. Mol. Ecol. 26:107–122. 27065228 10.1111/mec.13645

[evl374-bib-0057] Stuart, Y. E. , T. Veen , J. N. Weber , D. Hanson , M. Ravinet , B. K. Lohman , et al. 2017 Contrasting effects of environment and genetics generate a continuum of parallel evolution. Nat. Ecol. Evol. 1:0158. 10.1038/s41559-017-0158 28812631

[evl374-bib-0058] Szymura, J. M. , and N. H. Barton . 1986 Genetic analysis of a hybrid zone between the fire‐bellied toads, *Bombina bombina* and *B. variegata*, near Cracow in southern Poland. Evolution 40:1141–1159. 28563502 10.1111/j.1558-5646.1986.tb05740.x

[evl374-bib-0059] Twyford, A. D. , and J. Friedman . 2015 Adaptive divergence in the monkey flower *Mimulus guttatus* is maintained by a chromosomal inversion. Evolution 69:1476–1486. 25879251 10.1111/evo.12663 PMC5029580

[evl374-bib-0060] Vines, T. H. , A. C. Dalziel , A. Y. K. Albert , T. Veen , P. M. Schulte , and D. Schluter . 2016 Cline coupling and uncoupling in a stickleback hybrid zone. Evolution 70:1023–1038. 27061719 10.1111/evo.12917

[evl374-bib-0061] Wellenreuther, M. , H. Rosenquist , P. Jaksons , and K. W. Larson . 2017 Local adaptation along an environmental cline in a species with an inversion polymorphism. J. Evol. Biol. 30:1068–1077. 28295810 10.1111/jeb.13064

[evl374-bib-0062] Yeaman, S. 2015 Local adaptation by alleles of small effect. Am. Nat. 186:S74–S89. 26656219 10.1086/682405

[evl374-bib-0063] Yeaman, S. , and M. C. Whitlock . 2011 The genetic architecture of adaptation under migration–selection balance. Evolution 65:1897–1911. 21729046 10.1111/j.1558-5646.2011.01269.x

