# Supplementary material for: Clines on the seashore: The genomic architecture underlying rapid divergence in the face of gene flow
Source: Evol Lett. 2018 Aug 7;2(4):297–309. doi: 10.1002/evl3.74 (PMC6121805; doi:10.1002/evl3.74)
Supplement: Supplementary file 1 — TABLE S1.1 Summary statistics of the maximum‐likelihood results for simulated allele‐frequency data at neutrally evolving loci, with the number of individuals in each patch set to N =100. [file EVL3-2-297-s001.docx]

TABLE S1.1 Summary statistics of the maximum-likelihood results for simulated allele-frequency data at neutrally evolving loci, with the number of individuals in each patch set to *N* =100.

| Neutrally Evolving Loci | | | | | | | | | | |
| --- | --- | --- | --- | --- | --- | --- | --- | --- | --- | --- |
| *σ* | Model | #Selected Loci*^a^* | Sampling Time | % Processed*^b^* | %Clinal Loci*^c^* | | | | %Non-Clinal Loci*^d^* | |
|  |  | *L* | *T* |  | Simple | Right Tail | Left Tail | Both Tails | *p*_d_ *<* 0*.*1*^e^* | *p*_d_ *>* 0*.*1 |
| *σ*  =1  *.*  46 | Model 1 | *L* = 10 | *T* = 1000 | 99.66 | 56.24 | 0.04 | 0.08 | 0.03 | 31.48 | 12.14 |
|  |  |  | *T* = 2000 | 99.42 | 60.68 | 0.09 | 0.08 | 0.04 | 28.59 | 10.52 |
|  |  |  | *T* = 4000 | 96.80 | 61.92 | 0.06 | 0.08 | 0.03 | 28.69 | 9.23 |
|  |  |  | *T* = 8000 | 88.60 | 61.64 | 0.06 | 0.09 | 0.05 | 29.36 | 8.79 |
|  |  | *L* = 50 | *T* = 1000 | 99.69 | 57.08 | 0.06 | 0.06 | 0.03 | 30.80 | 11.97 |
|  |  |  | *T* = 2000 | 99.34 | 62.14 | 0.09 | 0.08 | 0.05 | 27.46 | 10.20 |
|  |  |  | *T* = 4000 | 96.70 | 64.73 | 0.08 | 0.08 | 0.08 | 26.39 | 8.65 |
|  |  |  | *T* = 8000 | 88.57 | 64.33 | 0.11 | 0.13 | 0.04 | 27.37 | 8.02 |
|  |  | *L* = 200 | *T* = 1000 | 99.68 | 56.34 | 0.03 | 0.07 | 0.04 | 30.89 | 12.63 |
|  |  |  | *T* = 2000 | 99.42 | 63.24 | 0.09 | 0.09 | 0.04 | 26.72 | 9.81 |
|  |  |  | *T* = 4000 | 96.69 | 66.84 | 0.08 | 0.08 | 0.08 | 24.65 | 8.27 |
|  |  |  | *T* = 8000 | 88.43 | 66.58 | 0.08 | 0.13 | 0.07 | 25.70 | 7.43 |
|  | Model 2 | *L* = 10 | *T* = 1000 | 99.57 | 66.63 | 0.08 | 0.13 | 0.06 | 24.09 | 9.02 |
|  |  |  | *T* = 2000 | 98.85 | 64.48 | 0.11 | 0.11 | 0.07 | 26.35 | 8.89 |
|  |  |  | *T* = 4000 | 95.42 | 63.01 | 0.08 | 0.08 | 0.03 | 27.83 | 8.95 |
|  |  |  | *T* = 8000 | 87.08 | 61.62 | 0.08 | 0.09 | 0.04 | 29.65 | 8.51 |
|  |  | *L* = 50 | *T* = 1000 | 99.59 | 67.53 | 0.12 | 0.17 | 0.08 | 23.22 | 8.89 |
|  |  |  | *T* = 2000 | 98.65 | 67.06 | 0.11 | 0.12 | 0.06 | 24.13 | 8.52 |
|  |  |  | *T* = 4000 | 95.58 | 65.48 | 0.09 | 0.12 | 0.05 | 26.16 | 8.10 |
|  |  |  | *T* = 8000 | 87.05 | 64.30 | 0.08 | 0.13 | 0.06 | 27.59 | 7.85 |
|  |  | *L* = 200 | *T* = 1000 | 99.60 | 68.40 | 0.11 | 0.15 | 0.09 | 22.49 | 8.76 |
|  |  |  | *T* = 2000 | 98.83 | 68.11 | 0.11 | 0.10 | 0.07 | 23.48 | 8.14 |
|  |  |  | *T* = 4000 | 95.21 | 67.46 | 0.13 | 0.15 | 0.05 | 24.61 | 7.61 |
|  |  |  | *T* = 8000 | 86.68 | 67.12 | 0.10 | 0.12 | 0.07 | 25.09 | 7.51 |

*^a^*Per simulation. *^b^*Percentage of all neutral loci that have passed our filters preceding fitting the data.

*^c^*Out of all processed neutral loci. *^d^*Out of all processed neutral loci. *^e^p*_d_ denotes the difference in allele frequencies at the two habitat ends.
