# Supplementary material for: Clines on the seashore: The genomic architecture underlying rapid divergence in the face of gene flow
Source: Evol Lett. 2018 Aug 7;2(4):297–309. doi: 10.1002/evl3.74 (PMC6121805; doi:10.1002/evl3.74)
Supplement: Supplementary file 2 — TABLE S1.2 Summary statistics of the maximum‐likelihood results for simulated allele‐frequency data at loci under selection, with the number of individuals in each patch set to N =100. [file EVL3-2-297-s002.docx]

TABLE S1.2 Summary statistics of the maximum-likelihood results for simulated allele-frequency data at loci under selection, with the number of individuals in each patch set to *N* =100.

| Loci Under Selection | | | | | | | | | | |
| --- | --- | --- | --- | --- | --- | --- | --- | --- | --- | --- |
| *σ* | Model | #Selected Loci*^a^* | Sampling Time | % Processed*^b^* | %Clinal Loci*^c^* | | | | %Non-Clinal Loci*^d^* | |
|  |  | *L* | *T* |  | Simple | Right Tail | Left Tail | Both Tails | *p*_d_ *<* 0*.*1*^e^* | *p*_d_ *>* 0*.*1 |
| *σ*  =1  *.*  46 | Model 1 | *L* = 10 | *T* = 1000 | 100.00 | 76.05 | 10.75 | 10.10 | 3.10 | 0.00 | 0.00 |
|  |  |  | *T* = 2000 | 100.00 | 75.50 | 10.75 | 10.90 | 2.85 | 0.00 | 0.00 |
|  |  |  | *T* = 4000 | 100.00 | 74.45 | 11.60 | 10.95 | 3.00 | 0.00 | 0.00 |
|  |  |  | *T* = 8000 | 100.00 | 76.60 | 11.10 | 9.45 | 2.85 | 0.00 | 0.00 |
|  |  | *L* = 50 | *T* = 1000 | 100.00 | 91.31 | 2.99 | 3.63 | 2.07 | 0.00 | 0.00 |
|  |  |  | *T* = 2000 | 100.00 | 91.04 | 2.95 | 3.63 | 2.38 | 0.00 | 0.00 |
|  |  |  | *T* = 4000 | 100.00 | 91.37 | 2.75 | 3.67 | 2.21 | 0.00 | 0.00 |
|  |  |  | *T* = 8000 | 100.00 | 90.51 | 3.16 | 4.02 | 2.31 | 0.00 | 0.00 |
|  |  | *L* = 200 | *T* = 1000 | 100.00 | 99.14 | 0.31 | 0.45 | 0.10 | 0.00 | 0.00 |
|  |  |  | *T* = 2000 | 100.00 | 98.44 | 0.59 | 0.66 | 0.32 | 0.00 | 0.00 |
|  |  |  | *T* = 4000 | 100.00 | 98.28 | 0.72 | 0.71 | 0.30 | 0.00 | 0.00 |
|  |  |  | *T* = 8000 | 100.00 | 98.30 | 0.70 | 0.71 | 0.28 | 0.00 | 0.00 |
|  | Model 2 | *L* = 10 | *T* = 1000 | 100.00 | 75.90 | 11.70 | 9.90 | 2.50 | 0.00 | 0.00 |
|  |  |  | *T* = 2000 | 100.00 | 77.35 | 10.20 | 9.90 | 2.55 | 0.00 | 0.00 |
|  |  |  | *T* = 4000 | 100.00 | 74.45 | 12.00 | 10.75 | 2.80 | 0.00 | 0.00 |
|  |  |  | *T* = 8000 | 100.00 | 77.75 | 9.40 | 10.00 | 2.85 | 0.00 | 0.00 |
|  |  | *L* = 50 | *T* = 1000 | 100.00 | 91.07 | 3.18 | 3.36 | 2.39 | 0.00 | 0.00 |
|  |  |  | *T* = 2000 | 100.00 | 91.07 | 3.24 | 3.52 | 2.17 | 0.00 | 0.00 |
|  |  |  | *T* = 4000 | 100.00 | 91.16 | 2.74 | 3.69 | 2.41 | 0.00 | 0.00 |
|  |  |  | *T* = 8000 | 100.00 | 91.46 | 2.94 | 3.40 | 2.20 | 0.00 | 0.00 |
|  |  | *L* = 200 | *T* = 1000 | 100.00 | 98.13 | 0.88 | 0.72 | 0.28 | 0.00 | 0.00 |
|  |  |  | *T* = 2000 | 100.00 | 98.19 | 0.79 | 0.71 | 0.30 | 0.00 | 0.00 |
|  |  |  | *T* = 4000 | 100.00 | 98.19 | 0.83 | 0.74 | 0.25 | 0.00 | 0.00 |
|  |  |  | *T* = 8000 | 100.00 | 98.27 | 0.75 | 0.67 | 0.32 | 0.00 | 0.00 |

*^a^*Per simulation. *^b^*Percentage of all selected loci that have passed our filters preceding fitting the data.

*^c^*Out of all processed selected loci. *^d^*Out of all processed selected loci. *^e^p*_d_ denotes the difference in allele frequencies at the two habitat ends.
