# Supplementary material for: Clines on the seashore: The genomic architecture underlying rapid divergence in the face of gene flow
Source: Evol Lett. 2018 Aug 7;2(4):297–309. doi: 10.1002/evl3.74 (PMC6121805; doi:10.1002/evl3.74)
Supplement: Supplementary file 3 — TABLE S1.3 Percentiles of the variance explained by the maximum‐likelihood clinal fits for simulated neutral loci (null hypothesis), with the number of individuals in each patch set to N =100. [file EVL3-2-297-s003.docx]

TABLE S1.3 Percentiles of the variance explained by the maximum-likelihood clinal fits for simulated neutral loci (null hypothesis), with the number of individuals in each patch set to *N* =100. The variance explained for loci designated as *non-clinal* was set to 0. Loci for which the variance explained was found to be negative, or with *F*_ST_ *>* 1 were excluded. The expectation used for the results shown in the main text is shown in boldface and highlighted by a star.

| *σ* | Model | #Selected Loci*^a^* | Sampling Time | Percentile | |
| --- | --- | --- | --- | --- | --- |
|  |  | *L* | *T* | 95 | 99 |
| *σ*  =1  *.*  46 | Model 1 | *L* = 10 | *T* = 1000 | 14.13 | 21.52 |
|  |  |  | *T* = 2000 | 17.07 | 25.69 |
|  |  |  | *T* = 4000 | 19.56 | 29.16 |
|  |  |  | *T* = 8000 | 19.98 | 30.05 |
|  |  | *L* = 50 | *T* = 1000 | 14.72 | 22.31 |
|  |  |  | *T* = 2000 | 18.94 | 28.48 |
|  |  |  | *T* = 4000 | 22.41 | 33.46 |
|  |  |  | *T* = 8000 | 23.01 | 33.60 |
|  |  | *L* = 200 | *T* = 1000 | 13.93 | 21.58 |
|  |  |  | *T* = 2000 | 19.49 | 29.26 |
|  |  |  | *T* = 4000 | 24.27 | 35.69*^?^* |
|  |  |  | *T* = 8000 | 26.39 | 37.65 |
|  | Model 2 | *L* = 10 | *T* = 1000 | 20.93 | 31.16 |
|  |  |  | *T* = 2000 | 20.18 | 29.57 |
|  |  |  | *T* = 4000 | 20.17 | 29.29 |
|  |  |  | *T* = 8000 | 20.26 | 29.93 |
|  |  | *L* = 50 | *T* = 1000 | 22.92 | 34.51 |
|  |  |  | *T* = 2000 | 23.11 | 34.24 |
|  |  |  | *T* = 4000 | 23.16 | 33.95 |
|  |  |  | *T* = 8000 | 23.29 | 35.24 |
|  |  | *L* = 200 | *T* = 1000 | 23.42 | 35.40 |
|  |  |  | *T* = 2000 | 24.10 | 34.95 |
|  |  |  | *T* = 4000 | 25.16 | 37.29 |
|  |  |  | *T* = 8000 | 25.77 | 37.78 |

*^a^*Per simulation.
