# Supplementary material for: Clines on the seashore: The genomic architecture underlying rapid divergence in the face of gene flow
Source: Evol Lett. 2018 Aug 7;2(4):297–309. doi: 10.1002/evl3.74 (PMC6121805; doi:10.1002/evl3.74)
Supplement: Supplementary file 4 — TABLE S1.4 Same as in Tab. S1.1 but for two additional values of the dispersal distance σ: σ = 1.09 and σ = 1.70. [file EVL3-2-297-s004.docx]

TABLE S1.4 Same as in Tab. S1.1 but for two additional values of the dispersal distance *σ*: *σ* = 1*.*09 and *σ* = 1*.*70. In both cases, the primary divergence model (i.e. Model 1) was simulated, and the number of individuals in each patch was set to *N* =100.

| Neutrally Evolving Loci | | | | | | | | | | |
| --- | --- | --- | --- | --- | --- | --- | --- | --- | --- | --- |
| *σ* | Model | #Selected Loci*^a^* | Sampling Time | % Processed*^b^* | %Clinal Loci*^c^* | | | | %Non-Clinal Loci*^d^* | |
|  |  | *L* | *T* |  | Simple | Right Tail | Left Tail | Both Tails | *p*_d_ *<* 0*.*1*^e^* | *p*_d_ *>* 0*.*1 |
| *σ*  =1  *.*  09 | Model 1 | *L* = 10 | *T* = 1000 | 98.77 | 59.59 | 0.10 | 0.11 | 0.05 | 30.94 | 9.22 |
|  |  |  | *T* = 2000 | 98.47 | 65.14 | 0.11 | 0.17 | 0.07 | 26.89 | 7.62 |
|  |  |  | *T* = 4000 | 96.30 | 68.25 | 0.22 | 0.19 | 0.11 | 26.76 | 6.47 |
|  |  |  | *T* = 8000 | 88.84 | 68.25 | 0.25 | 0.19 | 0.11 | 25.18 | 6.03 |
|  |  | *L* = 50 | *T* = 1000 | 98.99 | 59.91 | 0.08 | 0.07 | 0.05 | 30.48 | 9.41 |
|  |  |  | *T* = 2000 | 98.59 | 66.24 | 0.17 | 0.15 | 0.07 | 25.55 | 7.82 |
|  |  |  | *T* = 4000 | 96.03 | 69.62 | 0.23 | 0.24 | 0.10 | 23.62 | 6.20 |
|  |  |  | *T* = 8000 | 88.52 | 70.43 | 0.27 | 0.27 | 0.13 | 23.43 | 5.46 |
|  |  | *L* = 200 | *T* = 1000 | 99.12 | 60.55 | 0.07 | 0.10 | 0.07 | 29.79 | 9.43 |
|  |  |  | *T* = 2000 | 98.58 | 67.37 | 0.12 | 0.15 | 0.07 | 25.11 | 7.19 |
|  |  |  | *T* = 4000 | 96.28 | 70.46 | 0.22 | 0.22 | 0.10 | 22.99 | 6.01 |
|  |  |  | *T* = 8000 | 87.85 | 72.07 | 0.30 | 0.27 | 0.11 | 22.13 | 5.11 |
| *σ*  =1  *.*  70 | Model 1 | *L* = 10 | *T* = 1000 | 99.79 | 52.82 | 0.05 | 0.06 | 0.02 | 33.31 | 13.74 |
|  |  |  | *T* = 2000 | 99.48 | 57.85 | 0.04 | 0.06 | 0.03 | 29.82 | 12.19 |
|  |  |  | *T* = 4000 | 96.97 | 58.61 | 0.06 | 0.07 | 0.02 | 29.86 | 11.37 |
|  |  |  | *T* = 8000 | 89.05 | 56.97 | 0.06 | 0.06 | 0.02 | 32.28 | 10.61 |
|  |  | *L* = 50 | *T* = 1000 | 99.86 | 54.14 | 0.03 | 0.06 | 0.03 | 32.29 | 13.45 |
|  |  |  | *T* = 2000 | 99.42 | 59.94 | 0.04 | 0.05 | 0.04 | 28.23 | 11.70 |
|  |  |  | *T* = 4000 | 96.84 | 60.73 | 0.06 | 0.07 | 0.04 | 28.56 | 10.54 |
|  |  |  | *T* = 8000 | 88.40 | 60.14 | 0.08 | 0.06 | 0.05 | 29.66 | 10.02 |
|  |  | *L* = 200 | *T* = 1000 | 99.86 | 52.93 | 0.04 | 0.06 | 0.04 | 32.71 | 14.22 |
|  |  |  | *T* = 2000 | 99.43 | 60.43 | 0.04 | 0.06 | 0.02 | 27.81 | 11.64 |
|  |  |  | *T* = 4000 | 96.94 | 62.88 | 0.06 | 0.09 | 0.05 | 26.92 | 10.00 |
|  |  |  | *T* = 8000 | 88.14 | 61.99 | 0.07 | 0.10 | 0.04 | 28.35 | 9.45 |

*^a^*Per simulation. *^b^*Percentage of all neutral loci that have passed our filters preceding fitting the data.

*^c^*Out of all processed neutral loci. *^d^*Out of all processed neutral loci. *^e^p*_d_ denotes the difference in allele frequencies at the two habitat ends.
