# Supplementary material for: Clines on the seashore: The genomic architecture underlying rapid divergence in the face of gene flow
Source: Evol Lett. 2018 Aug 7;2(4):297–309. doi: 10.1002/evl3.74 (PMC6121805; doi:10.1002/evl3.74)
Supplement: Supplementary file 5 — TABLE S1.5 Same as in Tab. S1.4 but for loci under selection. [file EVL3-2-297-s005.docx]

TABLE S1.5 Same as in Tab. S1.4 but for loci under selection.

| Loci Under Selection | | | | | | | | | | |
| --- | --- | --- | --- | --- | --- | --- | --- | --- | --- | --- |
| *σ* | Model | #Selected Loci*^a^* | Sampling Time | % Processed*^b^* | %Clinal Loci*^c^* | | | | %Non-Clinal Loci*^d^* | |
|  |  | *L* | *T* |  | Simple | Right Tail | Left Tail | Both Tails | *p*_d_ *<* 0*.*1*^e^* | *p*_d_ *>* 0*.*1 |
| *σ*  =1  *.*  09 | Model 1 | *L* = 10 | *T* = 1000 | 100.00 | 75.00 | 10.15 | 11.10 | 3.75 | 0.00 | 0.00 |
|  |  |  | *T* = 2000 | 100.00 | 75.40 | 10.35 | 11.50 | 2.75 | 0.00 | 0.00 |
|  |  |  | *T* = 4000 | 100.00 | 75.10 | 10.45 | 11.75 | 2.70 | 0.00 | 0.00 |
|  |  |  | *T* = 8000 | 100.00 | 74.55 | 11.80 | 10.80 | 2.85 | 0.00 | 0.00 |
|  |  | *L* = 50 | *T* = 1000 | 100.00 | 86.01 | 4.99 | 5.21 | 3.79 | 0.00 | 0.00 |
|  |  |  | *T* = 2000 | 100.00 | 86.19 | 4.86 | 4.83 | 4.12 | 0.00 | 0.00 |
|  |  |  | *T* = 4000 | 100.00 | 86.22 | 4.64 | 5.54 | 3.60 | 0.00 | 0.00 |
|  |  |  | *T* = 8000 | 100.00 | 85.59 | 5.08 | 5.32 | 4.01 | 0.00 | 0.00 |
|  |  | *L* = 200 | *T* = 1000 | 100.00 | 98.14 | 0.88 | 0.79 | 0.18 | 0.00 | 0.00 |
|  |  |  | *T* = 2000 | 100.00 | 95.54 | 2.35 | 1.41 | 0.70 | 0.00 | 0.00 |
|  |  |  | *T* = 4000 | 100.00 | 94.73 | 2.74 | 1.58 | 0.95 | 0.00 | 0.00 |
|  |  |  | *T* = 8000 | 100.00 | 94.82 | 2.74 | 1.53 | 0.91 | 0.00 | 0.00 |
| *σ*  =1  *.*  70 | Model 1 | *L* = 10 | *T* = 1000 | 100.00 | 78.85 | 9.50 | 8.55 | 3.10 | 0.00 | 0.00 |
|  |  |  | *T* = 2000 | 100.00 | 79.05 | 9.75 | 8.50 | 2.70 | 0.00 | 0.00 |
|  |  |  | *T* = 4000 | 100.00 | 76.25 | 10.75 | 9.40 | 3.60 | 0.00 | 0.00 |
|  |  |  | *T* = 8000 | 100.00 | 78.85 | 8.70 | 9.40 | 3.05 | 0.00 | 0.00 |
|  |  | *L* = 50 | *T* = 1000 | 100.00 | 92.79 | 2.48 | 3.01 | 1.72 | 0.00 | 0.00 |
|  |  |  | *T* = 2000 | 100.00 | 93.17 | 2.55 | 2.81 | 1.47 | 0.00 | 0.00 |
|  |  |  | *T* = 4000 | 100.00 | 93.75 | 2.40 | 2.58 | 1.27 | 0.00 | 0.00 |
|  |  |  | *T* = 8000 | 100.00 | 93.26 | 2.49 | 2.75 | 1.50 | 0.00 | 0.00 |
|  |  | *L* = 200 | *T* = 1000 | 100.00 | 99.46 | 0.20 | 0.30 | 0.05 | 0.00 | 0.00 |
|  |  |  | *T* = 2000 | 100.00 | 99.18 | 0.24 | 0.46 | 0.12 | 0.00 | 0.00 |
|  |  |  | *T* = 4000 | 100.00 | 99.10 | 0.28 | 0.46 | 0.16 | 0.00 | 0.00 |
|  |  |  | *T* = 8000 | 100.00 | 99.10 | 0.24 | 0.47 | 0.18 | 0.00 | 0.00 |

*^a^*Per simulation. *^b^*Percentage of all neutral loci that have passed our filters preceding fitting the data.

*^c^*Out of all processed neutral loci. *^d^*Out of all processed neutral loci. *^e^p*_d_ denotes the difference in allele frequencies at the two habitat ends.
