# Supplementary material for: Clines on the seashore: The genomic architecture underlying rapid divergence in the face of gene flow
Source: Evol Lett. 2018 Aug 7;2(4):297–309. doi: 10.1002/evl3.74 (PMC6121805; doi:10.1002/evl3.74)
Supplement: Supplementary file 6 — TABLE S1.6 Same as in Tab. S1.3 but for two additional values of the dispersal distance σ: σ = 1.09 and σ = 1.70. [file EVL3-2-297-s006.docx]

TABLE S1.6 Same as in Tab. S1.3 but for two additional values of the dispersal distance *σ*: *σ* = 1*.*09 and *σ* = 1*.*70. In both cases, the primary divergence model (i.e. Model 1) was simulated, and the number of individuals in each patch set to *N* =100.

| *σ* | Model | #Selected Loci*^a^* | Sampling Time | Percentile | |
| --- | --- | --- | --- | --- | --- |
|  |  | *L* | *T* | 95 | 99 |
| *σ*  =1  *.*  09 | Model 1 | *L* = 10 | *T* = 1000 | 16.70 | 25.38 |
|  |  |  | *T* = 2000 | 21.54 | 31.48 |
|  |  |  | *T* = 4000 | 25.95 | 37.25 |
|  |  |  | *T* = 8000 | 27.58 | 39.22 |
|  |  | *L* = 50 | *T* = 1000 | 16.86 | 25.37 |
|  |  |  | *T* = 2000 | 22.86 | 33.92 |
|  |  |  | *T* = 4000 | 27.91 | 40.40 |
|  |  |  | *T* = 8000 | 30.95 | 43.92 |
|  |  | *L* = 200 | *T* = 1000 | 16.93 | 25.66 |
|  |  |  | *T* = 2000 | 23.20 | 34.17 |
|  |  |  | *T* = 4000 | 30.01 | 43.25 |
|  |  |  | *T* = 8000 | 35.04 | 47.48 |
| *σ*  =1  *.*  70 | Model 1 | *L* = 10 | *T* = 1000 | 12.68 | 19.42 |
|  |  |  | *T* = 2000 | 15.31 | 22.87 |
|  |  |  | *T* = 4000 | 16.79 | 25.29 |
|  |  |  | *T* = 8000 | 16.27 | 25.07 |
|  |  | *L* = 50 | *T* = 1000 | 13.36 | 20.81 |
|  |  |  | *T* = 2000 | 17.30 | 25.61 |
|  |  |  | *T* = 4000 | 19.21 | 29.33 |
|  |  |  | *T* = 8000 | 19.70 | 29.00 |
|  |  | *L* = 200 | *T* = 1000 | 12.60 | 19.26 |
|  |  |  | *T* = 2000 | 17.28 | 25.54 |
|  |  |  | *T* = 4000 | 20.42 | 30.23 |
|  |  |  | *T* = 8000 | 20.73 | 31.66 |

*^a^*Per simulation.
