# Supplementary material for: Clines on the seashore: The genomic architecture underlying rapid divergence in the face of gene flow
Source: Evol Lett. 2018 Aug 7;2(4):297–309. doi: 10.1002/evl3.74 (PMC6121805; doi:10.1002/evl3.74)
Supplement: Supplementary file 7 — TABLE S1.7 Same as in Tab. S1.1 but for two additional values of the local population size N: N = 50 and N = 200. [file EVL3-2-297-s007.docx]

TABLE S1.7 Same as in Tab. S1.1 but for two additional values of the local population size *N*: *N* = 50 and *N* = 200. In both cases, the primary divergence model (i.e. Model 1) with *σ* =1*.*46 and *L* =200 was simulated.

|  | | Neutrally Evolving Loci | | | | | | | | |
| --- | --- | --- | --- | --- | --- | --- | --- | --- | --- | --- |
| *N* | Model | #Selected Loci*^a^* | Sampling Time | % Processed*^b^* | %Clinal Loci*^c^* | | | | %Non-Clinal Loci*^d^* | |
|  |  | *L* | *T* |  | Simple | Right Tail | Left Tail | Both Tails | *p*_d_ *<* 0*.*1*^e^* | *p*_d_ *>* 0*.*1 |
| *N*  =50 | Model 1 | *L* = 200 | *T* = 1000 | 98.38 | 67.61 | 0.17 | 0.15 | 0.10 | 24.85 | 7.12 |
|  |  |  | *T* = 2000 | 95.99 | 71.98 | 0.30 | 0.31 | 0.10 | 21.88 | 5.43 |
|  |  |  | *T* = 4000 | 88.02 | 73.65 | 0.44 | 0.37 | 0.18 | 20.88 | 4.48 |
|  |  |  | *T* = 8000 | 72.48 | 72.31 | 0.44 | 0.35 | 0.18 | 22.78 | 3.94 |
| *N*  =200 | Model 1 | *L* = 200 | *T* = 1000 | 100.00 | 41.13 | 0.01 | 0.02 | 0.02 | 39.76 | 19.07 |
|  |  |  | *T* = 2000 | 99.94 | 49.90 | 0.03 | 0.03 | 0.02 | 33.43 | 16.60 |
|  |  |  | *T* = 4000 | 99.48 | 55.04 | 0.02 | 0.04 | 0.02 | 30.45 | 14.44 |
|  |  |  | *T* = 8000 | 96.67 | 55.63 | 0.05 | 0.04 | 0.02 | 30.95 | 13.31 |

*^a^*Per simulation. *^b^*Percentage of all neutral loci that have passed our filters preceding fitting the data.

*^c^*Out of all processed neutral loci. *^d^*Out of all processed neutral loci. *^e^p*_d_ denotes the difference in allele frequencies at the two habitat ends.
