# Supplementary material for: Clines on the seashore: The genomic architecture underlying rapid divergence in the face of gene flow
Source: Evol Lett. 2018 Aug 7;2(4):297–309. doi: 10.1002/evl3.74 (PMC6121805; doi:10.1002/evl3.74)
Supplement: Supplementary file 8 — TABLE S1.8 Same as in Tab. S1.7 but for loci under selection. [file EVL3-2-297-s008.docx]

TABLE S1.8 Same as in Tab. S1.7 but for loci under selection.

|  | | Loci Under Selection | | | | | | | | |
| --- | --- | --- | --- | --- | --- | --- | --- | --- | --- | --- |
| *N* | Model | #Selected Loci*^a^* | Sampling Time | % Processed*^b^* | %Clinal Loci*^c^* | | | | %Non-Clinal Loci*^d^* | |
|  |  | *L* | *T* |  | Simple | Right Tail | Left Tail | Both Tails | *p*_d_ *<* 0*.*1*^e^* | *p*_d_ *>* 0*.*1 |
| *N*  =50 | Model 1 | *L* = 200 | *T* = 1000 | 100.00 | 97.89 | 1.12 | 0.77 | 0.20 | 0.01 | 0.00 |
|  |  |  | *T* = 2000 | 100.00 | 97.14 | 1.44 | 0.94 | 0.47 | 0.00 | 0.00 |
|  |  |  | *T* = 4000 | 100.00 | 96.84 | 1.66 | 0.94 | 0.56 | 0.00 | 0.00 |
|  |  |  | *T* = 8000 | 100.00 | 96.84 | 1.66 | 0.92 | 0.58 | 0.00 | 0.00 |
| *N*  =200 | Model 1 | *L* = 200 | *T* = 1000 | 100.00 | 99.62 | 0.10 | 0.22 | 0.06 | 0.00 | 0.00 |
|  |  |  | *T* = 2000 | 100.00 | 99.11 | 0.20 | 0.52 | 0.17 | 0.00 | 0.00 |
|  |  |  | *T* = 4000 | 100.00 | 98.93 | 0.32 | 0.53 | 0.22 | 0.00 | 0.00 |
|  |  |  | *T* = 8000 | 100.00 | 98.91 | 0.35 | 0.54 | 0.21 | 0.00 | 0.00 |

*^a^*Per simulation.

*^b^*Percentage of all neutral loci that have passed our filters preceding fitting the data.

*^c^*Out of all processed neutral loci. *^d^*Out of all processed neutral loci. *^e^p*_d_ denotes the difference in allele frequencies at the two habitat ends.
