# Supplementary material for: Clines on the seashore: The genomic architecture underlying rapid divergence in the face of gene flow
Source: Evol Lett. 2018 Aug 7;2(4):297–309. doi: 10.1002/evl3.74 (PMC6121805; doi:10.1002/evl3.74)
Supplement: Supplementary file 9 — TABLE S1.9 Same as in Tab. S1.3 but for two additional values of the local population size N: N = 50 and N = 200. [file EVL3-2-297-s009.docx]

TABLE S1.9 Same as in Tab. S1.3 but for two additional values of the local population size *N*: *N* = 50 and *N* = 200. In both cases, the primary divergence model (i.e. Model 1) with *σ* =1*.*46 and *L* =200 was simulated.

| *N* | Model | #Selected Loci*^a^* | Sampling Time | Percentile | |
| --- | --- | --- | --- | --- | --- |
|  |  | *L* | *T* | 95 | 99 |
| *N*  =50 | Model 1 | *L* = 200 | *T* = 1000 | 23.50 | 34.67 |
|  |  |  | *T* = 2000 | 31.61 | 45.01 |
|  |  |  | *T* = 4000 | 38.01 | 52.54 |
|  |  |  | *T* = 8000 | 41.04 | 55.72 |
| *N*  =200 | Model 1 | *L* = 200 | *T* = 1000 | 8.43 | 13.21 |
|  |  |  | *T* = 2000 | 11.63 | 18.17 |
|  |  |  | *T* = 4000 | 14.17 | 21.87 |
|  |  |  | *T* = 8000 | 15.47 | 23.55 |

*^a^*Per simulation.
