# Supplementary material for: Clines on the seashore: The genomic architecture underlying rapid divergence in the face of gene flow
Source: Evol Lett. 2018 Aug 7;2(4):297–309. doi: 10.1002/evl3.74 (PMC6121805; doi:10.1002/evl3.74)
Supplement: Supplementary file 10 — Table S1: Summary of sequencing libraries used for Littorina saxatilis genome assembly. [file EVL3-2-297-s010.docx]

**Table S1**: Summary of sequencing libraries used for *Littorina saxatilis* assembly.

| Library | Average insert size (bp) | Read length (bp) | GC content | Total number  of bases | Trimming method |
| --- | --- | --- | --- | --- | --- |
| Littorina_1 | 112 | 101 | 42% | 46,912,679,172 | Trim Galore/Cutadapt |
| Littorina_2 | 203 | 101 | 42% | 14,208,904,220 | Trim Galore/Cutadapt |
| Littorina_3 | 206 | 101 | 42% | 30,439,678,354 | Trim Galore/Cutadapt |
| Littorina_4 | 208 | 101 | 41% | 20,848,408,890 | Trim Galore/Cutadapt |
| Littorina_5 | 209 | 101 | 42% | 37,976,024,442 | Trim Galore/Cutadapt |
| Littorina_6 | 345 | 126 | 41% | 68,613,002,892 | Trimmomatic |
| Littorina_7 | 355 | 126 | 41% | 56,077,755,300 | Trimmomatic |
| Littorina_8 | 520 | 300 | 42% | 101,061,858,282 | Trimmomatic |
| Littorina_9 | 528 | 300 | 42% | 74,348,777,104 | Trimmomatic |
| Littorina_10 | 1329 | 101 | 39% | 29,101,813,568 | Trim Galore/Cutadapt |
| Littorina_11 | 2564 | 101 | 41% | 37,689,441,992 | Trimmomatic |
| Littorina_12 | 5814 | 101 | 41% | 29,445,574,340 | Trimmomatic |
| Littorina_PB | 3,079* | 50-33,843 | 43% | 25,327,725,638 | Length cutoff 500bp |

* = Mean subread length given for PacBio data
