# Supplementary material for: Clines on the seashore: The genomic architecture underlying rapid divergence in the face of gene flow
Source: Evol Lett. 2018 Aug 7;2(4):297–309. doi: 10.1002/evl3.74 (PMC6121805; doi:10.1002/evl3.74)
Supplement: Supplementary file 11 — Table S2: Summary of scaffolded genome assembly. [file EVL3-2-297-s011.docx]

**Table S2**: Summary of scaffolded genome assembly.

| Number of scaffolds: | 116,262 |
| --- | --- |
| N50 scaffolds: | 44,284 bp |
| NG50 scaffolds: | 55,450 bp |
| Average scaffold length: | 13,834 |
| Assembly length: | 1,608,389,317 |
| Gap content: | 4.8% |
| Min sequence length: | 1,000 |
| Max sequence length | 608,273 |
