# Supplementary material for: Clines on the seashore: The genomic architecture underlying rapid divergence in the face of gene flow
Source: Evol Lett. 2018 Aug 7;2(4):297–309. doi: 10.1002/evl3.74 (PMC6121805; doi:10.1002/evl3.74)
Supplement: Supplementary file 12 — Table S3: Summary of BUSCO analyses of scaffolded assembly. [file EVL3-2-297-s012.docx]

**Table S3**: Summary of BUSCO analyses of scaffolded assembly.

|  | Eukaryotic reference set | Metazoan reference set |
| --- | --- | --- |
| Complete BUSCOs | 239 (78.9%) | 784 (80.1%) |
| Complete and single-copy | 234 (77.2%) | 772 (78.9%) |
| Complete and duplicated | 5 (1.7%) | 12 (1.2%) |
| Fragmented BUSCOs | 31 (10.2%) | 111 (11.3%) |
| Missing BUSCOs | 33 (10.9%) | 83 (8.6%) |
| Total BUSCO groups | 303 | 978 |
