# Supplementary material for: Clines on the seashore: The genomic architecture underlying rapid divergence in the face of gene flow
Source: Evol Lett. 2018 Aug 7;2(4):297–309. doi: 10.1002/evl3.74 (PMC6121805; doi:10.1002/evl3.74)
Supplement: Supplementary file 13 — Table S4: Parameter estimates for size and shape clines. [file EVL3-2-297-s013.docx]

**Table S4**: Parameter estimates (on the transformed scale) for size and shape clines. See Methods S1 for definitions of parameters. *hos* – height on shore. For centroid size, the best fitting model included a size difference between the sexes and an effect of shore height, both of which varied clinally (∆AIC = 38.55 compared to the next best model, which did not include the shore height effect; Fig. S1). For shape, the best fitting model included an influence of size, but not sex or height on the shore (∆AIC = 110.92 compared to the model with no size effect; Fig. S2). The size effect is consistent with previous observation of allometry that differs between ecotypes (Hollander *et al.* 2006). For size, the fit was improved by allowing variance to change along the transect, including an elevation at the cline centre, but variance was constant for shape.

| **Parameter** | **Size estimate** | **Standard error** | **Shape estimate** | **Standard error** |
| --- | --- | --- | --- | --- |
| *c* | 88.26 | 0.98 | 70.96 | 0.85 |
| *w* | 29.01 | 2.33 | 7.15 | 1.98 |
| *z_crab_* | 0.258 | 0.011 | 0.270 | 0.024 |
| *z_wave_* | 0.850 | 0.024 | 0.392 | 0.020 |
| *√v_crab_* | 0.060 | 0.0057 | 0.116 | 0.0037 |
| *√v_hybrid_* | 0.156 | 0.0117 | - | - |
| *√v_wave_* | 0.119 | 0.0092 | - | - |
| *b_crab_ (sex)* | -0.059 | 0.011 | - | - |
| *b_wave_ (sex)* | -0.151 | 0.025 | - | - |
| *b_crab_ (size)* | - | - | 0.077 | 0.123 |
| *b_wave_ (size)* | - | - | 0.436 | 0.032 |
| *b_crab_ (hos)* | 0.458 | 0.065 | - | - |
| *b_wave_ (hos)* | -0.077 | 0.109 | - | - |
