# Supplementary material for: Clines on the seashore: The genomic architecture underlying rapid divergence in the face of gene flow
Source: Evol Lett. 2018 Aug 7;2(4):297–309. doi: 10.1002/evl3.74 (PMC6121805; doi:10.1002/evl3.74)
Supplement: Supplementary file 14 — Table S5: Parameter estimates for colour clines. [file EVL3-2-297-s014.docx]

**Table S5**: Parameter estimates (frequency on logit scale for *z* unless fixed at 0 or 1) for colour clines. See Methods S1 for definitions of parameters. NA – standard error could not be estimated. No fit was obtained for the ‘Other’ category. The overall frequencies of the different shell colour categories were: Beige 0.485, Dark beige 0.211, Banded 0.082, Black 0.075, Other 0.146 (based on 478 adult snails). Beige was dominant in the crab environment, while colour was much more variable in the wave environment.

| **Parameter** | **Beige estimate** | **Standard error** | **Dark beige estimate** | **Standard error** | **Black estimate** | **Standard error** | **Banded estimate** | **Standard error** |
| --- | --- | --- | --- | --- | --- | --- | --- | --- |
| *c* | 91.6 | NA | 68.0 | 12.8 | 100.0 | 8.00 | 94.8 | 2.28 |
| *w* | 0.46 | NA | 91.5 | 33.9 | 28.5 | 13.1 | 12.25 | 3.57 |
| *z_crab_* | 1.00 | NA | Set to 0 |  | Set to 0 |  | Set to 0 |  |
| *z_wave_* | Set to 0 |  | -0.65 | 0.32 | -0.917 | 0.384 | -0.99 | 0.22 |
