# Supplementary material for: Clines on the seashore: The genomic architecture underlying rapid divergence in the face of gene flow
Source: Evol Lett. 2018 Aug 7;2(4):297–309. doi: 10.1002/evl3.74 (PMC6121805; doi:10.1002/evl3.74)
Supplement: Supplementary file 15 — Table S6: Different categories of SNPs based on cline analysis and simulations testing for neutrality. [file EVL3-2-297-s015.docx]

**Table S6**: Different categories of SNPs based on cline analysis and simulations testing for neutrality. SNPs in grey were excluded from all downstream analyses. SNPs with small end frequency differences or without significant clinal change were categorised as neutral SNPs. SNPs with significant clines that did not deviate from neutral patterns observed in simulations were categorised as neutral as well. Only SNPs with significant clines that deviated from neutral patterns in simulations were labelled as non-neutral. SNPs “not clearly associated with any category” are based on inconsistency between jittered replicate cline fits for the same SNP, see Methods S5.

| **category (initial filters and cline analysis)** | **category (neutrality test based on sim.)** | **category in downstream analyses** | **number of SNPs** | **% of SNPs** |
| --- | --- | --- | --- | --- |
| sex-linked | NA | excluded | 2,275 | 1.55 |
| excess heterozygotes |  |  | 2,473 | 1.69 |
| central allele frequency peak |  |  | 23 | 0.02 |
| not clearly associated with any category |  |  | 3,882 | 2.65 |
| no clinal change (allele freq. difference < 0.1 or cline fit not significant) |  | neutral | 62,456 | 42.58 |
| significant cline | consistent with neutrality |  | 73,671 | 50.23 |
|  | not consistent with neutrality | **non-neutral** | 1,891 | 1.29 |
