# Supplementary material for: Clines on the seashore: The genomic architecture underlying rapid divergence in the face of gene flow
Source: Evol Lett. 2018 Aug 7;2(4):297–309. doi: 10.1002/evl3.74 (PMC6121805; doi:10.1002/evl3.74)
Supplement: Supplementary file 16 — Table S7: Initial values, lower bounds and upper bounds for maximum likelihood estimation of SNP clines. [file EVL3-2-297-s016.docx]

**Table S7**: Initial values, lower bounds and upper bounds for maximum likelihood estimation of SNP clines.

| Parameter | Description | Initial value* | Lower bound | Upper bound |
| --- | --- | --- | --- | --- |
| *p_crab_* | Crab end frequency | *e_c_* – initial estimate of Crab end frequency | 0.001 | 0.9 |
| *p_diff_* | Wave end frequency – Crab end frequency | *e_w_ - e_c_* , where *e_w_* is the initial estimate of Wave end frequency | 0.01 | 0.999-0.5*e_c_* |
| *c* | cline centre | Initial estimate of centre position – point of maximum heterozygote frequency | 1 | 150 |
| *w* | cline width | Initial estimate of width (Σ3*pq)*, based on division into 10m demes | 1 | 1.5*min(150-*c*, *c*-1) |
| *le* | ln(sequencing error) | -5 | -10 | -1 |
| *d_L_* | distance of the introgression tail to the left from the centre of the cline | *w* | 0 | 2*w* |
| *d_R_* | distance of the introgression tail to the right from the centre of the cline | *w* | 0 | 2*w* |
| τ_L_ | ratio of the slope of left tail to the slope of the central sigmoid function at the transition point | 0.999 | 0.001 | 1 |
| τ_R_ | ratio of the slope of right tail to the slope of the central sigmoid function at the transition point | 0.999 | 0.001 | 1 |

* The simple cline fit (first 5 parameters) was run twice. In the second run these initial values were replaced with the estimates from the first run. For the 5 common parameters, tailed cline fits were initiated with parameter estimates from the simple cline.
