# Supplementary material for: Clines on the seashore: The genomic architecture underlying rapid divergence in the face of gene flow
Source: Evol Lett. 2018 Aug 7;2(4):297–309. doi: 10.1002/evl3.74 (PMC6121805; doi:10.1002/evl3.74)
Supplement: Supplementary file 17 — Table S8: Linkage disequilibrium (absolute correlation coefficient) between SNPs on linkage groups with regions showing high concentrations of non‐neutral SNPs. [file EVL3-2-297-s017.docx]

**Table S8**: Linkage disequilibrium (absolute correlation coefficient) between SNPs on linkage groups with regions showing high concentrations of non-neutral SNPs. Results are shown separately for SNPs inside and outside nnBlocks. The results demonstrate that linkage disequilibrium is elevated within nnBlocks in almost all cases (an exception is LG14 in the Wave ecotype), consistent with nnBlocks representing chromosomal rearrangements.

|  |  |  |  | **Mean and standard error of linkage disequilibrium** | | | | | |
| --- | --- | --- | --- | --- | --- | --- | --- | --- | --- |
| **LG** | **Ecotype** | **nnBlock** | **Number of SNPs** | **Between contigs, within map position** | **SE** | **Between map positions, within 5cM** | **SE** | **Between map positions, >5cM** | **SE** |
|  |  |  |  |  |  |  |  |  |  |
| 17 | Crab | Within | 270 | 0.166 | 0.0035 | 0.0740 | 0.00050 | 0.0700 | 0.00038 |
| 17 | Crab | Outside | 1553 | 0.134 | 0.00072 | 0.0787 | 0.00015 | 0.0699 | 5.4E-05 |
| 17 | Wave | Within | 323 | 0.445 | 0.0041 | 0.415 | 0.0020 | 0.415 | 0.0017 |
| 17 | Wave | Outside | 1313 | 0.208 | 0.00092 | 0.168 | 0.00032 | 0.145 | 0.00013 |
|  |  |  |  |  |  |  |  |  |  |
| 14 | Crab | Within | 1244 | 0.290 | 0.00079 | 0.259 | 0.00041 | 0.175 | 0.00035 |
| 14 | Crab | Outside | 550 | 0.180 | 0.0021 | 0.102 | 0.00043 | 0.083 | 0.00020 |
| 14 | Wave | Within | 851 | 0.198 | 0.00065 | 0.171 | 0.00031 | 0.162 | 0.00033 |
| 14 | Wave | Outside | 461 | 0.293 | 0.0028 | 0.283 | 0.0011 | 0.209 | 0.00059 |
|  |  |  |  |  |  |  |  |  |  |
| 6 | Crab | Within | 628 | 0.259 | 0.0021 | 0.273 | 0.0012 | 0.261 | 0.00064 |
| 6 | Crab | Outside | 1355 | 0.121 | 0.00049 | 0.0762 | 0.0001 | 0.0693 | 7.59E-05 |
| 6 | Wave | Within | 502 | 0.347 | 0.0026 | 0.341 | 0.0017 | 0.317 | 0.00089 |
| 6 | Wave | Outside | 1124 | 0.215 | 0.00075 | 0.171 | 0.00025 | 0.138 | 0.00018 |
