# Supplementary material for: Clines on the seashore: The genomic architecture underlying rapid divergence in the face of gene flow
Source: Evol Lett. 2018 Aug 7;2(4):297–309. doi: 10.1002/evl3.74 (PMC6121805; doi:10.1002/evl3.74)
Supplement: Supplementary file 18 — Table S9: SNPs significantly associated with colour traits according to GenABEL analysis. [file EVL3-2-297-s018.docx]

**Table S9**: SNPs significantly associated with colour traits according to GenABEL analysis. Analyses included correction for population structure (first 4 principal components), and p-values were obtained by permutation. The position of the closest SNP in the genetic map is indicated for each colour-associated SNP. Linkage groups / positions in grey indicate that the closest SNP in the linkage map is more than 1000bp away. All other SNPs are included in Fig. S4. Note that only one colour-associated SNP was also included in the cline analysis (Contig77167, Position 4379); this SNP did not show a significant cline (column ”Type”).

| **Trait** | **Contig** | **Position** | **Effect size** | **Standard error of effect size** | **PVE** | **P** | **Linkage group** | **cM** | **non-neutral SNPs in contig** | **total SNPs in contig** | **Type** |
| --- | --- | --- | --- | --- | --- | --- | --- | --- | --- | --- | --- |
| Banded | Contig150 | 124988 | 0.001715 | 0.000252 | 0.124919 | 0.009 | 12 | 40.54 | 0 | 11 | NA |
| Banded | Contig151787 | 234 | -0.00236 | 0.000376 | 0.106403 | 0.043 | 10 | 32.79 | 0 | 11 | NA |
| Banded | Contig163226 | 1342 | -0.00228 | 0.000357 | 0.110071 | 0.033 | 6 | 34.68 | 0 | 9 | NA |
| Banded | Contig190468 | 19669 | -0.00342 | 0.000427 | 0.173266 | 0.000999 | 6 | 29.58 | 0 | 12 | NA |
| Banded | Contig190468 | 19706 | -0.00336 | 0.00044 | 0.157484 | 0.000999 | 6 | 29.58 | 0 | 12 | NA |
| Banded | Contig2990 | 48679 | 0.001643 | 0.000244 | 0.122317 | 0.011 | 12 | 37.98 | 0 | 1 | NA |
| Banded | Contig3622 | 25308 | -0.0015 | 0.000191 | 0.165487 | 0.000999 | 11 | 52.91 | 0 | 19 | NA |
| Banded | Contig38854 | 11475 | -0.00321 | 0.000339 | 0.241691 | 0.000999 | 6 | 29.58 | 0 | 7 | NA |
| Banded | Contig38912 | 22856 | -0.00339 | 0.000425 | 0.171681 | 0.000999 | 6 | 29.30 | 0 | 4 | NA |
| Banded | Contig3925 | 23712 | -0.00449 | 0.000537 | 0.188606 | 0.000999 | 6 | 29.30 | 0 | 6 | NA |
| Banded | Contig3925 | 23798 | -0.00429 | 0.000306 | 0.530433 | 0.000999 | 6 | 29.30 | 0 | 6 | NA |
| Banded | Contig41890 | 22650 | -0.00293 | 0.000301 | 0.255895 | 0.000999 | 6 | 29.87 | 0 | 21 | NA |
| Banded | Contig41890 | 54571 | -0.0029 | 0.000279 | 0.292459 | 0.000999 | 6 | 29.87 | 0 | 21 | NA |
| Banded | Contig41890 | 54655 | -0.00294 | 0.000408 | 0.140276 | 0.001 | 6 | 29.87 | 0 | 21 | NA |
| Banded | Contig41890 | 55476 | -0.0029 | 0.000279 | 0.292459 | 0.000999 | 6 | 29.87 | 0 | 21 | NA |
| Banded | Contig41890 | 55491 | -0.0029 | 0.000279 | 0.292459 | 0.000999 | 6 | 29.87 | 0 | 21 | NA |
| Banded | Contig47764 | 93378 | -0.00126 | 0.000193 | 0.114966 | 0.02 | 6 | 55.27 | 0 | 4 | NA |
| Banded | Contig47764 | 93410 | -0.00126 | 0.000193 | 0.114966 | 0.02 | 6 | 55.27 | 0 | 4 | NA |
| Banded | Contig51857 | 7306 | -0.00198 | 0.000271 | 0.14346 | 0.001 | 6 | 31.00 | 0 | 11 | NA |
| Banded | Contig531 | 59764 | -0.003 | 0.000367 | 0.180947 | 0.000999 | 6 | 29.87 | 7 | 40 | NA |
| Banded | Contig531 | 59802 | -0.003 | 0.000367 | 0.180947 | 0.000999 | 6 | 29.87 | 7 | 40 | NA |
| Banded | Contig59002 | 1776 | 0.002248 | 0.000354 | 0.108831 | 0.037 | 12 | 45.82 | 0 | 4 | NA |
| Banded | Contig61068 | 52838 | -0.00208 | 0.000329 | 0.108504 | 0.037 | 6 | 37.96 | 0 | 2 | NA |
| Banded | Contig61716 | 17714 | -0.00185 | 0.000293 | 0.107423 | 0.042 | 6 | 38.91 | 0 | 1 | NA |
| Banded | Contig66881 | 44068 | -0.00239 | 0.000311 | 0.160171 | 0.000999 | 6 | 36.73 | 0 | 22 | NA |
| Banded | Contig67730 | 4971 | -0.00181 | 0.000286 | 0.108492 | 0.037 | 10 | 2.84 | 0 | 20 | NA |
| Banded | Contig69010 | 14777 | -0.00137 | 0.000174 | 0.168503 | 0.000999 | 11 | 52.91 | 0 | 11 | NA |
| Banded | Contig71895 | 26691 | -0.00299 | 0.000315 | 0.243247 | 0.000999 | 6 | 31.00 | 0 | 8 | NA |
| Banded | Contig71895 | 26692 | -0.00299 | 0.000315 | 0.243247 | 0.000999 | 6 | 31.00 | 0 | 8 | NA |
| Banded | Contig75665 | 12997 | -0.00234 | 0.000305 | 0.158341 | 0.000999 | 16 | 3.44 | 0 | 6 | NA |
| Banded | Contig77167 | 4379 | -0.00408 | 0.000562 | 0.142973 | 0.001 | 6 | 29.87 | 0 | 7 | no cline |
| Banded | Contig81952 | 1126 | -0.00198 | 0.000271 | 0.14346 | 0.001 | 6 | 31.00 | 0 | 1 | NA |
| Beige | Contig67175 | 7477 | 0.004411 | 0.00066 | 0.12078 | 0.001 | 5 | 21.93 | 0 | 3 | NA |
| Beige | Contig67175 | 7614 | 0.004411 | 0.00066 | 0.12078 | 0.001 | 5 | 21.93 | 0 | 3 | NA |
| Beige | Contig67175 | 7635 | 0.004411 | 0.00066 | 0.12078 | 0.001 | 5 | 21.93 | 0 | 3 | NA |
| Black | Contig10718 | 6069 | -0.00105 | 0.000151 | 0.131594 | 0.021 | 9 | 46.27 | 0 | 4 | NA |
