# Supplementary material for: Clines on the seashore: The genomic architecture underlying rapid divergence in the face of gene flow
Source: Evol Lett. 2018 Aug 7;2(4):297–309. doi: 10.1002/evl3.74 (PMC6121805; doi:10.1002/evl3.74)
Supplement: Supplementary file 19 — Table S10: Contributions of linkage groups to the heritability of shell size and shape, estimated in HEIDI. [file EVL3-2-297-s019.docx]

**Table S10**: Contributions of linkage groups to the heritability of shell size and shape, estimated in HEIDI. The normalised contribution is the estimated proportion of variance attributable to SNPs on a linkage group. Significance was estimated by comparing the log-likelihood of a model with only a relatedness matrix derived from all SNPs to one that also including relatedness based on the specified linkage group, tested using χ^2^ = -2∆LL (1 d.f.).

|  |  | Shell size | | | Shell shape | | |
| --- | --- | --- | --- | --- | --- | --- | --- |
| Linkage group | Number of SNPs | Normalized contribution | -2∆LL | P-value | Normalized contribution | -2∆LL | P-value |
| 1 | 2717 | 0.014 | 0.007 | 0.932 | 0.021 | 0.252 | 0.616 |
| 2 | 1914 | <0.001 | 1.895 | 0.168 | <0.001 | 1.286 | 0.257 |
| 3 | 1486 | <0.001 | 1.581 | 0.208 | 0.050 | 0.086 | 0.769 |
| 4 | 1530 | <0.001 | 0.946 | 0.330 | 0.044 | 0.080 | 0.778 |
| 5 | 1216 | 0.019 | 1.072 | 0.300 | <0.001 | 1.964 | 0.161 |
| 6 | 1093 | 0.048 | 4.019 | 0.045 | 0.112 | 3.948 | 0.047 |
| 7 | 966 | <0.001 | 0.962 | 0.326 | 0.003 | 0.116 | 0.733 |
| 8 | 893 | <0.001 | 1.262 | 0.261 | <0.001 | 0.331 | 0.565 |
| 9 | 950 | <0.001 | 0.910 | 0.340 | 0.112 | 2.758 | 0.097 |
| 10 | 884 | <0.001 | 1.396 | 0.237 | 0.009 | 0.110 | 0.740 |
| 11 | 1113 | <0.001 | 0.403 | 0.525 | 0.016 | 0.001 | 0.974 |
| 12 | 973 | 0.121 | 59.020 | <0.001 | 0.054 | 0.872 | 0.350 |
| 13 | 829 | <0.001 | 0.692 | 0.405 | 0.020 | 0.006 | 0.939 |
| 14 | 638 | 0.017 | 0.877 | 0.349 | 0.075 | 0.525 | 0.469 |
| 15 | 684 | <0.001 | 0.748 | 0.387 | <0.001 | 0.485 | 0.486 |
| 16 | 461 | <0.001 | 0.852 | 0.355 | <0.001 | 0.615 | 0.433 |
| 17 | 612 | 0.065 | 2.696 | 0.100 | 0.095 | 2.204 | 0.138 |
