# Supplementary material for: Clines on the seashore: The genomic architecture underlying rapid divergence in the face of gene flow
Source: Evol Lett. 2018 Aug 7;2(4):297–309. doi: 10.1002/evl3.74 (PMC6121805; doi:10.1002/evl3.74)
Supplement: Supplementary file 20 — Table S11: Numbers and proportions of clinal SNPs that were considered neutral and non‐neutral. [file EVL3-2-297-s020.docx]

**Table S11**: Numbers and proportions of clinal SNPs that were considered neutral and non-neutral. Non-neutral SNPs represent those that showed higher var.ex values than simulated neutral SNPs. Results are shown for three different var.ex thresholds identified by simulations. 35.69 was the threshold based on simulations with the set of parameters that seems most realistic given existing knowledge about the study system, and was used for the analyses shown in the main text; 19.26 and 47.48 represent the highest and lowest var.ex thresholds identified under a range of parameter values.

| **var.ex** | **19.26** | ***35.69*** | **47.48** |
| --- | --- | --- | --- |
| neutral | 68,500 (90.65%) | *73,671 (97.50%)* | 74,538 (98.64%) |
| non-neutral | 7,062 (9.35%) | *1,891 (2.50%)* | 1,024 (1.36%) |
