# Supplementary material for: Clines on the seashore: The genomic architecture underlying rapid divergence in the face of gene flow
Source: Evol Lett. 2018 Aug 7;2(4):297–309. doi: 10.1002/evl3.74 (PMC6121805; doi:10.1002/evl3.74)
Supplement: Supplementary file 21 — Fig. S1: Fitted clines for transformed centroid size (adjusted to the mean shore height) for females (black) and males (green). Fig. S2: Fitted cline for transformed shape (adjusted to a scaled size of 0.5) for females (black) and males (green). Fig. S3: Number of SNP pairs in which both SNPs are non‐neutral, divided by the number of SNP pairs containing at least one non‐neutral SNP. Fig. S4: Variation in the proportion of non‐neutral SNPs among map positions. Fig. S5: Genotypes at all non‐neutral SNPs placed on the genetic map. Fig. S6: Relationship between FST and cline slope. Fig. S7: Manhattan plots for (A) banded pattern on the shell, (B) beige colour, and (C) black colour of the shell. Fig. S8: Normalised contribution of each linkage group to shell size (A) and shape (B) variation using HEIDI. Fig. S9: Histograms of cline parameters in neutral and non‐neutral SNPs, based on the 19.26 var.ex threshold (A) and the 47.48 var.ex threshold (B). Fig. S10: Proportion of SNPs that were non‐neutral in each of the 17 LGs (LGs in order along the x‐axis). Fig. S11: Variation in the proportion of non‐neutral SNPs among map positions based on the 19.26 var.ex threshold (A) and the 47.48 var.ex threshold (B). Fig. S12: Distribution of cline slopes and centres of non‐neutral SNPs along the shore, based on the 19.26 var.ex threshold (A) and the 47.48 var.ex threshold (B). Fig. S13: Comparison between cline analysis and BayeScan outlier analysis. Fig. S14: Filtering of variant datasets after SNP calling. [file EVL3-2-297-s021.docx]

**Supplementary Information Appendix**

Clines on the seashore: The genomic architecture underlying rapid divergence in the face of gene flow

**Anja M. Westram^1,2,3^, Marina Rafajlović^4,5^, Pragya Chaube^1^, Rui Faria^1^, Tomas Larsson^6^, Marina Panova^7^,**

**Mark Ravinet^8^, Anders Blomberg^9^, Bernhard Mehlig^5^, Kerstin Johannesson^7^, and Roger Butlin^1,7^**

^1^Department of Animal and Plant Sciences, University of Sheffield, Sheffield, UK

^2^Current address: IST Austria, Am Campus 1, 3400 Klosterneuburg, Austria

^3^E-mail: anja.westram@ist.ac.at

^4^Current address: Department of Marine Sciences, University of Gothenburg, 40530 Gothenburg, Sweden

^5^Department of Physics, University of Gothenburg, 41296 Gothenburg, Sweden

^6^Department of Marine Sciences, University of Gothenburg, 40530 Gothenburg, Sweden

^7^Department of Marine Sciences - Tjärnö, University of Gothenburg, 45296 Strömstad, Sweden

^8^CEES (Centre for Ecological and Evolutionary Synthesis), University of Oslo, Oslo 0316, Norway

^9^Department of Chemistry and Molecular Biology, University of Gothenburg, 40530 Gothenburg, Sweden

Key words: hybrid zones, clines, local adaptation, speciation, inversions, molluscs

# SI Appendix, Methods

**Methods S1: Phenotypic cline fitting**

In preparation for phenotypic cline fitting, quantitative phenotypes (size and shape) were re-scaled to range between 0 and 1, for ease of comparison among traits and with SNP clines, such that the most extreme crab ecotype individual had a score of 0 and the most extreme wave ecotype individual had a score of 1. The expected phenotype for individual *i* was given by:

$$z_{i}= z_{crab}+ \left( z_{wave}- z_{crab} \right)f_{d_{i}}$$

where f_di_ is the expected frequency of the allele that has higher frequency in the wave habitat than in the crab habitat, at the individual’s position *d_i_*, which is obtained from the no-tail cline formula of Derryberry et al. (2014)

$$f_{d_{i}}=1/(1+e^{\frac{4\left( d_{i}-c \right)}{w}})$$

where *c* is the cline centre and *w* is the cline width.

The variance in phenotype, *v*, was allowed to change along the transect such that the variance at position *d_i_* was given by:

$v_{d_{i}}= v_{crab}+ {4v}_{hybrid}f_{d_{i}}\left( 1-f_{d_{i}} \right)+ {(v}_{wave}-v_{crab})f_{d_{i}}^{2}$

This is equivalent to the unimodal model of Gay et al. (2008). There was no evidence of bimodality for the traits analysed here.

Models were tested in which all variances were constrained to be equal, variances for the two ecotypes were equal but an elevation in the cline centre was possible (*v_crab_ = v_wave_ ≠ v_hybrid_*), variances for the two ecotypes were unequal but there was no elevation in the cline centre (*v_di_ = v_crab_(1 - f_di_) + v_wave_f_di_*), or where all three variances were independent.

The likelihood of an individual’s phenotype was obtained with the dnorm function in R, given the expected phenotype and variance from the cline model. The log-likelihood of the data was computed as the sum of log-likelihoods across individuals. Clines were fitted using the mle2 function in the R package bbmle (Bolker 2012) with the default method (‘Nelder-Mead’). Estimated model parameters are given in Table S4 and Table S5. Akaike’s Information Criterion was used to compare model fits.

The basic cline model was extended to allow for differences between the sexes or effects of covariates that are a function of cline position:

$$z_{i}= z_{crab}+ \left( z_{wave}- z_{crab} \right)f_{d_{i}}+b_{1.crab}x_{1}+\left( b_{1.wave}-b_{1.crab} \right)x_{1}f_{d_{i}}+b_{2.crab}x_{2}+\left( b_{2.wave}-b_{2.crab} \right)x_{2}f_{d_{i}}\ldots$$

where *x_j_* is a covariate and *b_j.crab_* and *b_j.wave_* are regression coefficients at the ends of the clines. For size, we tested the effects of sex (coded 0 for male, and 1 for female) and shore level at the snail position and for shape we tested the effects of sex, size and shore level.

Colour phenotypes were coded as present or absent for each class. We attempted to fit clines to the frequencies of classes with overall frequency in the sample >0.05 (Beige, Dark beige, Black, Banded, or Other). For these cline fits, end frequencies were expressed on a logit scale, to avoid convergence problems, or set to zero or one where appropriate.

**Methods S2: Reference genome assembly**

DNA from a single male Crab ecotype individual was extracted and used to generate a reference genome assembly. A combination of several Illumina sequencing datasets with different insert sizes ranging from ≈ 110 bp to ≈ 5,900 bp was used for contig assembly and initial scaffolding. These initial scaffolds were further gap-filled and extended using PacBio single molecule long reads. All datasets were generated from the same Crab ecotype individual.

Raw data were quality-checked using the software FastQC (<http://www.bioinformatics.ba-braham.ac.uk/projects/fastqc>). Initial quality results were summarized with multiqc, version 0.7 (Ewels et al., 2016). Libraries are summarized in Table S1.

For samples where the initial quality check indicated problems with PCR duplicates, a duplication removal step was run using the perl script “filterPCRdupl_v1.01.pl” (<https://git-hub.com/linneas/condetri>). After identification of possible adaptors and PCR primer contaminants for each dataset, these sequences were removed using cutadapt (Martin 2011) as run from within “Trim Galore!” (<https://www.bioinformatics.babraham.ac.uk/projects/trim_galore/>) or using Trimmomatic (Bolger *et al.* 2014), see Table S1 for details. After PCR duplication removal, adaptor and quality trimming of data and conversion of quality scores (Ewels *et al.* 2016) for all datasets to the standard Sanger encoding, FastQC was run to control and visualize the effect of trimming. A post trimming preqc (Simpson 2014) analysis was run to further evaluate the results of cleaning and trimming steps. The results of the above analyses were used to guide the selection of kmer size for contig assembly. Based on the estimated ideal kmer size from preqc and kmergenie, an initial test assembly was run using SOAPdenovo (Luo *et al.* 2012). Insert sizes for each library for this assembly was set according to preqc and/or the insert sizes aimed for during library preparation. The result from the initial assembly was used to optimize later assemblies with regard to insert size of each library.

A final set of assembled Illumina contigs was chosen based on comparison of the amount of raw data included in the assembly, contig lengths and NG50 value. This assembly contained a total of 2,776,845 sequences with a length of 200bp or longer.

Due to the high rate of heterozygosity expected in the data, several attempts to remove redundant contigs were made. A final approach using the software redundans (Pryszcz & Gabaldón 2016) was used with an identity setting of 95% and overlap set to 75%. After redundancy removal a total of 1,876,613 contigs was kept and further scaffolded and gap-filled using the scaffolder of SOAPdenovo.

The scaffolded Illumina assembly was used as input for further scaffolding using the PBJelly pipeline (English *et al.* 2012) together with long PacBio reads. In order to reduce computation time, only PacBio sequences above 500bp in length were used. The final scaffolded assembly contained a total of 388,619 scaffolds and contigs. Of those, 116,262 were 1,000bp or longer and used for further analysis.

The statistics of the final scaffolded assembly are summarized in Table S2. NG50 was calculated using the estimated genome size 1.35Gb (<http://www.genomesize.com>, (Vitturi *et al.* 1995)). The BUSCO (Simão *et al.* 2015) pipeline was used to assess completeness using both the eukaryotic and metazoan reference sets (summarized in Table S3).

**Methods S3: Generation of a linkage map**

In order to produce a linkage map, we generated a full-sib family by crossing a male and a virgin female Crab ecotype individual from the transect end in the lab. Offspring were raised in a seawater aquarium until large enough for DNA extraction. 183 offspring were chosen randomly (sexing was impossible due to immaturity), DNA was extracted from offspring and parents, and sequencing was performed as described above for the hybrid zone samples. The sequencing data were processed as described above up to the SNP calling step.

In order to avoid incorrect inferences in the map caused by genotyping errors, we aimed to include only high-quality genotypes and variants, and applied stringent filtering as shown in Fig. S14. Of 74,424 markers passing these filters, 1,112 had missing genotypes for either one or both the parents. For SNPs with a single parent missing a genotype, we inferred the missing parental genotype using the segregation pattern observed in the offspring. SNPs where both parents lacked a genotype were discarded. The remaining 74,027 SNPs were used as the initial input for the Lep-Map2 package (Rastas *et al.* 2013, 2016). The Lep-Map2 “Filtering” module was used to remove SNPs that showed significant segregation distortion (“dataTolerance” set to a p-value of 0.01) or missing data in 92 or more of the 183 offspring (“missingLimit” set to 92). The remaining 61,161 SNPs were grouped into LGs with the “SeparateChromosomes” module, using a LOD score limit of 12 and a minimum LG size of 20 SNPs. Markers that were not assigned to LGs in this step were added, where possible, using the “JoinSingles” module with the LOD threshold set to 3. Contigs that contained SNPs on multiple LGs were discarded, as these likely represent either mapping or assembly errors.

The order of SNPs on each LG was estimated using the “OrderMarkers” module to generate separate maps based on recombination in the male and female parents. A recombination rate parameter of 0.005 and the Kosambi function were used in this step and the task was repeated until the likelihood stabilised. The order with the highest likelihood was chosen for each LG and manually curated to remove single markers from the ends of LGs where they were more than 5cM from their nearest neighbour or groups of up to 3 markers at the same map position that were more than 10cM from their nearest neighbour. Optimization runs with “OrderMarkers” were then repeated until the likelihood no longer increased and the highest likelihood orders were used in the next step.

After creating parent-specific maps with all SNPs that passed filters, we removed SNPs for which LepMap2 estimated genotyping error ≥0.1 during marker ordering. We found that the merging function in LepMap2 was unable to produce a satisfactory combined map from our two parent-specific maps because of the way it treated multiple singly-informative SNPs between pairs of bi-parentally informative SNPs. Therefore, we reduced our mapping data set in the following way, using a custom R script:

1. We retained all bi-parentally informative SNPs
2. For contigs that lacked bi-parental SNPs but contained both male-informative and female-informative SNPs, we created a synthetic bi-parental SNPs for each independent pair of adjacent male- and female-informative SNPs that were <1kb apart.
3. We removed all single-parent SNPs except in contigs that contained no bi-parental or synthetic bi-parental SNP where we retained just one SNP, selecting the one with the least missing values.
4. We created two data sets, one with bi-parental and male-informative SNPs and one with bi-parental and female-informative SNPs.

We ran the LepMap2 “OrderMarker” module again with these reduced data sets, as detailed above, generating two parent-specific maps. Markers isolated at the ends of LGs were removed, as above. We also removed any single marker with map position >5 cM from a group of other markers on the same contig that shared one map position. These maps were then combined using a custom R script by averaging the positions of bi-parentally informative SNPs and then interpolating the positions of single-parent SNPs on the basis of their distances to the nearest bi-parental SNPs in the relevant single-parent map. This process avoided the use of map positions assigned to male-informative SNPs in the female map, and vice-versa, which created the problems with the standard procedure in LepMap2. This approach resulted in 18,942 markers overall in the sex-averaged maps.

**Methods S4: DNA extraction, capture sequencing and bioinformatics**

For 373 mature individuals from across the contact zone, DNA for capture sequencing was extracted from a small piece of foot tissue using a CTAB protocol (Panova *et al.* 2016).

The target capture technology allows for the sequencing of known genomic regions, which are targeted with probes and enriched before sequencing. With probes of 120bp, this approach produces data for genomic regions of a few hundred base pairs each, usually including multiple SNPs in our study species. We used a total of 40,000 probes. The majority of these, that is, 38,684 probes, were newly designed probes randomly distributed across the genome, with only a single probe in a given genomic region (this was ensured by using only probes mapping to different contigs of a previous version of the *L. saxatilis* genome assembly). In addition, we used 1,316 probes designed for earlier work (Westram *et al.* 2016) where multiple probes targeted the same genomic region. Of these, 842 targeted outlier loci or loci showing expression differences in earlier studies (Galindo *et al.* 2010; Westram *et al.* 2014, 2016; Ravinet *et al.* 2016; Panova et al. unpublished) and 474 targeted random loci across the genome. All probes were designed by first generating a larger set of potential probes and then filtering those according to GC content and number of mapping locations in the reference genome (probes with more than two mapping locations indicate repetitive regions and were excluded). Probe design was performed by RapidGenomics (Gainesville FL, United States).

Library preparation, capture and paired-end sequencing for all individuals was performed by RapidGenomics. For some individuals, both paired-end and single-end libraries were sequenced; the data were pooled in the bioinformatic pipeline (see below).

All fastq files obtained from capture sequencing for the hybrid zone individuals (see above) and the mapping family individuals (see below) were trimmed with Trimmomatic (v. 0.36, (Bolger *et al.* 2014)), retaining reads with a minimum length of 70bp after filtering. Reads were then mapped to the *L. saxatilis* reference genome (assembly described in Methods S2) using bwa-mem (Li 2013). Paired-end reads were mapped separately from single-end reads.

Capture sequencing datasets often contain considerable amounts of data from non-targeted regions, which may have low coverage depth and are not useful for downstream analyses. We aimed to filter out such regions early in the pipeline in order to speed up the following steps. To do so, we first counted the number of reads mapping to each genome contig in each paired-end mapping file. Only reads mapping to contigs with at least 10 reads in at least 150 individuals were retained for downstream analyses.

Bam files were filtered by removing secondary hits and hits with a mapping quality less than 20. Potential PCR and optical duplicates were removed using PicardTools MarkDuplicates (http://broadinstitute.github.io/picard/), and overlap between paired-end reads was soft-clipped using bamUtil clipOverlap (https://github.com/statgen/bamUtil). Single- and paired-end read bam files from the same individual were then merged into a single file in samtools (v. 1.3.1, (Li *et al.* 2009)). After that, we used samtools to call SNPs, including only bases with a quality of at least 20.

All downstream analyses were performed on either the resulting SNP calls or the allelic read depths obtained along with the SNP calls. Further filtering was necessary depending on the requirements of each analysis; filtering steps are described in Fig. S14 and were performed using vcftools (Danecek *et al.* 2011) or custom R scripts. In particular, we filtered out any SNPs that were more than 1,000bp away from any SNP included in the linkage map. The rationale for this was that we aimed to ensure that only genomic regions showing normal segregation patterns were used. We deemed this filtering necessary in order to filter out duplicated loci, which are expected to deviate from normal segregation patterns. The presence of such loci in the *L. saxatilis* genome had been suggested by previous analyses. These loci are not usable for our cline and association analyses as reads from multiple loci may map to the same position in the genome.

**Methods S5: Fitting of SNP clines**

After removing SNPs with a minor allele frequency smaller than 10% from the filtered hybrid zone SNP set, we used custom R scripts to filter loci further, as follows:

1. SNPs with significantly more heterozygotes than expected under Hardy-Weinberg equilibrium across the whole sample were excluded (Chi-square test, critical P = 0.01). These loci are putatively influenced by confounding of reads from paralogous loci. Because loci with clinal variation in frequency are expected to show an overall deficiency of heterozygotes, this test is lenient.
2. SNPs with significantly different frequencies of heterozygotes in males and females were excluded (Chi-square test, critical P = 0.01). Our data suggest that there is no heteromorphic sex chromosome pair (as was expected based on (Rolan-Alvarez *et al.* 1996)) because there is no set of loci for which heterozygotes are absent in one sex. However, some loci were removed because they showed an excess of heterozygotes in females and a deficiency in males, perhaps suggesting linkage to a sex-determining region.
3. SNPs with a higher frequency of either the reference or alternative allele near the centre of the transect than at either end were excluded by dividing the transect into three equal parts and removing loci where the allele frequency in the centre was more than 0.2 greater than at either end.

For these steps, a simple classification into genotypes was used: genotype RR (reference homozygote) if the read count for the A allele was <2, genotype AA (alternative homozygote) if the read count for the R allele was <2, genotype AR (heterozygote) if both read counts were >1.

Allele frequency change along the transect was then modelled as either fixed (parameter, *p_all_*) or clinal. Clinal models were only tested if an initial estimate of the allele frequency difference between the ends of the transect was >0.1. Clinal models followed the no tail, left tail, right tail and independent tail formulae used in (Derryberry *et al.* 2014) with adjustment for non-fixed end frequencies. Since the frequency of the allele at higher frequency at the Wave environment end of the transect was used in all cline fits, the frequency formula took the form:

$$p_{d}= p_{crab}+ p_{diff}f_{d}$$

where *p_d_* is the allele frequency at line distance *d, p_crab_* is the frequency in the Crab habitat, *p_diff_* is the allele frequency difference between the Crab and Wave habitats and *f_d_* is the admixture proportion given by the relevant cline formula.

Calling genotypes from read counts involves some uncertainty, especially where the total read count is low. Therefore, we used the approach of Buerkle and Gompert (2013) to find the likelihood of the read counts for the two alleles, for each individual, given the individual’s position and the relevant model for allele frequency change along the transect. Specifically, we summed the probabilities of the three possible genotypes given the read counts and a locus-specific error rate *e_l_*, weighted by the probabilities of the genotypes given the allele frequency *p_d_* (equations 3, 4; (Buerkle & Gompert 2013)). The genotype probabilities assumed Hardy-Weinberg proportions. The log-likelihood of the data for a SNP was then the sum of individual log-likelihoods. Models were fitted using the mle2 function in the R package bbmle (Bolker 2012) as for phenotype clines, except that method “L-BFGS-B” was used to allow for constraints on fitted parameters and the ‘parscale’ option was used to adjust step size for the search algorithm according to the initial values (see Table S7 for parameters with constraints and initial values).

In order to ensure that cline parameters were robust to the sample distribution on the shore, particularly near the habitat transition where a lot of individuals “clump” in the same place, we repeated the cline fitting procedure 10x for each SNP, with the spatial position of each individual slightly jittered each time. We then identified SNPs for which at least 8 of the 11 jittered replicates were clinal (either tailed or non-tailed). These SNPs were labelled as clinal, and the average cline slope and average cline centre across clinal jittered replicates was used in downstream analyses. Analogously we identified non-clinal SNPs as those where at least 8 jittered replicates were non-clinal (either because the estimated allele frequency difference between cline ends was smaller than 0.1 or because a model of no allele frequency change provided a better fit than a clinal model). Any SNPs that showed inconsistent patterns across jittered replicates are indicated as “not clearly associated with any category” in Table S6 and were not used in downstream analyses. A small number of clinal loci where the fitting process failed, and which consequently had var.ex values < 0 or F_ST_ values > 1, were excluded from further analyses (0.5% of all SNPs for which cline analysis was performed).

## **Methods S6: Estimation of dispersal parameters**

Dispersal was estimated from the relationship between the product of rates of allele frequency change (*s_i_s_j_*) and the linkage disequilibria (*D_ij_*) between pairs of loci (*i,j*) given by Barton and Gale (31), using an approach similar to Hollander et al. (32). We divided the central part of the transect into 6 blocks with boundaries at 70, 75, 80, 85, 90, 95, 100 and 110m from the Crab end. For each block, we calculated *D_ij_* using the genetics package in R (<https://CRAN.R-project.org/package=genetics>) and *s_i_s_j_* by taking the slopes of the fitted clines at the mean position of individuals within the block, for pairs of loci that met the following criteria:

1. They both had significant cline fits, with centres in the range 60 - 120m, widths in the range 2 - 40m, centre + width < 150m, centre – width > 1m and *p_diff_* > 0.2,
2. They were both polymorphic within the block,
3. They had the highest variance explained by the cline fit among SNPs in the same contig,
4. They were on different linkage groups (therefore, recombination rate, *r* = 0.5).

Within each block, we regressed *D_ij_* on 3*s_i_s_j_* with the intercept at zero. Given Barton and Gale’s formula, and assuming sampling after dispersal (giving the factor of 3 = (1 + *r*)/*r*), the regression coefficient is then an estimate of σ^2^, where σ is the standard deviation of parent-offspring distances in the transect dimension. We combined estimates across blocks by taking an average, weighted by the standard error of each estimate. We also repeated the analysis 50 times, shifting the block boundaries by a uniform random increment in the range (-1, 1) to avoid any bias due to a particular partition. We took the median across these runs as our best estimate of σ, and the 3^rd^ and 48^th^ ranked values as the range of plausible estimates.

## **Methods S7: Association and linkage disequilibrium analyses**

Due to statistical association between genotypes and phenotypes emerging in hybrid zones, it is possible to identify genomic regions underlying divergently selected traits (Rieseberg & Buerkle 2002; Lindtke *et al.* 2013; Nadeau *et al.* 2014; Hollander *et al.* 2015). Here, we used association analysis to investigate the genetic architecture of three divergent traits (scaled centroid size, shell shape, shell colour/banding pattern).

The input dataset was generated from the filtered hybrid zone SNP set (see above, “Bioinformatic analyses”, and Fig. S14). For the 106,599 SNPs passing filters, imputation of missing genotypes was performed using LinkImpute (Money *et al.* 2015) as the association analysis methods do not allow for missing data. To increase the speed, the imputation process was performed separately for each LG. If a SNP was not on the linkage map, it was assigned to an LG based on the physically closest SNP that was on the map, if possible.

The association analysis was performed with the full set of genotypes for each of the phenotypic traits: 1. scaled centroid size; 2. scaled RW1 for shape variability; 3. presence/absence of shell banding, black, beige and dark beige colours. We implemented the single-SNP association analysis from the GenABEL R package (Aulchenko *et al.* 2007). For scaled centroid size, we included sex as a covariate while for scaled RW1, size was used as a covariate. The analysis used the egscore() function which implements the EIGENSTRAT method (Price *et al.* 2006). It accounts for population stratification by regressing genotype and phenotype on the principal axes of variation obtained by decomposing the identity-by-state matrix of the individuals. We included the first four PC axes, based on examination of the scree plot (the number of axes had only minor effects on the results when numbers between 3 and 10 were tested). Multiple testing was accounted for by using the permutation method in the egscore function with the option, times = 1,000. The genome-wide threshold for p-values was chosen to be 0.05. The proportion of total phenotypic variance explained by each significant SNP was estimated by dividing the χ^2^_1_ test statistic by the number of samples (<http://forum.genabel.org/viewtopic.php?f=6&t=760&p=1449&hilit=proportion+variance+explained#p1449>). The genomic inflation factor (λ) was used to check for adequate correction for population structure.

For the continuous traits, we expected polygenic inheritance and limited power to detect single-locus associations. Therefore, the software HEIDI (Kostem & Eskin 2013) was used to estimate the overall heritability of size and shape (confidence intervals were obtained using a “leave one out” jackknife over individuals), and to partition heritability among chromosomes. For LGs 6, 14 and 17, we also partitioned the contributions of the nnBlock and the rest of the LG.

The data set used for association mapping was also used to calculate linkage disequilibrium between SNPs within nnBlocks and outside nnBlocks on the same linkage groups (LG6, LG14, LG17) using the genetics package in R (<https://CRAN.R-project.org/package=genetics>). We calculated mean LD (and its standard error), expressed as the unsigned correlation coefficient, between SNPs within contigs, between contigs at the same map position, between contigs at different map positions but within 5cM, and between more distant contigs. In each case, we did the calculation separately for crab ecotype and wave ecotype (defined as >20m from the mean position of non-neutral cline centres).

**Methods S8: Clustering**

We first tested whether the 17 LGs differed with regard to the proportion of non-neutral SNPs. We ran 1,000 permutations of SNPs among all map positions, and then tested whether the variance (weighted by the total number of SNPs per LG) in the proportion of non-neutral SNPs between LGs was higher in the observed data than in any of the permutated datasets, indicating differences between LGs.

We then asked whether regions within LGs show differences, possibly indicating clustering. We permuted contigs among LG positions (contigs rather than SNPs were permuted in order to maintain clustering at the within-contig level; see below), and then asked whether the observed variance between linkage map positions (weighted by the total number of SNPs per map position) was higher than in 95% of the permuted runs.

Finally, we asked about clustering at the base-pair scale within contigs, using contigs with at least three SNPs and at least two non-neutral SNPs. After obtaining all within-contig pairs of SNPs that contained at least one non-neutral SNP, we asked in what proportion of these pairs *both* SNPs are non-neutral. If non-neutral SNPs are clustered at the contig level, a decrease of this statistic with physical distance between SNPs is expected.

**Methods S9: BayeScan analysis**

To compare the set of non-neutral SNPs identified using cline analysis to outliers obtained with a genome scan, we ran BayeScan (Foll & Gaggiotti 2008) using 30 individuals from each cline end (mimicking typical sampling for a genome scan). Only SNPs included in both the BayeScan and the cline analysis are considered in the following (51,932 SNPs). BayeScan estimates locus-specific F_ST_ values (which are expected to be elevated for loci under divergent selection) as well as q-values reflecting the probability that a locus is under divergent selection, after correction for false discovery rate. We first ran BayeScan with standard settings (q-value of 0.05 and prior odds parameter of 1,000).

To account for differences in stringency between the two analyses, we also compared equally-sized sets of non-neutral SNPs from the two analyses. Specifically, we compared the 143 BayeScan outliers identified at a q-value of 0.05 with an equally-sized set of non-neutral SNPs from the cline analysis (generated by picking the top clinal loci after ranking them by their var.ex value).

Finally, we also used BayeScan with more lenient settings (prior odds parameter of 100) and compared all SNPs with a locus-specific F_ST_ > 0.1 with an equally-sized set of non-neutral loci from the cline analysis.

# SI Appendix, Figures


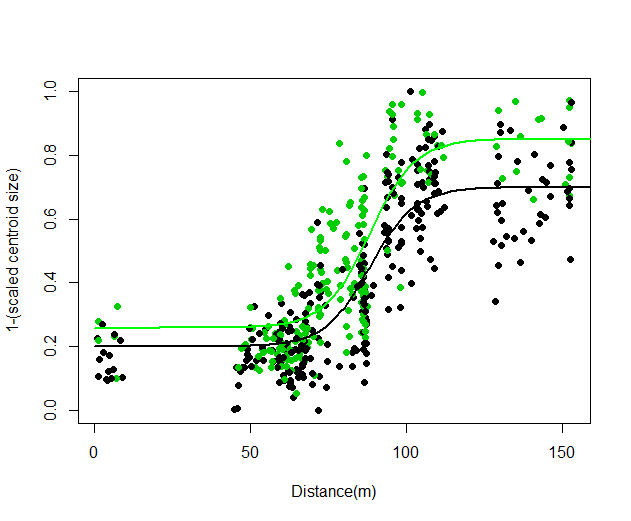


**Fig. S1**: Fitted clines for transformed centroid size (adjusted to the mean shore height) for females (black) and males (green). Back-transformed mean sizes were: female crab 16.27mm, male crab 14.71mm, female wave 6.91mm, male wave 5.34mm. Only mature individuals were included.


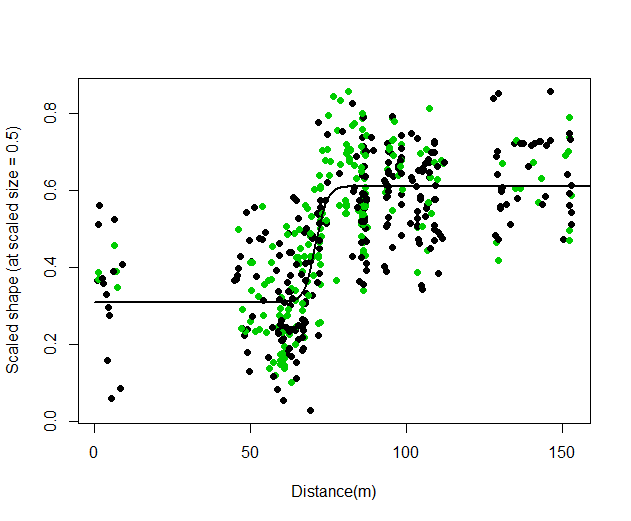


**Fig. S2**: Fitted cline for transformed shape (adjusted to a scaled size of 0.5) for females (black) and males (green). Only mature individuals were included.

A) B)


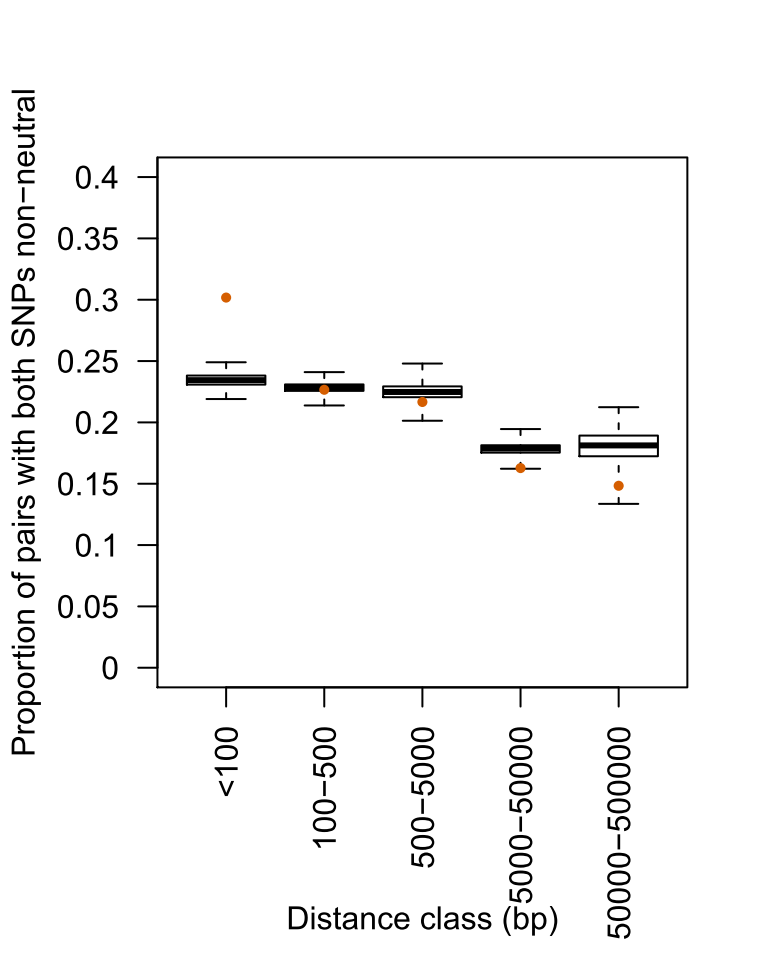

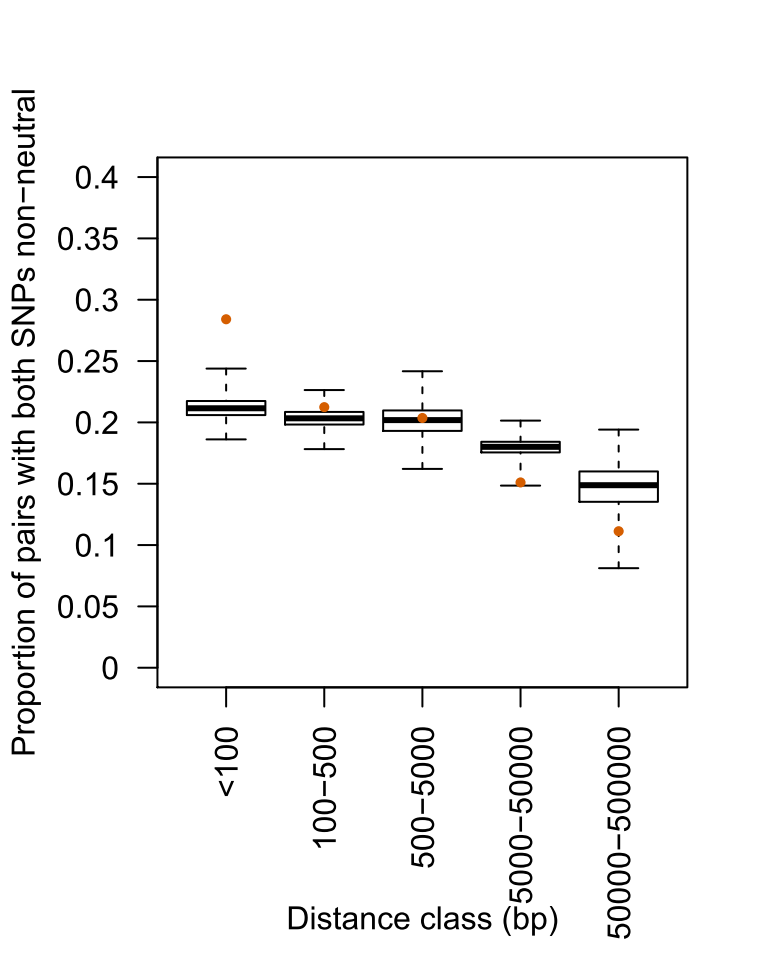


**Fig. S3**: Number of SNP pairs in which both SNPs are non-neutral, divided by the number of SNP pairs containing at least one non-neutral SNP. SNPs within a pair were always in the same contig and were grouped according to the distance between SNPs (x-axis). Orange points represent observed data; boxes represent results based on 1,000 permutations of SNPs within contigs. A) All SNPs, B) all SNPs excluding those in the three high-LD regions.


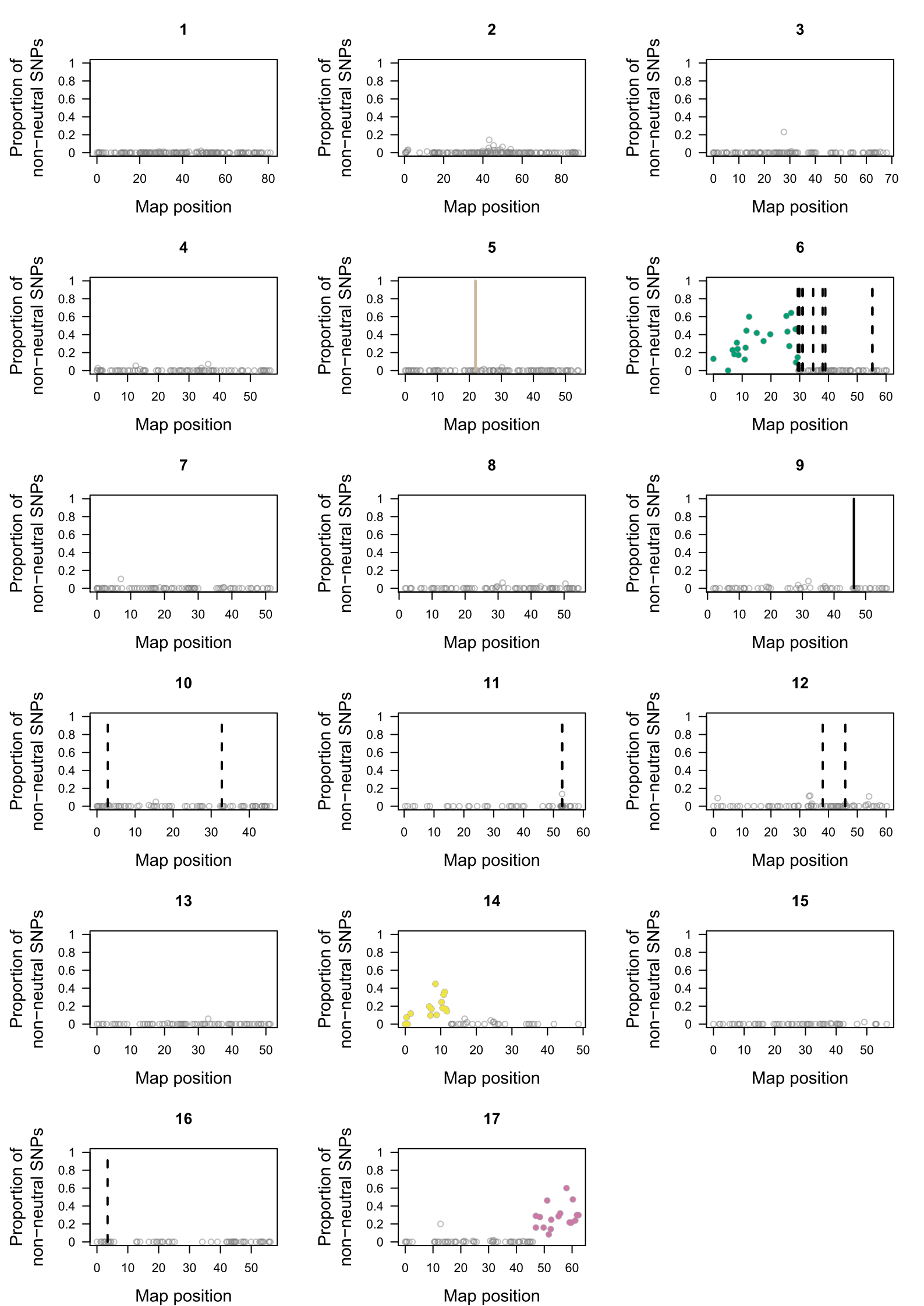


**Fig. S4**: Variation in the proportion of non-neutral SNPs among map positions. Each subplot represents one LG (title). Variation between map positions was significant for LGs 3, 6, 14 and 17. Variation between 10cM windows was significant for LGs 2, 4, 6, 9, 14 and 17. Three long genomic regions (“nnBlocks”) with elevated proportions of non-neutral SNPs, as well as elevated LD (see Fig. S5) are indicated in colour. Positions of SNPs significantly associated with three colour traits are indicated by vertical lines (black solid line = black shell colour; beige solid line = beige shell colour; black dashed line = banding pattern).


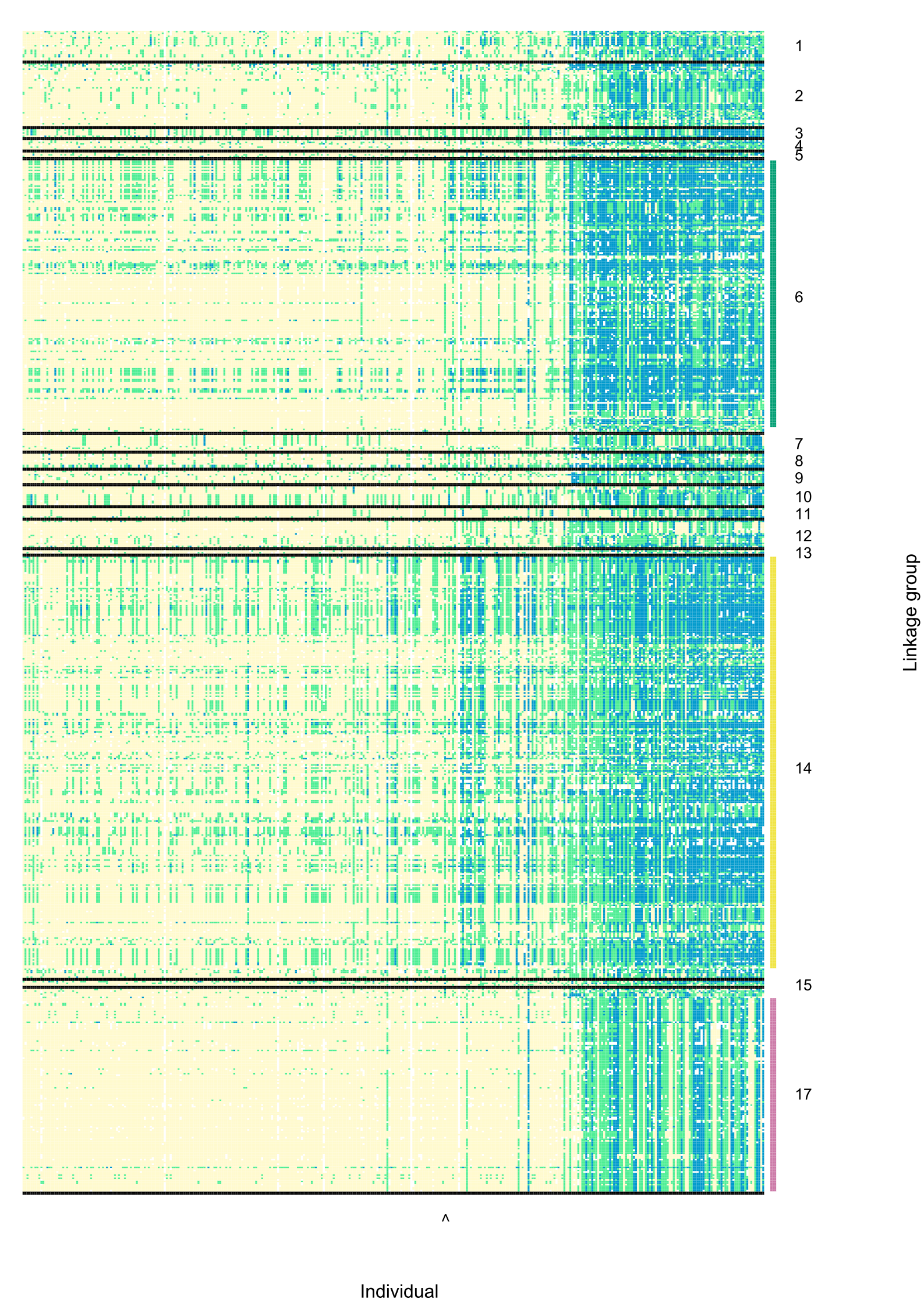


**Fig. S5**: Genotypes at all non-neutral SNPs placed on the genetic map. The x-axis represents individuals sorted according to their position on the path along the shore (left: Crab end; right: Wave end. The habitat transition from rock platform to cliff (arrow 2 in Fig. 1) is indicated with an arrow. On the y-axis, SNPs are sorted by linkage groups, and by map position within linkage groups (the “top” map position within each LG is analogous to the leftmost position in the respective subplot in Fig. S4). Yellow and blue colours represent the two homozygous genotypes (blue = the allele that increases in frequency in the Wave ecotype); green represents the heterozygote, and white indicates missing data. The position of the three nnBlocks is indicated to the right of the plot. Note the wide extent of linkage disequilibrium in these regions (indicated by long vertical bars of consistent colour), compared to other genomic regions.

A)
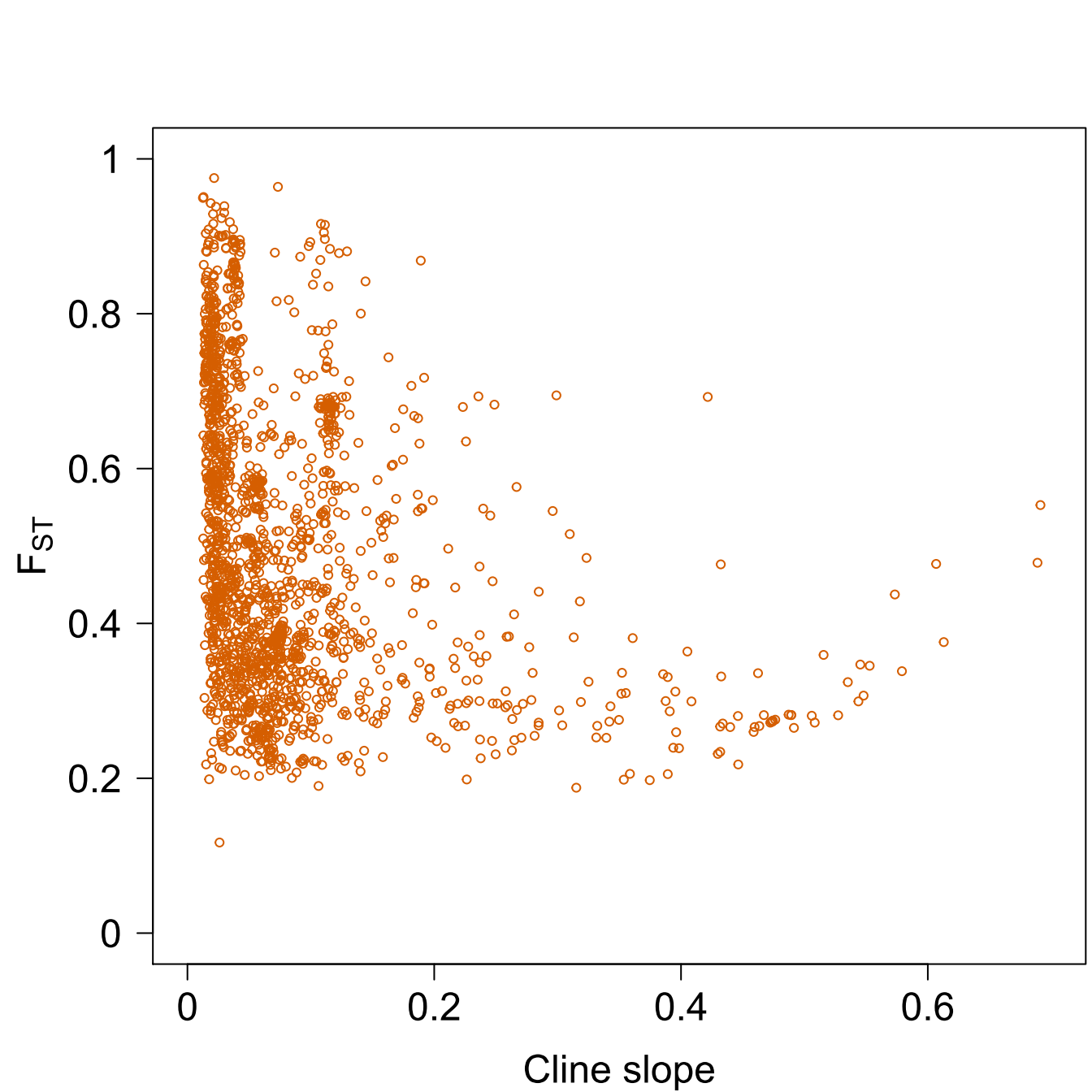
B)
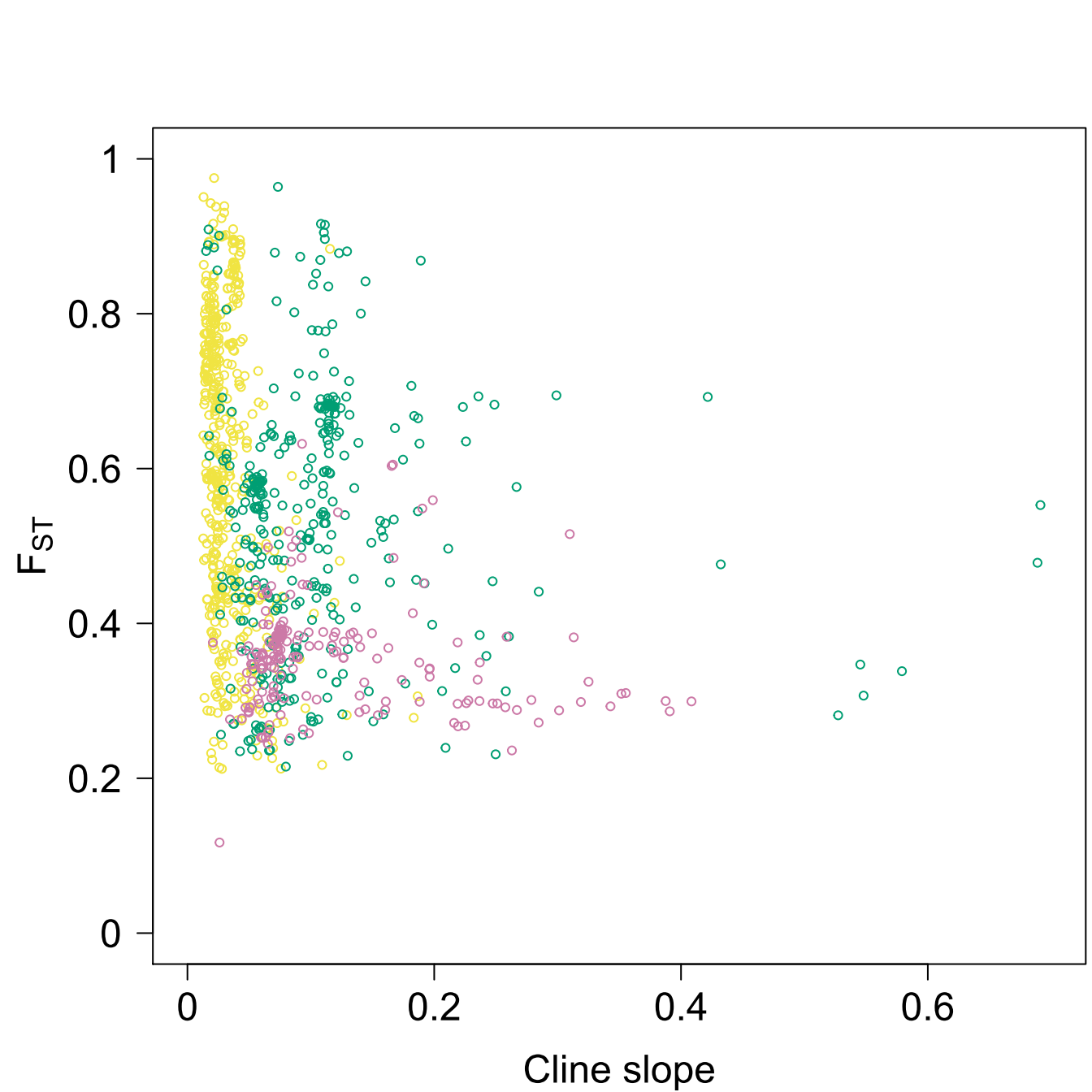


**Fig. S6**: Relationship between F_ST_ and cline slope. F_ST_ estimates are based on allele frequencies estimated for cline ends during the cline fitting process. A) and B) Results of cline analysis; identification of non-neutral SNPs based on simulations of clines under a model of primary divergence. A) All non-neutral SNP (orange). B) Non-neutral SNP in nnBlocks on LG6 (green), LG14 (yellow), and LG17 (purple).

A)


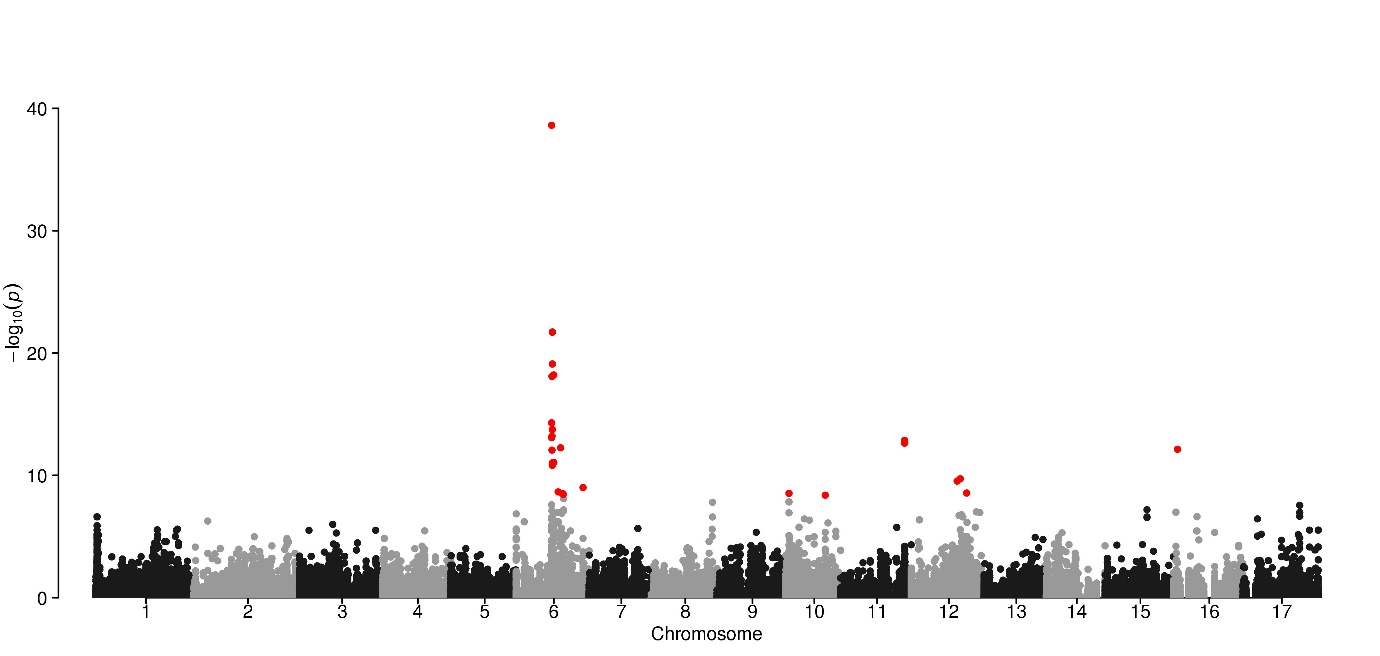


B)


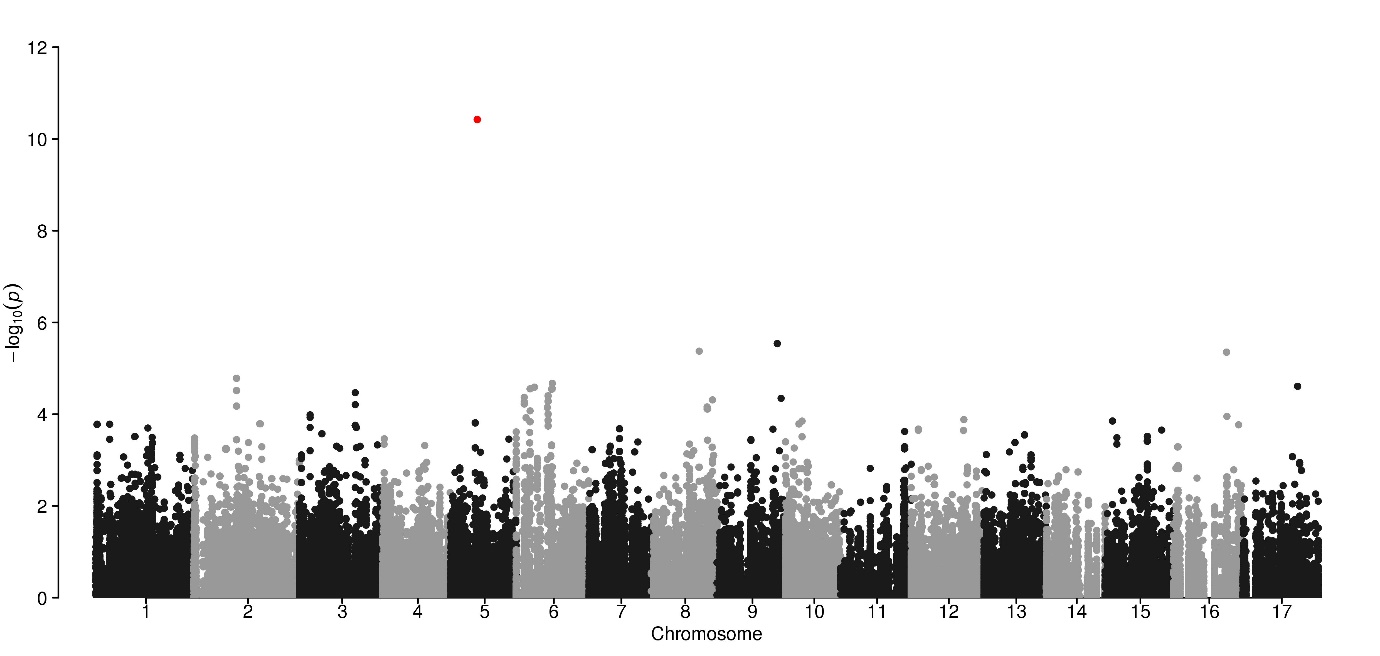


C)


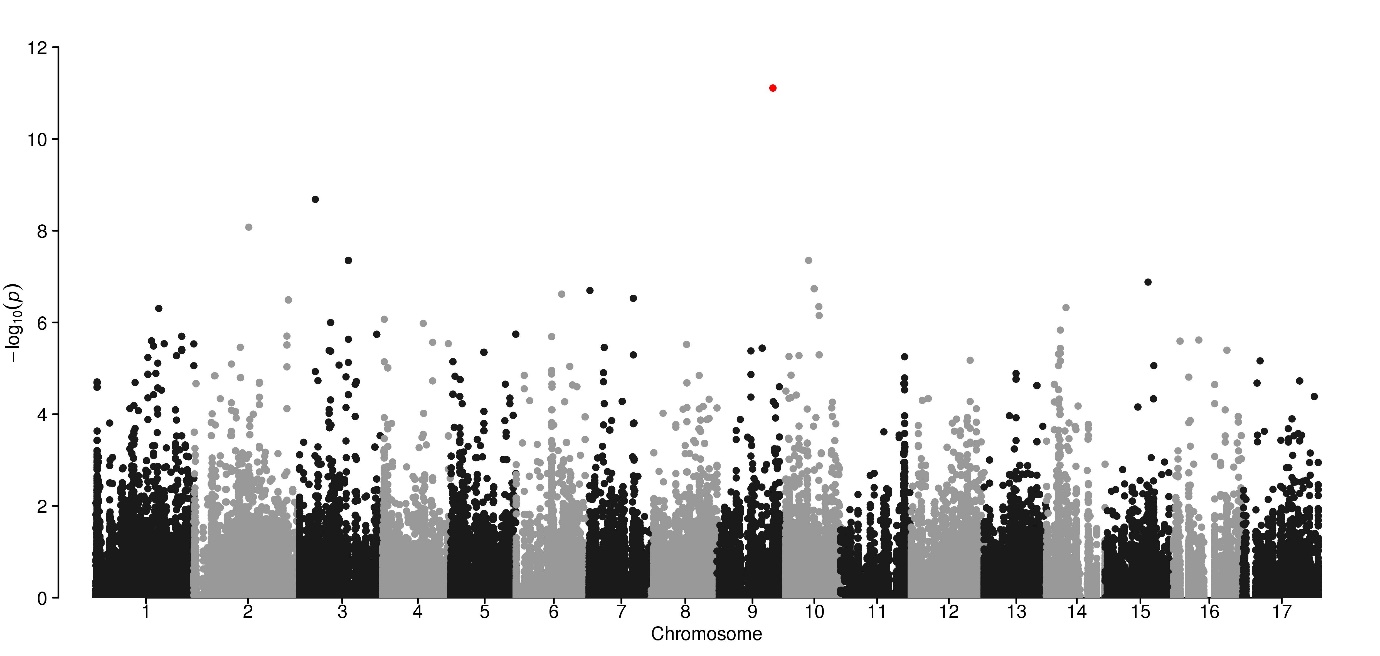


**Fig. S7**: Manhattan plots for (A) banded pattern on the shell (B) beige colour and (C) black colour of the shell. The SNPs with genome-wide threshold p-value < 0.05 obtained after permutations tests are highlighted in red.

A)


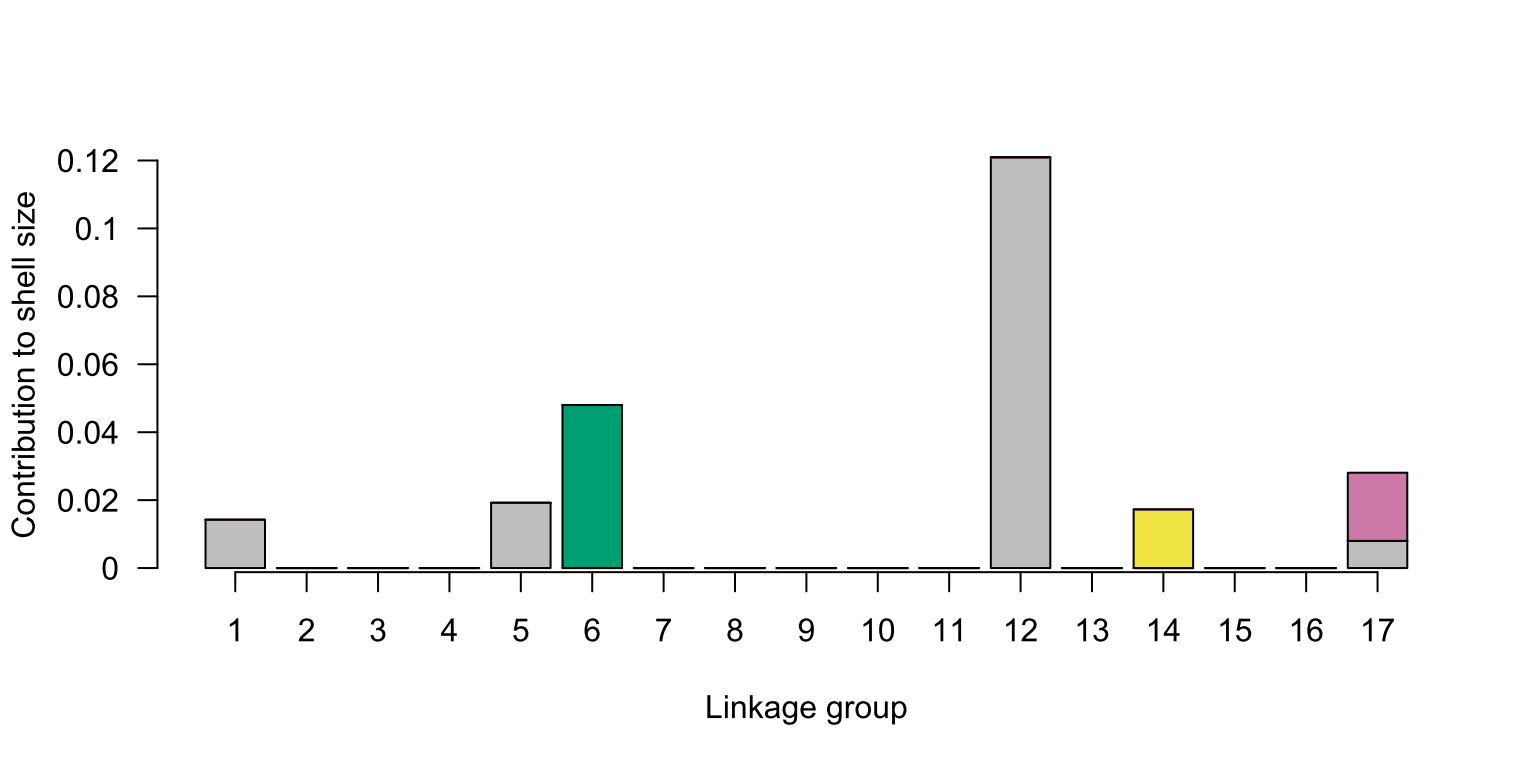


B)


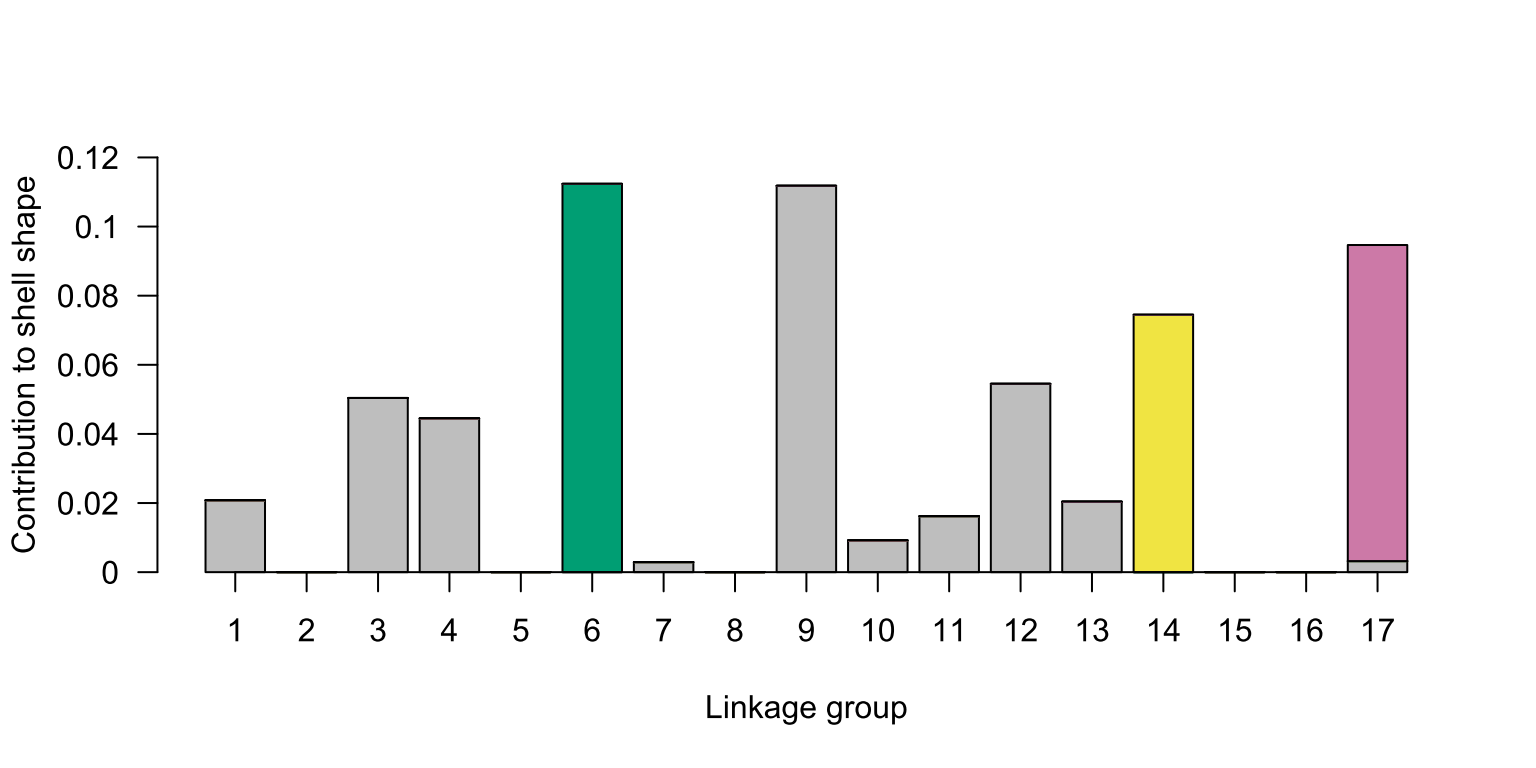


**Fig. S8**: Normalised contribution of each linkage group to shell size (A) and shape (B) variation using HEIDI. For linkage groups with nnBlocks (6, 14 and 17), contributions are split into the nnBlocks (colour) and the rest of the linkage group (grey).

A)


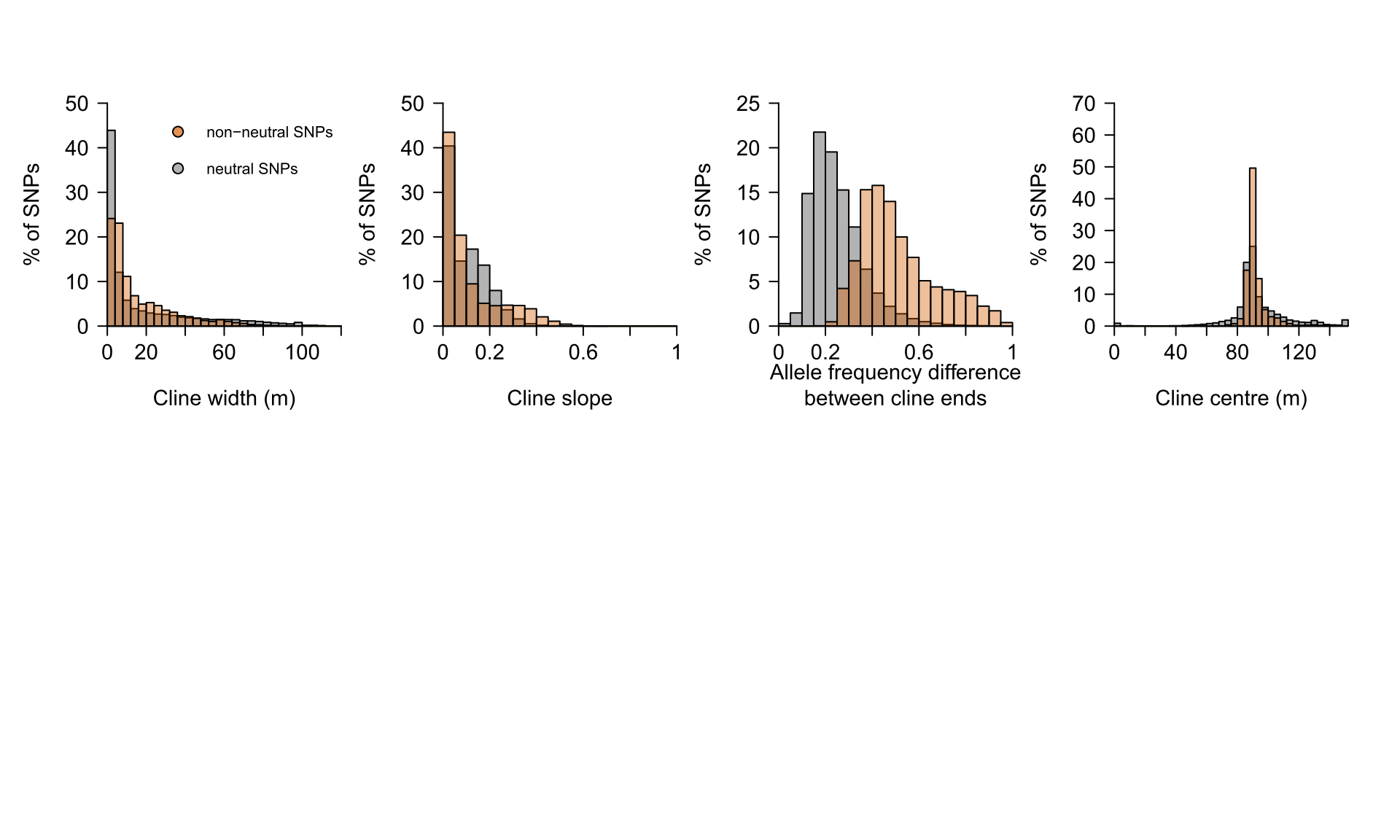


B)


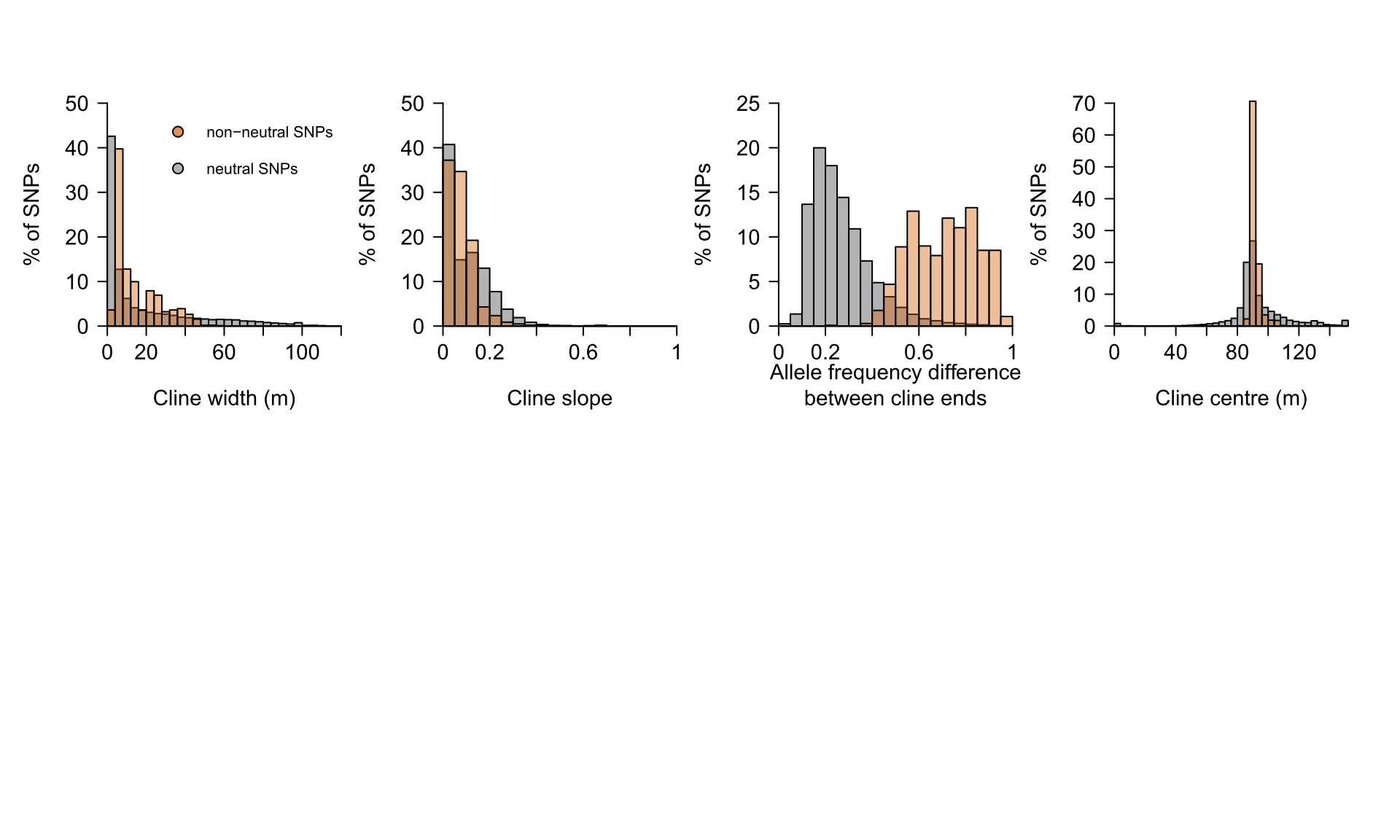


**Fig. S9**: Histograms of cline parameters in neutral and non-neutral SNPs, based on the 19.26 var.ex threshold (A) and the 47.48 var.ex threshold (B).

A)


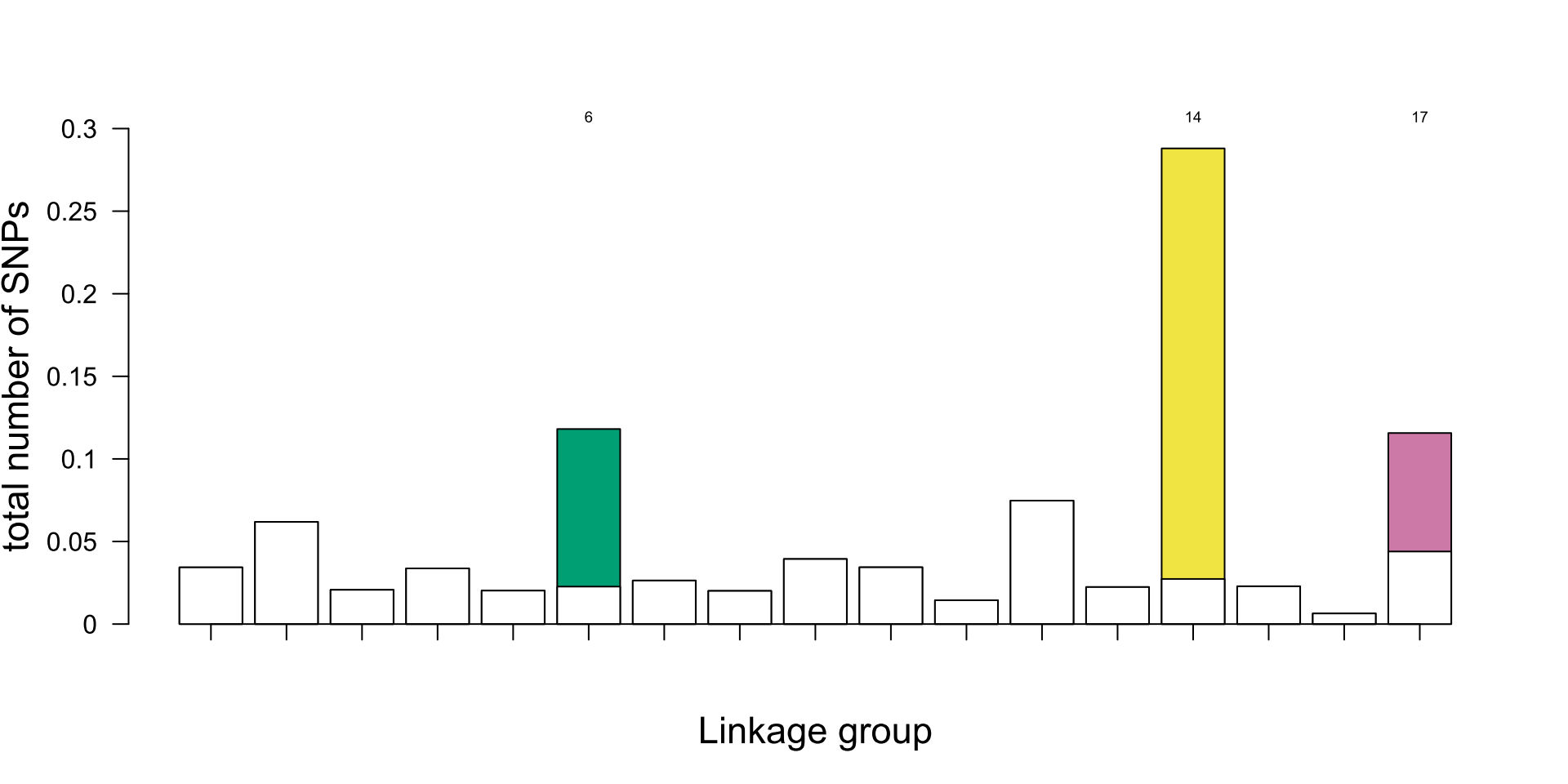


B)


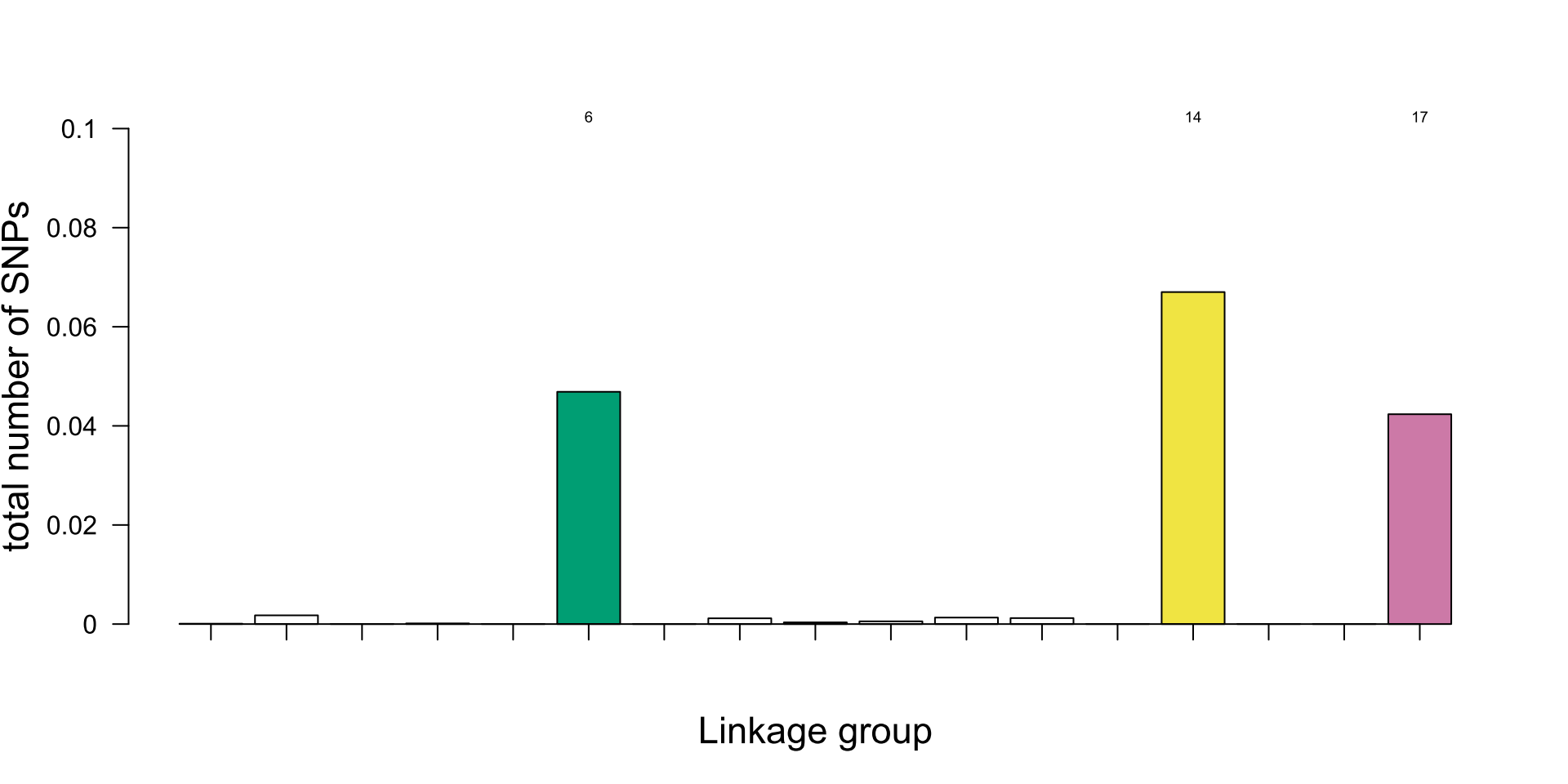


**Fig. S10**: Proportion of SNPs that were non-neutral in each of the 17 LGs (LGs in order along the x-axis). SNPs within three large regions of high linkage disequilibrium (“nnBlocks”) are shown in colour. A) var.ex threshold of 19.26; B) var.ex threshold of 47.48.

A)


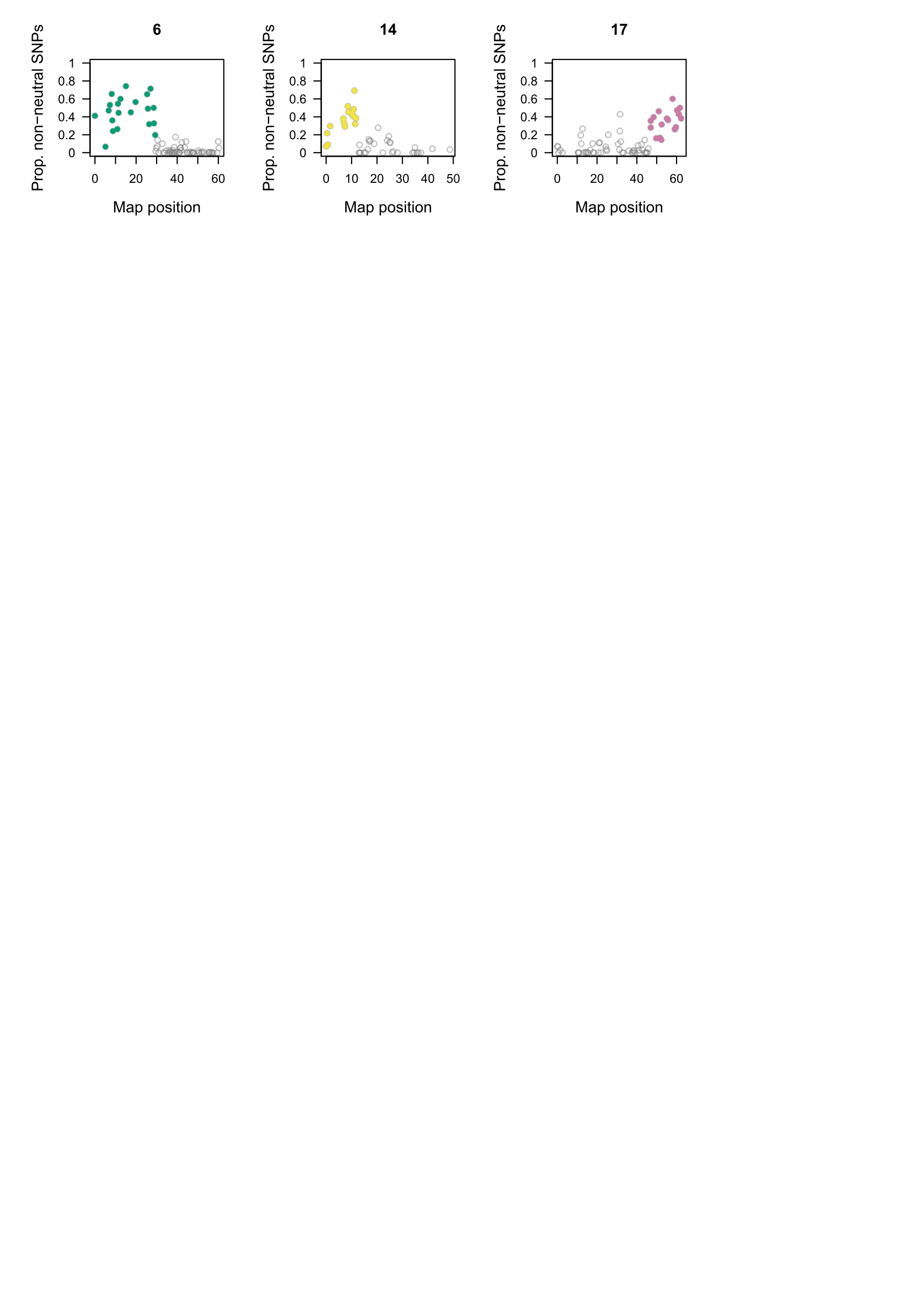
B)


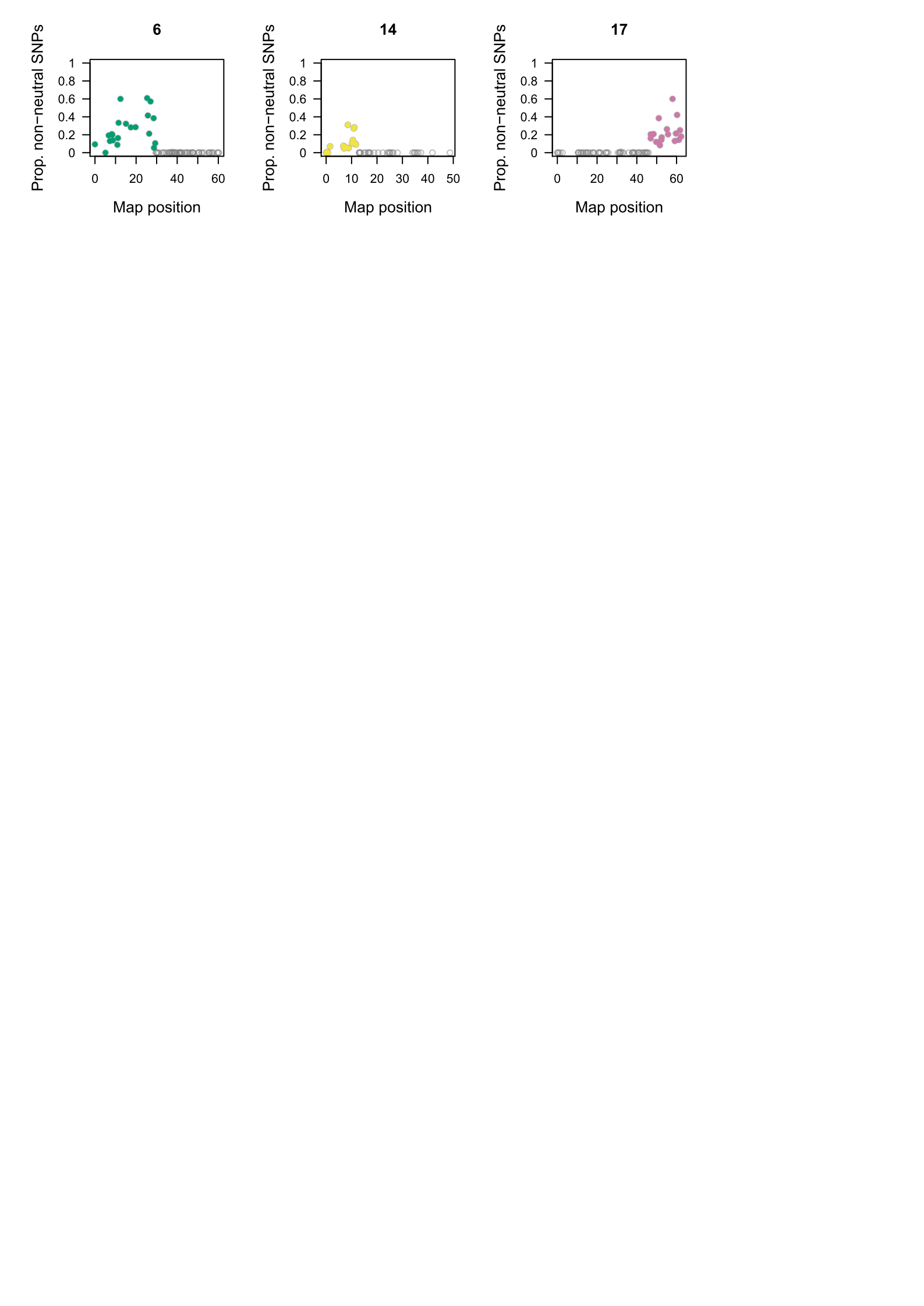


**Fig. S11**: Variation in the proportion of non-neutral SNPs among map positions. Each subplot represents one LG (title; only LGs with large numbers of non-neutral SNPs shown). Three long genomic regions (“nnBlocks”) with elevated proportions of non-neutral SNPs, as well as elevated LD are indicated in colour. A) var.ex threshold of 19.26. B) var.ex threshold of 47.48.

A)


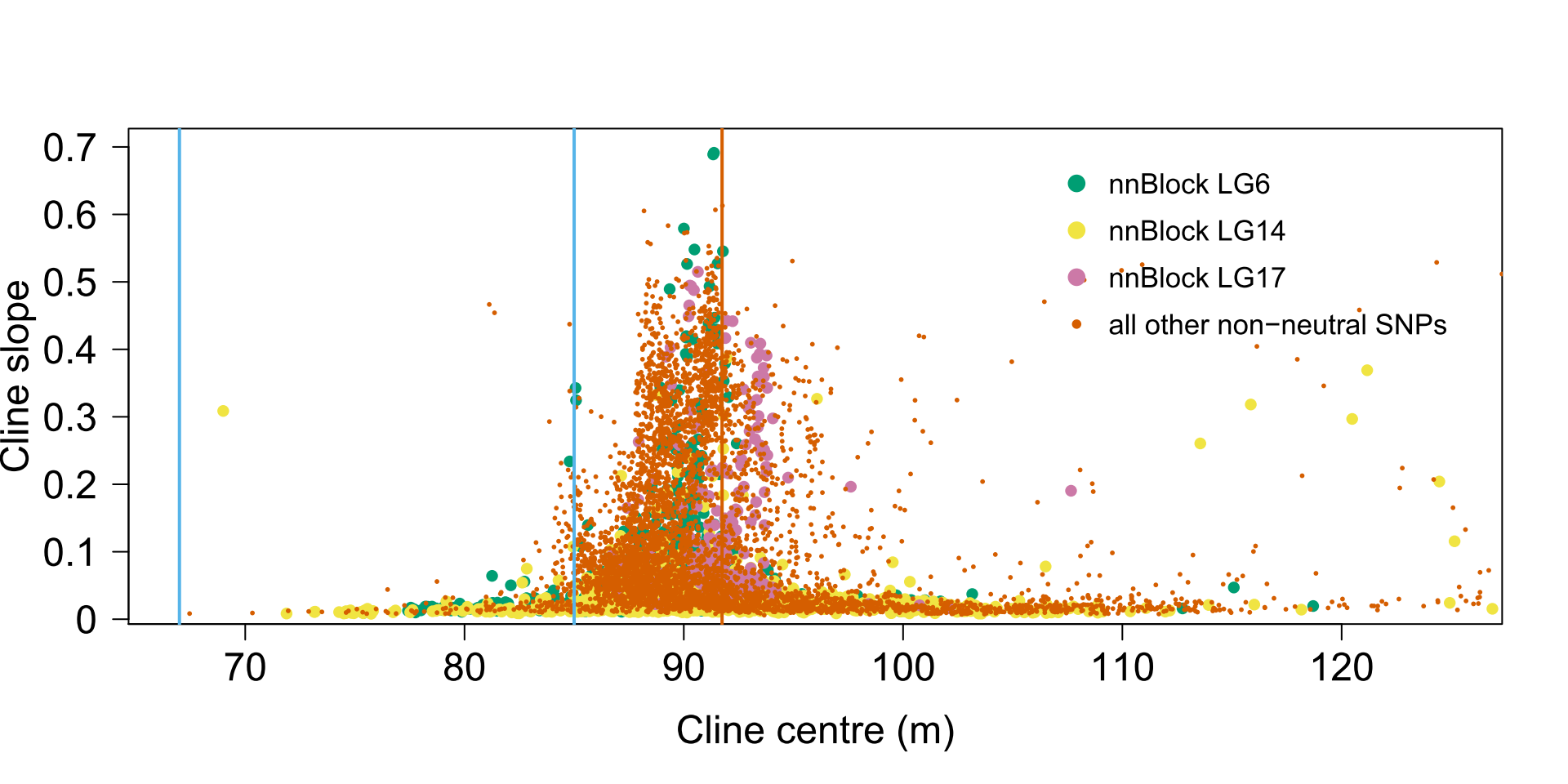


B)


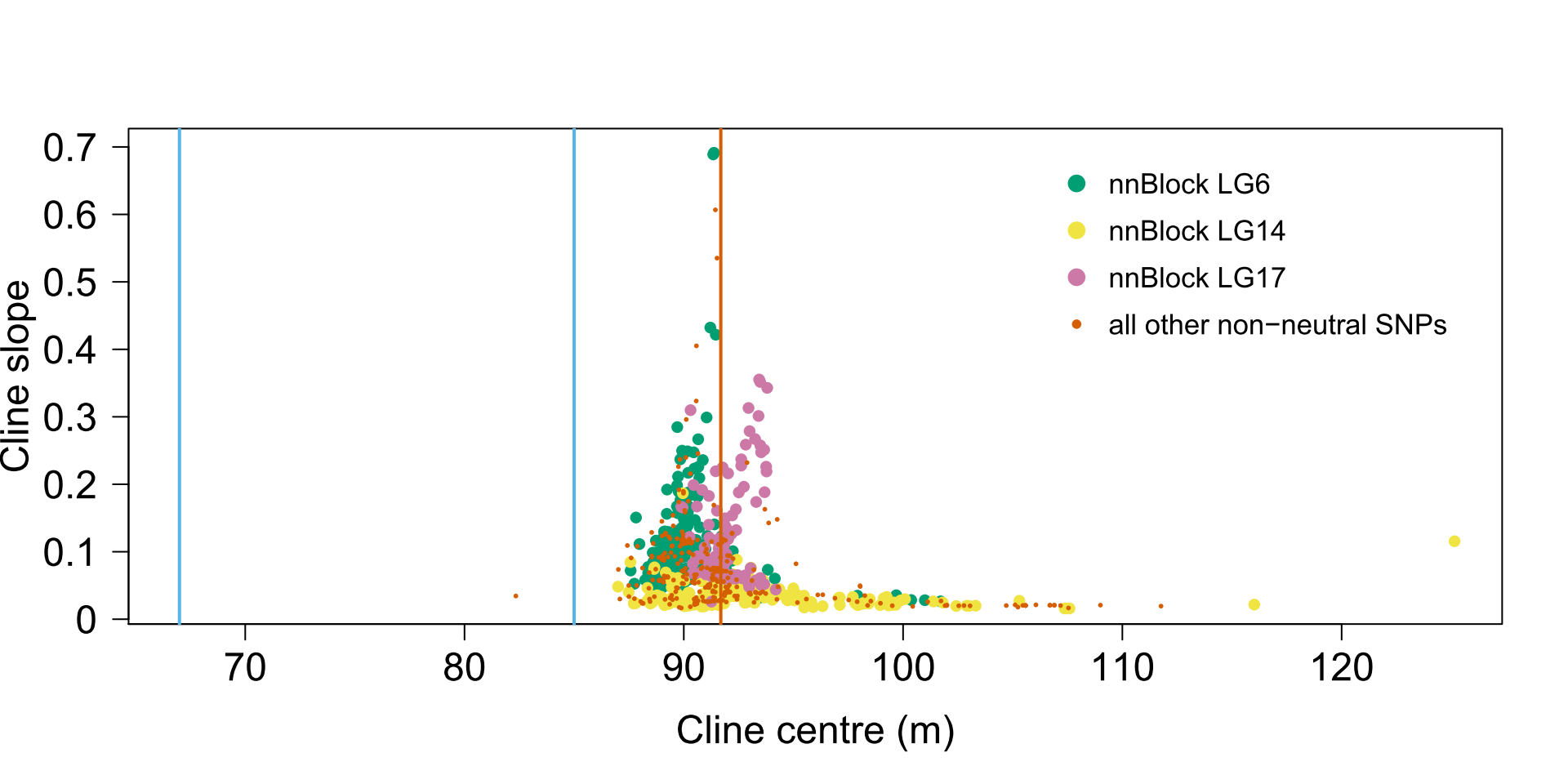


**Fig. S12**: Distribution of cline slopes and centres of non-neutral SNPs. Non-neutral clines in nnBlocks are shown in colours analogous to previous figures. All other non-neutral SNPs are shown in orange. The orange line indicates the average of all non-neutral cline centres. Blue lines indicate habitat transitions as in Fig. 1. A) var.ex threshold of 19.26. B) var.ex threshold of 47.48.

A)
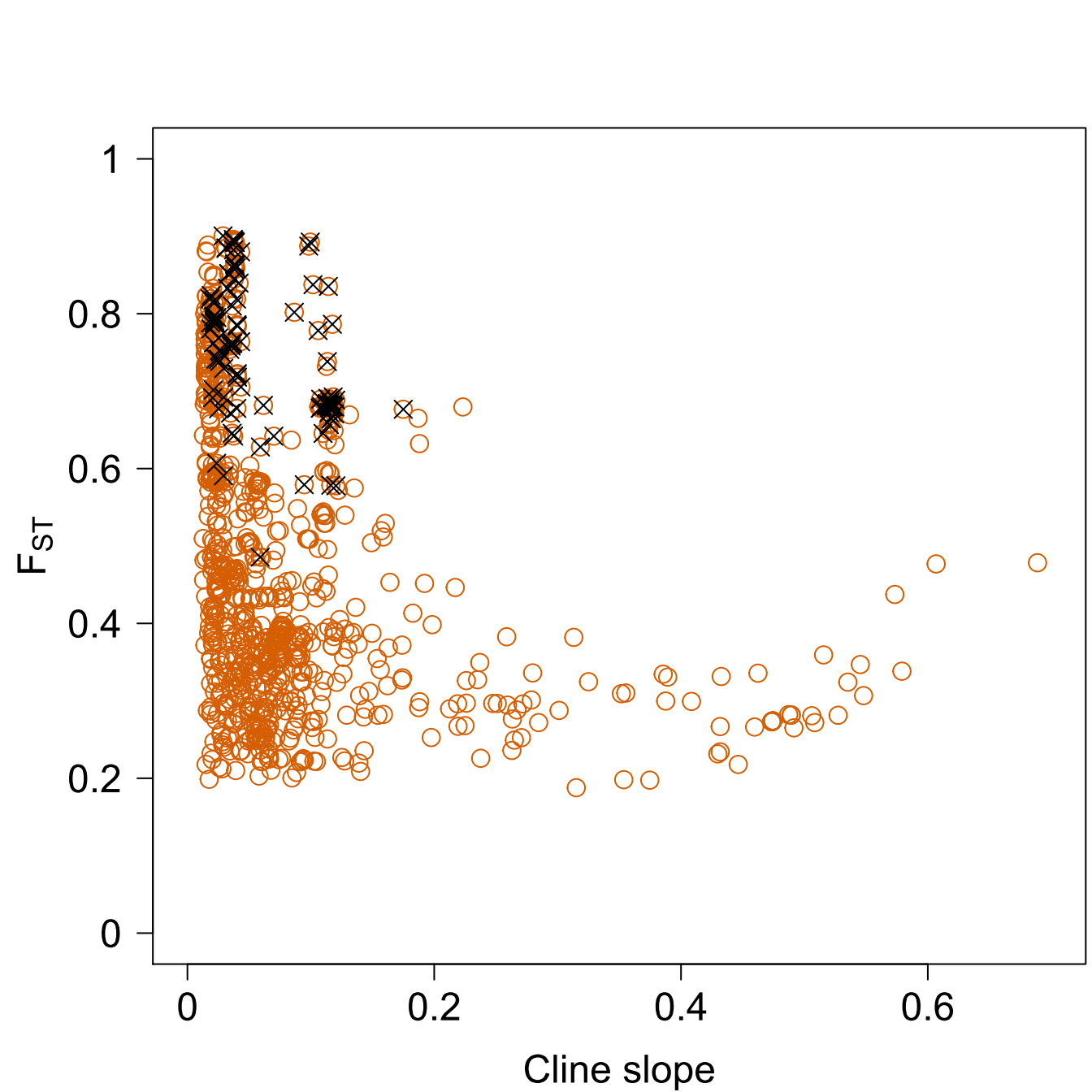
B)
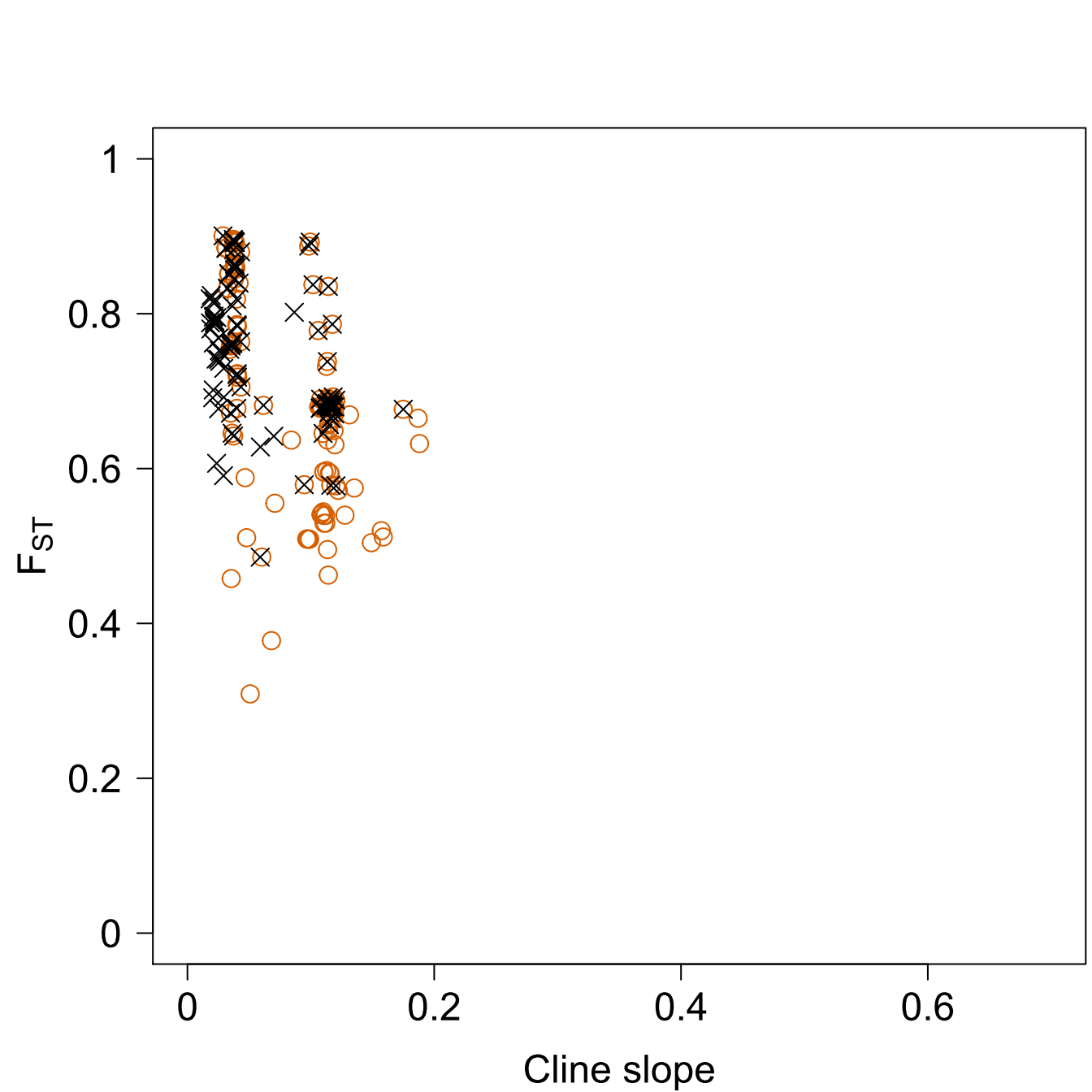


C)
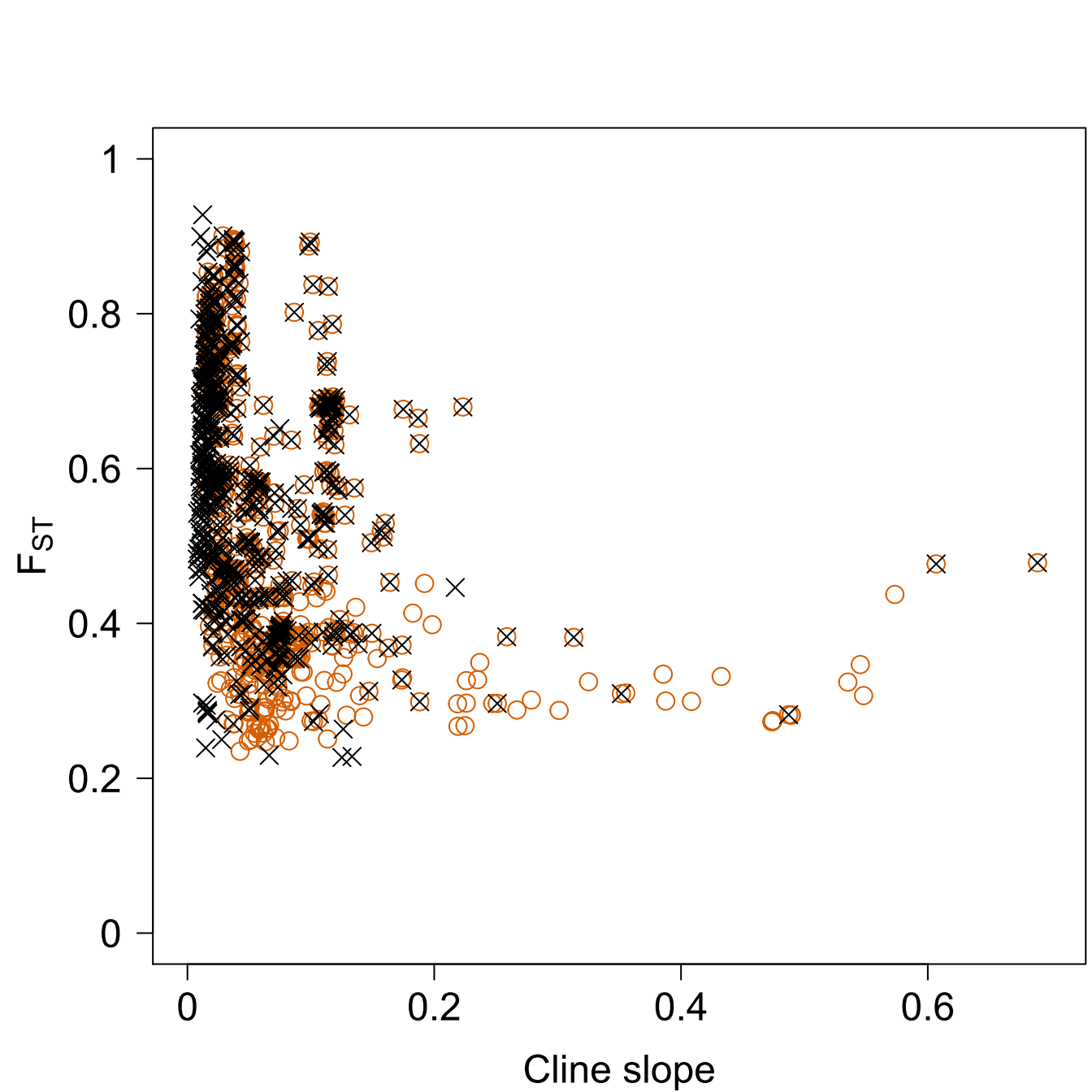


**Fig. S13**: Comparison between cline analysis and BayeScan outlier analysis. A) All cline outliers (orange circles; n=948, considering only SNPs used in both analyses) and all BayeScan outliers under standard settings (orange circles; n=143). 142 (99%) out of 143 BayeScan outliers were contained within the set of 948 non-neutral clinal SNPs. B) Standard (stringent) BayeScan analysis compared with the set of cline outliers obtained under enforced equal stringency. Black crosses: Non-neutral SNPs identified by BayeScan as those that had a q-value < 0.05 at a prior odds ratio of 1,000 (143 SNPs). Orange circles: the 143 non-neutral clinal loci with the highest var.ex. Overlap between the two sets of outliers was 70%. C) Lenient BayeScan analysis compared with the set of cline outliers obtained under enforced equal stringency. Black crosses: non-neutral SNPs identified by BayeScan as those that have a locus-specific F_ST_ estimate > 0.1 at a prior odds parameter of 100 (697 SNPs). Orange circles: the 697 non-neutral clinal loci with the highest var.ex. Overlap between the two sets of outliers was 73%.


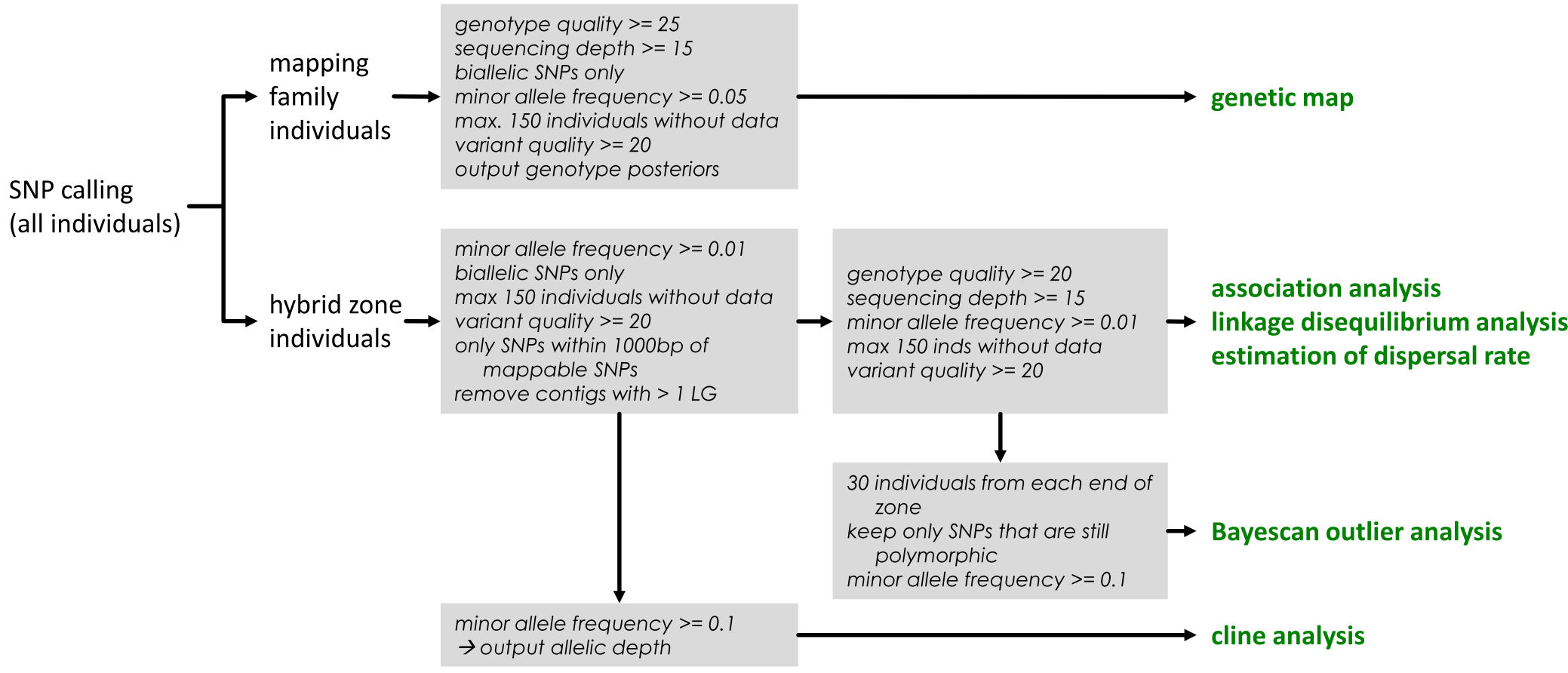


**Fig. S14**: Filtering of variant datasets after SNP calling. Filters were optimised for the following downstream analyses (green) and are shown in grey boxes. Standard filtering steps were performed in vcftools (Danecek *et al.* 2011); removal of SNPs distant from mapped SNPs and removal of contigs associated with multiple linkage groups was performed with custom R and bash scripts.

# SI Appendix, Tables

**Table S1**: Summary of sequencing libraries used for *Littorina saxatilis* assembly.

| Library | Average insert size (bp) | Read length (bp) | GC content | Total number  of bases | Trimming method |
| --- | --- | --- | --- | --- | --- |
| Littorina_1 | 112 | 101 | 42% | 46,912,679,172 | Trim Galore/Cutadapt |
| Littorina_2 | 203 | 101 | 42% | 14,208,904,220 | Trim Galore/Cutadapt |
| Littorina_3 | 206 | 101 | 42% | 30,439,678,354 | Trim Galore/Cutadapt |
| Littorina_4 | 208 | 101 | 41% | 20,848,408,890 | Trim Galore/Cutadapt |
| Littorina_5 | 209 | 101 | 42% | 37,976,024,442 | Trim Galore/Cutadapt |
| Littorina_6 | 345 | 126 | 41% | 68,613,002,892 | Trimmomatic |
| Littorina_7 | 355 | 126 | 41% | 56,077,755,300 | Trimmomatic |
| Littorina_8 | 520 | 300 | 42% | 101,061,858,282 | Trimmomatic |
| Littorina_9 | 528 | 300 | 42% | 74,348,777,104 | Trimmomatic |
| Littorina_10 | 1329 | 101 | 39% | 29,101,813,568 | Trim Galore/Cutadapt |
| Littorina_11 | 2564 | 101 | 41% | 37,689,441,992 | Trimmomatic |
| Littorina_12 | 5814 | 101 | 41% | 29,445,574,340 | Trimmomatic |
| Littorina_PB | 3,079* | 50-33,843 | 43% | 25,327,725,638 | Length cutoff 500bp |

* = Mean subread length given for PacBio data

**Table S2**: Summary of scaffolded genome assembly.

| Number of scaffolds: | 116,262 |
| --- | --- |
| N50 scaffolds: | 44,284 bp |
| NG50 scaffolds: | 55,450 bp |
| Average scaffold length: | 13,834 |
| Assembly length: | 1,608,389,317 |
| Gap content: | 4.8% |
| Min sequence length: | 1,000 |
| Max sequence length | 608,273 |

**Table S3**: Summary of BUSCO analyses of scaffolded assembly.

|  | Eukaryotic reference set | Metazoan reference set |
| --- | --- | --- |
| Complete BUSCOs | 239 (78.9%) | 784 (80.1%) |
| Complete and single-copy | 234 (77.2%) | 772 (78.9%) |
| Complete and duplicated | 5 (1.7%) | 12 (1.2%) |
| Fragmented BUSCOs | 31 (10.2%) | 111 (11.3%) |
| Missing BUSCOs | 33 (10.9%) | 83 (8.6%) |
| Total BUSCO groups | 303 | 978 |

**Table S4**: Parameter estimates (on the transformed scale) for size and shape clines. See Methods S1 for definitions of parameters. *hos* – height on shore. For centroid size, the best fitting model included a size difference between the sexes and an effect of shore height, both of which varied clinally (∆AIC = 38.55 compared to the next best model, which did not include the shore height effect; Fig. S1). For shape, the best fitting model included an influence of size, but not sex or height on the shore (∆AIC = 110.92 compared to the model with no size effect; Fig. S2). The size effect is consistent with previous observation of allometry that differs between ecotypes (Hollander *et al.* 2006). For size, the fit was improved by allowing variance to change along the transect, including an elevation at the cline centre, but variance was constant for shape.

| **Parameter** | **Size estimate** | **Standard error** | **Shape estimate** | **Standard error** |
| --- | --- | --- | --- | --- |
| *c* | 88.26 | 0.98 | 70.96 | 0.85 |
| *w* | 29.01 | 2.33 | 7.15 | 1.98 |
| *z_crab_* | 0.258 | 0.011 | 0.270 | 0.024 |
| *z_wave_* | 0.850 | 0.024 | 0.392 | 0.020 |
| *√v_crab_* | 0.060 | 0.0057 | 0.116 | 0.0037 |
| *√v_hybrid_* | 0.156 | 0.0117 | - | - |
| *√v_wave_* | 0.119 | 0.0092 | - | - |
| *b_crab_ (sex)* | -0.059 | 0.011 | - | - |
| *b_wave_ (sex)* | -0.151 | 0.025 | - | - |
| *b_crab_ (size)* | - | - | 0.077 | 0.123 |
| *b_wave_ (size)* | - | - | 0.436 | 0.032 |
| *b_crab_ (hos)* | 0.458 | 0.065 | - | - |
| *b_wave_ (hos)* | -0.077 | 0.109 | - | - |

**Table S5**: Parameter estimates (frequency on logit scale for *z* unless fixed at 0 or 1) for colour clines. See Methods S1 for definitions of parameters. NA – standard error could not be estimated. No fit was obtained for the ‘Other’ category. The overall frequencies of the different shell colour categories were: Beige 0.485, Dark beige 0.211, Banded 0.082, Black 0.075, Other 0.146 (based on 478 adult snails). Beige was dominant in the crab environment, while colour was much more variable in the wave environment.

| **Parameter** | **Beige estimate** | **Standard error** | **Dark beige estimate** | **Standard error** | **Black estimate** | **Standard error** | **Banded estimate** | **Standard error** |
| --- | --- | --- | --- | --- | --- | --- | --- | --- |
| *c* | 91.6 | NA | 68.0 | 12.8 | 100.0 | 8.00 | 94.8 | 2.28 |
| *w* | 0.46 | NA | 91.5 | 33.9 | 28.5 | 13.1 | 12.25 | 3.57 |
| *z_crab_* | 1.00 | NA | Set to 0 |  | Set to 0 |  | Set to 0 |  |
| *z_wave_* | Set to 0 |  | -0.65 | 0.32 | -0.917 | 0.384 | -0.99 | 0.22 |

**Table S6**: Different categories of SNPs based on cline analysis and simulations testing for neutrality. SNPs in grey were excluded from all downstream analyses. SNPs with small end frequency differences or without significant clinal change were categorised as neutral SNPs. SNPs with significant clines that did not deviate from neutral patterns observed in simulations were categorised as neutral as well. Only SNPs with significant clines that deviated from neutral patterns in simulations were labelled as non-neutral. SNPs “not clearly associated with any category” are based on inconsistency between jittered replicate cline fits for the same SNP, see Methods S5.

| **category (initial filters and cline analysis)** | **category (neutrality test based on sim.)** | **category in downstream analyses** | **number of SNPs** | **% of SNPs** |
| --- | --- | --- | --- | --- |
| sex-linked | NA | excluded | 2,275 | 1.55 |
| excess heterozygotes |  |  | 2,473 | 1.69 |
| central allele frequency peak |  |  | 23 | 0.02 |
| not clearly associated with any category |  |  | 3,882 | 2.65 |
| no clinal change (allele freq. difference < 0.1 or cline fit not significant) |  | neutral | 62,456 | 42.58 |
| significant cline | consistent with neutrality |  | 73,671 | 50.23 |
|  | not consistent with neutrality | **non-neutral** | 1,891 | 1.29 |

**Table S7**: Initial values, lower bounds and upper bounds for maximum likelihood estimation of SNP clines.

| Parameter | Description | Initial value* | Lower bound | Upper bound |
| --- | --- | --- | --- | --- |
| *p_crab_* | Crab end frequency | *e_c_* – initial estimate of Crab end frequency | 0.001 | 0.9 |
| *p_diff_* | Wave end frequency – Crab end frequency | *e_w_ - e_c_* , where *e_w_* is the initial estimate of Wave end frequency | 0.01 | 0.999-0.5*e_c_* |
| *c* | cline centre | Initial estimate of centre position – point of maximum heterozygote frequency | 1 | 150 |
| *w* | cline width | Initial estimate of width (Σ3*pq)*, based on division into 10m demes | 1 | 1.5*min(150-*c*, *c*-1) |
| *le* | ln(sequencing error) | -5 | -10 | -1 |
| *d_L_* | distance of the introgression tail to the left from the centre of the cline | *w* | 0 | 2*w* |
| *d_R_* | distance of the introgression tail to the right from the centre of the cline | *w* | 0 | 2*w* |
| τ_L_ | ratio of the slope of left tail to the slope of the central sigmoid function at the transition point | 0.999 | 0.001 | 1 |
| τ_R_ | ratio of the slope of right tail to the slope of the central sigmoid function at the transition point | 0.999 | 0.001 | 1 |

* The simple cline fit (first 5 parameters) was run twice. In the second run these initial values were replaced with the estimates from the first run. For the 5 common parameters, tailed cline fits were initiated with parameter estimates from the simple cline.

**Table S8**: Linkage disequilibrium (absolute correlation coefficient) between SNPs on linkage groups with regions showing high concentrations of non-neutral SNPs. Results are shown separately for SNPs inside and outside nnBlocks. The results demonstrate that linkage disequilibrium is elevated within nnBlocks in almost all cases (an exception is LG14 in the Wave ecotype), consistent with nnBlocks representing chromosomal rearrangements.

|  |  |  |  | **Mean and standard error of linkage disequilibrium** | | | | | |
| --- | --- | --- | --- | --- | --- | --- | --- | --- | --- |
| **LG** | **Ecotype** | **nnBlock** | **Number of SNPs** | **Between contigs, within map position** | **SE** | **Between map positions, within 5cM** | **SE** | **Between map positions, >5cM** | **SE** |
|  |  |  |  |  |  |  |  |  |  |
| 17 | Crab | Within | 270 | 0.166 | 0.0035 | 0.0740 | 0.00050 | 0.0700 | 0.00038 |
| 17 | Crab | Outside | 1553 | 0.134 | 0.00072 | 0.0787 | 0.00015 | 0.0699 | 5.4E-05 |
| 17 | Wave | Within | 323 | 0.445 | 0.0041 | 0.415 | 0.0020 | 0.415 | 0.0017 |
| 17 | Wave | Outside | 1313 | 0.208 | 0.00092 | 0.168 | 0.00032 | 0.145 | 0.00013 |
|  |  |  |  |  |  |  |  |  |  |
| 14 | Crab | Within | 1244 | 0.290 | 0.00079 | 0.259 | 0.00041 | 0.175 | 0.00035 |
| 14 | Crab | Outside | 550 | 0.180 | 0.0021 | 0.102 | 0.00043 | 0.083 | 0.00020 |
| 14 | Wave | Within | 851 | 0.198 | 0.00065 | 0.171 | 0.00031 | 0.162 | 0.00033 |
| 14 | Wave | Outside | 461 | 0.293 | 0.0028 | 0.283 | 0.0011 | 0.209 | 0.00059 |
|  |  |  |  |  |  |  |  |  |  |
| 6 | Crab | Within | 628 | 0.259 | 0.0021 | 0.273 | 0.0012 | 0.261 | 0.00064 |
| 6 | Crab | Outside | 1355 | 0.121 | 0.00049 | 0.0762 | 0.0001 | 0.0693 | 7.59E-05 |
| 6 | Wave | Within | 502 | 0.347 | 0.0026 | 0.341 | 0.0017 | 0.317 | 0.00089 |
| 6 | Wave | Outside | 1124 | 0.215 | 0.00075 | 0.171 | 0.00025 | 0.138 | 0.00018 |

**Table S9**: SNPs significantly associated with colour traits according to GenABEL analysis. Analyses included correction for population structure (first 4 principal components), and p-values were obtained by permutation. The position of the closest SNP in the genetic map is indicated for each colour-associated SNP. Linkage groups / positions in grey indicate that the closest SNP in the linkage map is more than 1000bp away. All other SNPs are included in Fig. S4. Note that only one colour-associated SNP was also included in the cline analysis (Contig77167, Position 4379); this SNP did not show a significant cline (column ”Type”).

**Table S10**: Contributions of linkage groups to the heritability of shell size and shape, estimated in HEIDI. The normalised contribution is the estimated proportion of variance attributable to SNPs on a linkage group. Significance was estimated by comparing the log-likelihood of a model with only a relatedness matrix derived from all SNPs to one that also including relatedness based on the specified linkage group, tested using χ^2^ = -2∆LL (1 d.f.).

|  |  | Shell size | | | Shell shape | | |
| --- | --- | --- | --- | --- | --- | --- | --- |
| Linkage group | Number of SNPs | Normalized contribution | -2∆LL | P-value | Normalized contribution | -2∆LL | P-value |
| 1 | 2717 | 0.014 | 0.007 | 0.932 | 0.021 | 0.252 | 0.616 |
| 2 | 1914 | <0.001 | 1.895 | 0.168 | <0.001 | 1.286 | 0.257 |
| 3 | 1486 | <0.001 | 1.581 | 0.208 | 0.050 | 0.086 | 0.769 |
| 4 | 1530 | <0.001 | 0.946 | 0.330 | 0.044 | 0.080 | 0.778 |
| 5 | 1216 | 0.019 | 1.072 | 0.300 | <0.001 | 1.964 | 0.161 |
| 6 | 1093 | 0.048 | 4.019 | 0.045 | 0.112 | 3.948 | 0.047 |
| 7 | 966 | <0.001 | 0.962 | 0.326 | 0.003 | 0.116 | 0.733 |
| 8 | 893 | <0.001 | 1.262 | 0.261 | <0.001 | 0.331 | 0.565 |
| 9 | 950 | <0.001 | 0.910 | 0.340 | 0.112 | 2.758 | 0.097 |
| 10 | 884 | <0.001 | 1.396 | 0.237 | 0.009 | 0.110 | 0.740 |
| 11 | 1113 | <0.001 | 0.403 | 0.525 | 0.016 | 0.001 | 0.974 |
| 12 | 973 | 0.121 | 59.020 | <0.001 | 0.054 | 0.872 | 0.350 |
| 13 | 829 | <0.001 | 0.692 | 0.405 | 0.020 | 0.006 | 0.939 |
| 14 | 638 | 0.017 | 0.877 | 0.349 | 0.075 | 0.525 | 0.469 |
| 15 | 684 | <0.001 | 0.748 | 0.387 | <0.001 | 0.485 | 0.486 |
| 16 | 461 | <0.001 | 0.852 | 0.355 | <0.001 | 0.615 | 0.433 |
| 17 | 612 | 0.065 | 2.696 | 0.100 | 0.095 | 2.204 | 0.138 |

**Table S11**: Numbers and proportions of clinal SNPs that were considered neutral and non-neutral. Non-neutral SNPs represent those that showed higher var.ex values than simulated neutral SNPs. Results are shown for three different var.ex thresholds identified by simulations. 35.69 was the threshold based on simulations with the set of parameters that seems most realistic given existing knowledge about the study system, and was used for the analyses shown in the main text; 19.26 and 47.48 represent the highest and lowest var.ex thresholds identified under a range of parameter values.

| **var.ex** | **19.26** | ***35.69*** | **47.48** |
| --- | --- | --- | --- |
| neutral | 68,500 (90.65%) | *73,671 (97.50%)* | 74,538 (98.64%) |
| non-neutral | 7,062 (9.35%) | *1,891 (2.50%)* | 1,024 (1.36%) |

# SI Appendix, References

Aulchenko, Y.S., Ripke, S., Isaacs, A., Duijn, V. & M, C. (2007). GenABEL: an R library for genome-wide association analysis. *Bioinformatics*, 23, 1294–1296.

Bolger, A.M., Lohse, M. & Usadel, B. (2014). Trimmomatic: a flexible trimmer for Illumina sequence data. *Bioinformatics*, 30, 2114–2120.

Bolker, B. (2012). R Development Core Team, 2014. bbmle: Tools for general maximum likelihood estimation. *R Package Version*.

Buerkle, A. & Gompert, Z. (2013). Population genomics based on low coverage sequencing: how low should we go? *Mol. Ecol.*, 22, 3028–3035.

Danecek, P., Auton, A., Abecasis, G., Albers, C.A., Banks, E., DePristo, M.A., *et al.* (2011). The variant call format and VCFtools. *Bioinformatics*, 27, 2156–2158.

Derryberry, E.P., Derryberry, G.E., Maley, J.M. & Brumfield, R.T. (2014). hzar: Hybrid zone analysis using an R software package. *Mol. Ecol. Resour.*, 14, 652–663.

English, A.C., Richards, S., Han, Y., Wang, M., Vee, V., Qu, J., *et al.* (2012). Mind the Gap: Upgrading Genomes with Pacific Biosciences RS Long-Read Sequencing Technology. *PLOS ONE*, 7, e47768.

Ewels, P., Magnusson, M., Lundin, S. & Käller, M. (2016). MultiQC: summarize analysis results for multiple tools and samples in a single report. *Bioinformatics*, 32, 3047–3048.

Foll, M. & Gaggiotti, O. (2008). A genome-scan method to identify selected loci appropriate for both dominant and codominant markers: a Bayesian perspective. *Genetics*, 180, 977–993.

Galindo, J., Grahame, J.W. & Butlin, R.K. (2010). An EST-based genome scan using 454 sequencing in the marine snail *Littorina saxatilis*. *J. Evol. Biol.*, 23, 2004–2016.

Gay, L., Crochet, P.-A., Bell, D.A. & Lenormand, T. (2008). Comparing clines on molecular and phenotypic traits in hybrid zones: a window on tension zone models. *Evolution*, 62, 2789–2806.

Hollander, J., Adams, D.C. & Johannesson, K. (2006). Evolution of adaptation through allometric shifts in a marine snail. *Evolution*, 60, 2490–2497.

Hollander, J., Galindo, J. & Butlin, R.K. (2015). Selection on outlier loci and their association with adaptive phenotypes in *Littorina saxatilis* contact zones. *J. Evol. Biol.*, 28, 328–337.

Kostem, E. & Eskin, E. (2013). Improving the accuracy and efficiency of partitioning heritability into the contributions of genomic regions. *Am. J. Hum. Genet.*, 92, 558–564.

Li, H. (2013). Aligning sequence reads, clone sequences and assembly contigs with BWA-MEM. *ArXiv13033997 Q-Bio*.

Li, H., Handsaker, B., Wysoker, A., Fennell, T., Ruan, J., Homer, N., *et al.* (2009). The Sequence Alignment/Map format and SAMtools. *Bioinformatics*, 25, 2078–2079.

Lindtke, D., González-Martínez, S.C., Macaya-Sanz, D. & Lexer, C. (2013). Admixture mapping of quantitative traits in *Populus* hybrid zones: power and limitations. *Heredity*, 111, 474.

Luo, R., Liu, B., Xie, Y., Li, Z., Huang, W., Yuan, J., *et al.* (2012). SOAPdenovo2: an empirically improved memory-efficient short-read de novo assembler. *GigaScience*, 1, 18.

Martin, M. (2011). Cutadapt removes adapter sequences from high-throughput sequencing reads. *EMBnet.journal*, 17, 10–12.

Money, D., Gardner, K., Migicovsky, Z., Schwaninger, H., Zhong, G.-Y. & Myles, S. (2015). Linkimpute: Fast and accurate genotype imputation for nonmodel organisms. *G3 Genes Genomes Genet.*, 5, 2383–2390.

Nadeau, N.J., Ruiz, M., Salazar, P., Counterman, B., Medina, J.A., Ortiz-Zuazaga, H., *et al.* (2014). Population genomics of parallel hybrid zones in the mimetic butterflies, *H. melpomene* and *H. erato*. *Genome Res.*, 24, 1316–1333.

Panova, M., Aronsson, H., Cameron, R.A., Dahl, P., Godhe, A., Lind, U., *et al.* (2016). DNA extraction protocols for whole-genome sequencing in marine organisms. In: *Marine Genomics*, Methods in Molecular Biology (ed. Bourlat, S.J.). Springer New York, pp. 13–44.

Price, A.L., Patterson, N.J., Plenge, R.M., Weinblatt, M.E., Shadick, N.A. & Reich, D. (2006). Principal components analysis corrects for stratification in genome-wide association studies. *Nat. Genet.*, 38, 904.

Pryszcz, L.P. & Gabaldón, T. (2016). Redundans: an assembly pipeline for highly heterozygous genomes. *Nucleic Acids Res.*, 44, e113–e113.

Rastas, P., Calboli, F.C.F., Guo, B., Shikano, T. & Merilä, J. (2016). Construction of ultradense linkage maps with Lep-MAP2: Stickleback F2 recombinant crosses as an example. *Genome Biol. Evol.*, 8, 78–93.

Rastas, P., Paulin, L., Hanski, I., Lehtonen, R. & Auvinen, P. (2013). Lep-MAP: fast and accurate linkage map construction for large SNP datasets. *Bioinformatics*, 29, 3128–3134.

Ravinet, M., Westram, A., Johannesson, K., Butlin, R., André, C. & Panova, M. (2016). Shared and nonshared genomic divergence in parallel ecotypes of *Littorina saxatilis* at a local scale. *Mol. Ecol.*, 25, 287–305.

Rieseberg, L.H. & Buerkle, C.A. (2002). Genetic mapping in hybrid zones. *Am. Nat.*, 159, S36–S50.

Rolan-Alvarez, E., Buño, I. & Gosalvez, J. (1996). Sex is determined by sex chromosomes in *Littorina saxatilis* (Olivi) (Gastropoda, Prosobranchia). *Hereditas*, 124, 261–268.

Simão, F.A., Waterhouse, R.M., Ioannidis, P., Kriventseva, E.V. & Zdobnov, E.M. (2015). BUSCO: assessing genome assembly and annotation completeness with single-copy orthologs. *Bioinformatics*, 31, 3210–3212.

Simpson, J.T. (2014). Exploring genome characteristics and sequence quality without a reference. *Bioinformatics*, 30, 1228–1235.

Vitturi, R., Libertini, A., Panozzo, M. & Mezzapelle, G. (1995). Karyotype analysis and genome size in three Mediterranean species of periwinkles (Prosobranchia: Mesogastropoda). *Malacologia*, 37, 123–132.

Westram, A.M., Galindo, J., Alm Rosenblad, M., Grahame, J.W., Panova, M. & Butlin, R.K. (2014). Do the same genes underlie parallel phenotypic divergence in different *Littorina saxatilis* populations? *Mol. Ecol.*, 23, 4603–4616.

Westram, A.M., Panova, M., Galindo, J. & Butlin, R.K. (2016). Targeted resequencing reveals geographical patterns of differentiation for loci implicated in parallel evolution. *Mol. Ecol.*, 25, 3169–3186.
