# Supplementary material for: Clines on the seashore: The genomic architecture underlying rapid divergence in the face of gene flow
Source: Evol Lett. 2018 Aug 7;2(4):297–309. doi: 10.1002/evl3.74 (PMC6121805; doi:10.1002/evl3.74)
Supplement: Supplementary file 22 — FIG. S1.1 Maximum‐likelihood estimates of cline centres for simulated allele‐frequency data under the primary divergence model with σ = 1.46. FIG. S1.2 Same as in Fig. S1.1, but for the secondary contact model. FIG. S1.3 Maximum‐likelihood estimates of the difference of allele frequencies at the two habitat ends for simulated allele‐frequency data under the primary divergence model with σ = 1.46. FIG. S1.4 Same as in Fig. S1.3, but for the secondary contact model. FIG. S1.5 Maximum‐likelihood estimates of cline width for simulated allele‐frequency data under the primary divergence model with σ = 1.46. FIG. S1.6 Same as in Fig. S1.5, but for the secondary contact model. FIG. S1.7 Variance explained by the maximum‐likelihood clinal fit for simulated allele‐frequency data under the primary divergence model with σ = 1.46. FIG. S1.8 Same as in Fig. S1.7, but for the secondary contact model. FIG. S1.9 Maximum‐likelihood estimates of cline slopes (a, c), and effective selection coefficients (per locus) inferred from the estimated slopes (b, d) for loci under selection. Shown are only results for loci designated as clinal under the primary divergence model with σ = 1.46. FIG. S1.10 Same as in Fig. S1.9, but for the secondary contact model. [file EVL3-2-297-s022.pdf]

Supporting Information S1

Clines on the seashore: The genomic architecture underlying rapid divergence in the face of gene flow

Anja M. Westram[1,2,3], Marina Rafajlović[4,5], Pragya Chaube[1], Rui Faria[1], Tomas Larsson[6], Marina Panova[7], Mark Ravinet[8], Anders Blomberg[9], Bernhard Mehlig[5], Kerstin Johannesson[7], Roger Butlin[1,7]

Addresses:
[1]Department of Animal and Plant Sciences, University of Sheffield, Sheffield, UK
[2]Current address: IST Austria, Am Campus 1, 3400 Klosterneuburg, Austria
[3]E-mail: anja.westram@ist.ac.at
[4]Current address: Department of Marine Sciences, University of Gothenburg, 40530 Gothenburg, Sweden
[5]Department of Physics, University of Gothenburg, 41296 Gothenburg, Sweden
[6]Department of Marine Sciences, University of Gothenburg, 40530 Gothenburg, Sweden
[7]Department of Marine Sciences - Tjärnö, University of Gothenburg, 45296 Strömstad, Sweden
[8]CEES (Centre for Ecological and Evolutionary Synthesis), University of Oslo, Oslo 0316, Norway
[9]Department of Chemistry and Molecular Biology, University of Gothenburg, 40530 Gothenburg, Sweden

Key words: : hybrid zones, clines, local adaptation, speciation, inversions, molluscs

Running title: Clines on the seashore

In this Supplementary document, we explain the implementation of the computer simulations used to interpret empirical data (sections Model and Methods), and we present the simulation results (section Results).

Recall that for each empirical marker SNP, a maximum-likelihood approach was used to determine the pattern ('clinal' or 'non-clinal'; see main text, Cline fitting and identification of non-neutral loci), and the maximum-likelihood parameters underlying the pattern that the SNP exhibited in our transect. Each SNP was initially designated as a non-clinal SNP. If the Akaike factor (AIC; (1)) for the maximum-likelihood fit for this SNP to a non-clinal pattern was larger by at least 4 than the corresponding AIC for the maximum-likelihood fit for this SNP to a cline, then the SNP was designated as a clinal SNP. Following this criterion, we found that many of the empirical marker SNPs were clinal (see Identification of non-neutral loci). Some of these clinal SNPs may be directly relevant to an individual's fitness (loci under some form of spatial selection gradient that imposes a barrier to gene flow), or under indirect selection due to linkage to such a SNP, but other loci are likely to be evolving neutrally and the clinal patterns they exhibit could be due to a the genome-wide effects of the barrier to gene flow, which may generate (weak) differentiation even at loci unlinked to selected regions. The effect of a barrier on a neutral locus is stronger and lasts longer when linkage to one or more directly selected loci is tighter (2). In principle, the different types of loci may be difficult to separate based on their clinal patterns. However, the genetic barrier is expected to have the weakest and shortest-lasting effect on neutral loci that are unlinked to any selected locus. Having this in mind, we aimed to use, as our neutral expectation, the clinal patterns at such loci, unlinked to any of the selected loci. We tested the empirical clinal patterns against this expectation (the statistic used is explained under Methods below). In essence, loci that are unlikely given this expectation may be under direct selection, or tightly linked to one or more selected loci (or they could be false positives, but the proportion of false positives is set in advance; see Methods below). Alternatively, such loci might indicate that the model used in our simulations lacks some properties relevant for the study system, e.g. the model does not include any density troughs in the sampling area, nor does it include habitat choice. However, we emphasise that in order to avoid this problem, we made the model as realistic for our study system as possible, representing an advance e.g. compared to outlier scan approaches that use a standard model uninformed by the study system.

To separate neutral loci that are not linked to any locus under selection from other types of loci (explained above), we used individual-based computer simulations of two models of divergence, that is, a model of a primary divergence and a model of a secondary contact. We used both divergence models to test for robustness of our method in identifying loci that are likely to be under selection or linked to a locus under selection (departures from our expectation), though for the *Littorina saxatilis* system, it has been found that a model of primary divergence is a better fit to observed patterns than a model of secondary contact (e.g. (3,4)). The two models we used are explained next (Model). Thereafter, in Methods, we explain the statistical test we used to compare the empirical data against the expectation. Finally, the simulation results are shown in Results.

S1.1. MODEL

A. General assumptions

The habitat was assumed to consist of K patches arranged in a one-dimensional array. The number of individuals in each patch was fixed to a constant denoted by N. We further assumed that there was an additional source population that consisted of one mainland (Primary divergence model) or two mainlands (Secondary contact model). In either case, the source population was assumed to be infinitely large and very old, so that the genetic variation it carried was not affected by potential migrants from any of the K patches, and that it was in a steady state. For more details on genetic variation carried by the source population, see Input data and parameters.

We assumed that migration occurred between neighbouring patches (short-range dispersal) according to the Gaussian dispersal function with mean zero and standard deviation σ. While migration from patches to the source population was neglected (see above), we assumed that the source population occasionally sent migrants to the patches by long-range dispersal, so that a migrant was equally likely to arrive at any patch in the habitat. The total long-range dispersal probability per generation from the source population was set to a constant denoted by M. In other words, the total number of migrants from the source population per generation was, on average, equal to M.

In the model, individuals were diploid. We assumed that the fitness of an individual depended on the individuals genotype at L bi-allelic loci under selection, as well as on the spatial position (patch $i = 1, 2, \ldots, K$) of the individual in the habitat. The two allele types at locus $j = 1, 2, \ldots, L$ are denoted by A_j and a_j. We assumed that there was a single environmental transition in the habitat (patch i_{T}) such that for individuals in patches $i \leq i_{\mathrm{T}}$ (i.e. *Crab-exposed* habitat), alleles A_j were beneficial over a_j (for all $j = 1, 2, \ldots, L$), whereas the opposite was true for individuals in patches $i > i_{\mathrm{T}}$ (i.e. *Wave-exposed* habitat). The selected loci were assumed to contribute to the fitness of an individual in a multiplicative way, so that the fitness $w_{(i,d)}$ of an individual in patch i, having d deleterious alleles and $2L - d$ beneficial alleles at the selected loci (note that due to diploidy, there are in total $2L$ alleles at the selected loci) was computed as

$$w_{(i,d)} = (1 - s_0)^d, \text{ for } 1 \leq i \leq K \text{ and } 0 \leq d \leq 2L \; . \tag{1}$$

Here, it was assumed that the deleterious allele at each selected locus contributed the same fitness disadvantage (s_0) to the overall fitness. Note that, according to Eq. (1), the maximum fitness locally in the habitat was equal to 1 (for $d = 0$), whereas the minimum fitness was equal to $(1 - s_0)^{2L}$. The maximum total fitness reduction (denoted by s below) anywhere in the habitat was, therefore, equal to $s = 1 - (1 - s_0)^{2L}$. In the limit of $s_0 \ll 1$, it follows that $s \approx 2Ls_0$.

The selected loci were assumed to be fully unlinked to each other, that is, we assumed that the recombination probability r_s per gamete per generation between any pair of loci under selection was $r_\mathrm{s} = 0.5$.

Apart from selected loci, in each individual we additionally modelled L_n bi-allelic neutral loci. The neutral loci were fully unlinked to any of the selected loci, so that the recombination probability r_sn between any selected and neutral locus was $r_\mathrm{sn} = 0.5$. By contrast, for pairs of neutral loci we set the per-gamete per-generation recombination probability using the empirical genetic map (see details below in Input data and parameters).

The lifecycle of individuals was modelled as follows: short-range dispersal of virgin adults between neighbouring patches, long-range dispersal of virgin adults from the source population, recombination, mating locally in each patch, and soft fecundity selection, so that the probability that an adult contributes to the pool of offspring in its patch is equal to the fitness of the adult relative to the total fitness of all adults in that patch.

The details of the two models we used (mimicking primary divergence and secondary contact) are explained next.

B. Primary divergence model

The source population in this model consisted of a single mainland. For neutral loci in our model (see Input data and parameters), the mainland was assumed to carry the genotypes of all individuals sampled empirically. In particular, from each multilocus genotype we generated pairs of haplotypes as follows. From a given empirically sampled multilocus genotype we generated a haplotype at the loci in question (recall that in each simulation we modelled L_n neutral loci; see above), by sampling at each locus uniformly at random an allele from the genotype (and the second haplotype corresponded to the sequence of alleles that were not chosen for the first haplotype). We repeated this with randomly-selected empirically-sampled genotypes until we generated enough pairs of haplotypes (each being an individual) to populate all patches in the habitat (see below). Conversely, for selected loci we assumed that the genetic variation at each locus corresponded to the empirically estimated average genetic variation at the L_n neutral loci modelled in the simulation in question. At the beginning of a simulation, for each patch, we sampled uniformly at random N individuals (genotypes) from the mainland, as explained above. Thereafter, we modelled short-range dispersal between neighbouring patches, long-range dispersal from the mainland, recombination, random mating locally in each patch, and soft selection. In the events of long-range dispersal from the mainland, we assumed that a migrant individual contributed at each locus with a given allele with a probability equal to the frequency of this allele at the locus in question.

C. Secondary contact model

The source population in this model consisted of two mainlands, referred to as the "Wave" and "Crab" mainland. For neutral loci in our model (see Input data and parameters), the Wave-mainland was assumed to carry the genotypes of 30 individuals sampled empirically from the Wave habitat end (and for the Crab-mainland, genotypes consisted of 30 individuals sampled from the Crab habitat end). By contrast, we assumed that in each mainland the corresponding locally beneficial allele was fixed at the selected loci. At the beginning of the simulation, for each patch with index i such the $i \leq i_\mathrm{T}$ we sampled uniformly at random N genotypes at neutral loci from the Crab mainland (the genotypes at the selected loci are fixed for the Crab-beneficial allele). Conversely, for each patch with index $i > i_\mathrm{T}$ we sampled uniformly at random N genotypes at neutral loci from the Wave mainland (the genotypes at the selected loci were fixed for the Wave-beneficial allele). Thereafter, we modelled short-range dispersal between neighbouring patches, long-range dispersal from the mainlands, recombination, random mating locally in each patch, and soft selection. In the events of long-range dispersal, we assumed that each mainland gave a migrant with probability $M/2$ per generation. Similarly to the primary divergence model, all patches were equally likely to receive a migrant, independently of whether the migrant came from the Wave or the Crab mainland, and the migrant individual contributed at each locus with a given allele with a probability equal to the frequency of this allele at the locus in question in the source mainland.

D. Input data and parameters

To cover in the simulations the range of recombination rates as well as of the amount of genetic variation relevant for the empirical data, we used the empirical genetic map, the individuals' genotypes at the loci sampled, as well as the empirical

distributions of the total number of read counts and of read-count ratios. The usage of these data in the simulations is explained next.

Firstly, for each simulation, we sampled uniformly at random a linkage group from the 17 linkage groups found in *L. saxatilis*. Then, we sampled uniformly at random (and without replacement) a number of contigs from this linkage group. The number of contigs to be sampled depended on the total number of neutral loci L_n in our simulations: from each contig, we took all SNP positions found on the contig to represent neutral loci in our simulation, and we continued sampling new contigs until we reached the designated number of neutral loci L_n. Note that here we only included empirical SNPs with less than 20% of missing genotypes. Then the recombination rates between pairs of neutral loci in the simulation in question were equal to the recombination rates between the corresponding SNP positions within and between the contigs sampled.

Secondly, the individuals' genotypes at the loci sampled for each simulation were taken to represent the genetic variation at these loci that was carried by the mainland in the primary divergence model, or by the two mainlands (Wave and Crab mainland) in the secondary contact model.

Finally, for the loci sampled for each simulation, we computed the distribution of the total number of reads, and the read-count ratio. This distribution of the total number of reads was used for sampling of simulated individuals as follows. For each individual sampled from a simulation in question, and for each locus, we first sampled randomly a number from the distribution of the total number of reads. If at a given locus an individual was a homozygote for one allele, then the read count for this allele was equal to the total number of reads sampled, and the read count for the alternative allele was equal to zero. Alternatively, if the individual was a heterozygote at this locus, the read count for an allele was sampled from a binomial distribution with the number of trials equal to the total number of read counts sampled, and the probability of success equal to the empirically estimated mean read-count ratio for this allele at this locus for heterozygotes. The latter was estimated using the empirical distribution of the read-count ratio for the allele at the locus in question based on the values of the read-count ratio that were larger than 0.1 and smaller than 0.9. Note that the values that were cut out may have been obtained in empirical sampling even when an individual was a homozygote due to potential sequencing errors. Such cases were, therefore, excluded from this estimation. In addition, we did not allow for sequencing errors in the simulations.

The number of individuals in each patch was fixed to $N = 100$, at the lower end of densities of adult snails observed in the field in order to approximate the effective density. However, we also performed simulations with a lower ($N = 50$), and a higher ($N = 200$) number of individuals per patch. The number of patches was fixed to $K = 152$. This number corresponded to the total length (in meters) of the sampled hybrid zone in question. Thus, each patch in our simulation represented a 1m wide area in the hybrid zone. The average dispersal distance was measured in meters, and it was taken from the empirically estimated dispersal distance ($\sigma = 1.46$, corresponding to the median across the estimated values from the linkage-disequilibrium patterns, see Methods S6). Additional simulations were ran with a lower ($\sigma = 1.09$), and a higher ($\sigma = 1.7$) value of σ, corresponding, respectively, to the 3rd and 48th ranked value among the estimated values of the average dispersal distance (see Methods S6). The number of neutral loci was fixed to $L_n = 200$, whereas the number of selected loci ranged between low ($L = 10$), intermediate ($L = 50$) and high ($L = 200$). Furthermore, the total fitness disadvantage (s) was set to $s = 0.7$, according to an earlier empirical estimate (5). The environmental transition occurred at patch number $i_{\mathrm{T}} = 85$, corresponding to the observed position of the main environmental transition at about 85m (see main text, Shore structure and phenotypic patterns). The long-range dispersal rate was set to a small value ($M = 0.1$). Finally, for each parameter set, we ran 200 simulations.

S1.2. METHODS

We ran each simulation for up to 8000 generations after the initialisation. We sampled individuals to assess the underlying clinal patterns at all loci in generations 1000, 2000, 4000 and 8000. Within the sets of parameters tested, the system reached approximate equilibrium after $2000 - 4000$ generations. Sampling was done to mimic as closely as possible empirical sampling: the number of individuals sampled from patch i in each simulation corresponded to the number of individuals sampled empirically between $(i - 1)$ metres and i meters in the hybrid zone. For each individual sampled, we generated read counts at the two loci (see Input data and parameters), applied the same filters as for the observed data, and used the resulting filtered data as input for cline fitting.

For each parameter set, and each sampling time, we fitted the sampled read counts to assess the maximum-likelihood parameters of the underlying pattern for each locus (clinal or non-clinal, see above). For both neutral and selected loci in our simulations, we estimated the probability distribution of cline centres, widths, end allele-frequencies, and variance explained (shown next in Results). We found that the neutral loci typically had much lower variance explained than the selected loci, and the overlap between the two distributions was very small ($< 0.005\%$), except when N was set to $N = 50$, in which case the overlap was higher (up to about 7%, not shown). Thus, our test statistic to distinguish between neutral loci unlinked to any selected locus and loci influenced by selection was based on the 99th percentile of the distribution of the variance explained found for the simulated neutral loci, where the variance explained for non-clinal neutral loci was set to zero (whereas loci for which fitting failed, that is, the variance explained was found to be negative and/or with $F_{\mathrm{ST}} > 1$, were excluded).

The simulation results obtained are shown in the next section.

S1.3. RESULTS

In this section the simulation results are organised as follows:

- percentage of clinal and non-clinal loci (Tabs. S1.1-S1.2, S1.4-S1.5, S1.7-S1.8),
- percentiles of variance explained (Tabs. S1.3, S1.6, S1.9),
- distributions of estimated cline centres (Figs. S1.1-S1.2),
- distribution of estimated differences in allele frequencies between the two habitat ends (Figs. S1.3-S1.4),
- distribution of estimated cline widths (Figs. S1.5-S1.6),
- distribution of the variance explained by the maximum-likelihood clinal parameters (Figs. S1.7-S1.8),
- distribution of cline slopes (adjusted by the difference in end frequencies) and effective selection per locus (Figs. S1.9-S1.10).

Note that, due to the large number of parameters' sets tested (see Input data and parameters), we do not show here the distributions of estimated cline parameters for all cases we tested. However, the corresponding summary statistics (Tabs. S1.1-S1.9) include all cases tested.

In what follows, the primary divergence model is referred to as "Model 1", and the secondary contact model is referred to as "Model 2".

TABLE S1.1 Summary statistics of the maximum-likelihood results for simulated allele-frequency data at neutrally evolving loci, with the number of individuals in each patch set to $N = 100$.

Neutrally Evolving Loci										
σ	Model	#Selected Loci[a]	Sampling Time	% Processed[b]	%Clinal Loci[c]				%Non-Clinal Loci[d]	
		L	T		Simple	Right Tail	Left Tail	Both Tails	$p_{\mathrm{d}} < 0.1$[e]	$p_{\mathrm{d}} > 0.1$
$\sigma = 1.46$	Model 1	$L = 10$	$T = 1000$	99.66	56.24	0.04	0.08	0.03	31.48	12.14
			$T = 2000$	99.42	60.68	0.09	0.08	0.04	28.59	10.52
			$T = 4000$	96.80	61.92	0.06	0.08	0.03	28.69	9.23
			$T = 8000$	88.60	61.64	0.06	0.09	0.05	29.36	8.79
		$L = 50$	$T = 1000$	99.69	57.08	0.06	0.06	0.03	30.80	11.97
			$T = 2000$	99.34	62.14	0.09	0.08	0.05	27.46	10.20
			$T = 4000$	96.70	64.73	0.08	0.08	0.08	26.39	8.65
			$T = 8000$	88.57	64.33	0.11	0.13	0.04	27.37	8.02
		$L = 200$	$T = 1000$	99.68	56.34	0.03	0.07	0.04	30.89	12.63
			$T = 2000$	99.42	63.24	0.09	0.09	0.04	26.72	9.81
			$T = 4000$	96.69	66.84	0.08	0.08	0.08	24.65	8.27
			$T = 8000$	88.43	66.58	0.08	0.13	0.07	25.70	7.43
	Model 2	$L = 10$	$T = 1000$	99.57	66.63	0.08	0.13	0.06	24.09	9.02
			$T = 2000$	98.85	64.48	0.11	0.11	0.07	26.35	8.89
			$T = 4000$	95.42	63.01	0.08	0.08	0.03	27.83	8.95
			$T = 8000$	87.08	61.62	0.08	0.09	0.04	29.65	8.51
		$L = 50$	$T = 1000$	99.59	67.53	0.12	0.17	0.08	23.22	8.89
			$T = 2000$	98.65	67.06	0.11	0.12	0.06	24.13	8.52
			$T = 4000$	95.58	65.48	0.09	0.12	0.05	26.16	8.10
			$T = 8000$	87.05	64.30	0.08	0.13	0.06	27.59	7.85
		$L = 200$	$T = 1000$	99.60	68.40	0.11	0.15	0.09	22.49	8.76
			$T = 2000$	98.83	68.11	0.11	0.10	0.07	23.48	8.14
			$T = 4000$	95.21	67.46	0.13	0.15	0.05	24.61	7.61
			$T = 8000$	86.68	67.12	0.10	0.12	0.07	25.09	7.51

[a]Per simulation.
[b]Percentage of all neutral loci that have passed our filters preceding fitting the data.
[c]Out of all processed neutral loci.
[d]Out of all processed neutral loci.
[e]p_{d} denotes the difference in allele frequencies at the two habitat ends.

TABLE S1.2 Summary statistics of the maximum-likelihood results for simulated allele-frequency data at loci under selection, with the number of individuals in each patch set to $N = 100$.

Loci Under Selection										
σ	Model	#Selected Loci[a] L	Sampling Time T	% Processed[b]	%Clinal Loci[c]				%Non-Clinal Loci[d]	
					Simple	Right Tail	Left Tail	Both Tails	$p_{\mathrm{d}} < 0.1$[e]	$p_{\mathrm{d}} > 0.1$
$\sigma = 1.46$	Model 1	$L = 10$	$T = 1000$	100.00	76.05	10.75	10.10	3.10	0.00	0.00
			$T = 2000$	100.00	75.50	10.75	10.90	2.85	0.00	0.00
			$T = 4000$	100.00	74.45	11.60	10.95	3.00	0.00	0.00
			$T = 8000$	100.00	76.60	11.10	9.45	2.85	0.00	0.00
		$L = 50$	$T = 1000$	100.00	91.31	2.99	3.63	2.07	0.00	0.00
			$T = 2000$	100.00	91.04	2.95	3.63	2.38	0.00	0.00
			$T = 4000$	100.00	91.37	2.75	3.67	2.21	0.00	0.00
			$T = 8000$	100.00	90.51	3.16	4.02	2.31	0.00	0.00
		$L = 200$	$T = 1000$	100.00	99.14	0.31	0.45	0.10	0.00	0.00
			$T = 2000$	100.00	98.44	0.59	0.66	0.32	0.00	0.00
			$T = 4000$	100.00	98.28	0.72	0.71	0.30	0.00	0.00
			$T = 8000$	100.00	98.30	0.70	0.71	0.28	0.00	0.00
	Model 2	$L = 10$	$T = 1000$	100.00	75.90	11.70	9.90	2.50	0.00	0.00
			$T = 2000$	100.00	77.35	10.20	9.90	2.55	0.00	0.00
			$T = 4000$	100.00	74.45	12.00	10.75	2.80	0.00	0.00
			$T = 8000$	100.00	77.75	9.40	10.00	2.85	0.00	0.00
		$L = 50$	$T = 1000$	100.00	91.07	3.18	3.36	2.39	0.00	0.00
			$T = 2000$	100.00	91.07	3.24	3.52	2.17	0.00	0.00
			$T = 4000$	100.00	91.16	2.74	3.69	2.41	0.00	0.00
			$T = 8000$	100.00	91.46	2.94	3.40	2.20	0.00	0.00
		$L = 200$	$T = 1000$	100.00	98.13	0.88	0.72	0.28	0.00	0.00
			$T = 2000$	100.00	98.19	0.79	0.71	0.30	0.00	0.00
			$T = 4000$	100.00	98.19	0.83	0.74	0.25	0.00	0.00
			$T = 8000$	100.00	98.27	0.75	0.67	0.32	0.00	0.00

[a]Per simulation.
[b]Percentage of all selected loci that have passed our filters preceding fitting the data.
[c]Out of all processed selected loci.
[d]Out of all processed selected loci.
[e]p_{d} denotes the difference in allele frequencies at the two habitat ends.

TABLE S1.3 Percentiles of the variance explained by the maximum-likelihood clinal fits for simulated neutral loci (null hypothesis), with the number of individuals in each patch set to $N = 100$. The variance explained for loci designated as *non-clinal* was set to 0. Loci for which the variance explained was found to be negative, or with $F_{\mathrm{ST}} > 1$ were excluded. The expectation used for the results shown in the main text is shown in boldface and highlighted by a star.

σ	Model	#Selected Loci[a] L	Sampling Time T	Percentile 95	Percentile 99
$\sigma = 1.46$	Model 1	$L = 10$	$T = 1000$	14.13	21.52
			$T = 2000$	17.07	25.69
			$T = 4000$	19.56	29.16
			$T = 8000$	19.98	30.05
		$L = 50$	$T = 1000$	14.72	22.31
			$T = 2000$	18.94	28.48
			$T = 4000$	22.41	33.46
			$T = 8000$	23.01	33.60
		$L = 200$	$T = 1000$	13.93	21.58
			$T = 2000$	19.49	29.26
			$T = 4000$	24.27	**35.69***
			$T = 8000$	26.39	37.65
	Model 2	$L = 10$	$T = 1000$	20.93	31.16
			$T = 2000$	20.18	29.57
			$T = 4000$	20.17	29.29
			$T = 8000$	20.26	29.93
		$L = 50$	$T = 1000$	22.92	34.51
			$T = 2000$	23.11	34.24
			$T = 4000$	23.16	33.95
			$T = 8000$	23.29	35.24
		$L = 200$	$T = 1000$	23.42	35.40
			$T = 2000$	24.10	34.95
			$T = 4000$	25.16	37.29
			$T = 8000$	25.77	37.78

[a]Per simulation.

TABLE S1.4 Same as in Tab. S1.1 but for two additional values of the dispersal distance σ: $\sigma = 1.09$ and $\sigma = 1.70$. In both cases, the primary divergence model (i.e. Model 1) was simulated, and the number of individuals in each patch was set to $N = 100$.

Neutrally Evolving Loci										
σ	Model	#Selected Loci[a] L	Sampling Time T	% Processed[b]	%Clinal Loci[c]				%Non-Clinal Loci[d]	
					Simple	Right Tail	Left Tail	Both Tails	$p_{\mathrm{d}} < 0.1$[e]	$p_{\mathrm{d}} > 0.1$
$\sigma = 1.09$	Model 1	$L = 10$	$T = 1000$	98.77	59.59	0.10	0.11	0.05	30.94	9.22
			$T = 2000$	98.47	65.14	0.11	0.17	0.07	26.89	7.62
			$T = 4000$	96.30	68.25	0.22	0.19	0.11	26.76	6.47
			$T = 8000$	88.84	68.25	0.25	0.19	0.11	25.18	6.03
		$L = 50$	$T = 1000$	98.99	59.91	0.08	0.07	0.05	30.48	9.41
			$T = 2000$	98.59	66.24	0.17	0.15	0.07	25.55	7.82
			$T = 4000$	96.03	69.62	0.23	0.24	0.10	23.62	6.20
			$T = 8000$	88.52	70.43	0.27	0.27	0.13	23.43	5.46
		$L = 200$	$T = 1000$	99.12	60.55	0.07	0.10	0.07	29.79	9.43
			$T = 2000$	98.58	67.37	0.12	0.15	0.07	25.11	7.19
			$T = 4000$	96.28	70.46	0.22	0.22	0.10	22.99	6.01
			$T = 8000$	87.85	72.07	0.30	0.27	0.11	22.13	5.11
$\sigma = 1.70$	Model 1	$L = 10$	$T = 1000$	99.79	52.82	0.05	0.06	0.02	33.31	13.74
			$T = 2000$	99.48	57.85	0.04	0.06	0.03	29.82	12.19
			$T = 4000$	96.97	58.61	0.06	0.07	0.02	29.86	11.37
			$T = 8000$	89.05	56.97	0.06	0.06	0.02	32.28	10.61
		$L = 50$	$T = 1000$	99.86	54.14	0.03	0.06	0.03	32.29	13.45
			$T = 2000$	99.42	59.94	0.04	0.05	0.04	28.23	11.70
			$T = 4000$	96.84	60.73	0.06	0.07	0.04	28.56	10.54
			$T = 8000$	88.40	60.14	0.08	0.06	0.05	29.66	10.02
		$L = 200$	$T = 1000$	99.86	52.93	0.04	0.06	0.04	32.71	14.22
			$T = 2000$	99.43	60.43	0.04	0.06	0.02	27.81	11.64
			$T = 4000$	96.94	62.88	0.06	0.09	0.05	26.92	10.00
			$T = 8000$	88.14	61.99	0.07	0.10	0.04	28.35	9.45

[a]Per simulation.
[b]Percentage of all neutral loci that have passed our filters preceding fitting the data.
[c]Out of all processed neutral loci.
[d]Out of all processed neutral loci.
[e]p_{d} denotes the difference in allele frequencies at the two habitat ends.

TABLE S1.5 Same as in Tab. S1.4 but for loci under selection.

Loci Under Selection										
σ	Model	#Selected Loci[a] L	Sampling Time T	% Processed[b]	%Clinal Loci[c]				%Non-Clinal Loci[d]	
					Simple	Right Tail	Left Tail	Both Tails	$p_{\mathrm{d}} < 0.1$[e]	$p_{\mathrm{d}} > 0.1$
$\sigma = 1.09$	Model 1	$L = 10$	$T = 1000$	100.00	75.00	10.15	11.10	3.75	0.00	0.00
			$T = 2000$	100.00	75.40	10.35	11.50	2.75	0.00	0.00
			$T = 4000$	100.00	75.10	10.45	11.75	2.70	0.00	0.00
			$T = 8000$	100.00	74.55	11.80	10.80	2.85	0.00	0.00
		$L = 50$	$T = 1000$	100.00	86.01	4.99	5.21	3.79	0.00	0.00
			$T = 2000$	100.00	86.19	4.86	4.83	4.12	0.00	0.00
			$T = 4000$	100.00	86.22	4.64	5.54	3.60	0.00	0.00
			$T = 8000$	100.00	85.59	5.08	5.32	4.01	0.00	0.00
		$L = 200$	$T = 1000$	100.00	98.14	0.88	0.79	0.18	0.00	0.00
			$T = 2000$	100.00	95.54	2.35	1.41	0.70	0.00	0.00
			$T = 4000$	100.00	94.73	2.74	1.58	0.95	0.00	0.00
			$T = 8000$	100.00	94.82	2.74	1.53	0.91	0.00	0.00
$\sigma = 1.70$	Model 1	$L = 10$	$T = 1000$	100.00	78.85	9.50	8.55	3.10	0.00	0.00
			$T = 2000$	100.00	79.05	9.75	8.50	2.70	0.00	0.00
			$T = 4000$	100.00	76.25	10.75	9.40	3.60	0.00	0.00
			$T = 8000$	100.00	78.85	8.70	9.40	3.05	0.00	0.00
		$L = 50$	$T = 1000$	100.00	92.79	2.48	3.01	1.72	0.00	0.00
			$T = 2000$	100.00	93.17	2.55	2.81	1.47	0.00	0.00
			$T = 4000$	100.00	93.75	2.40	2.58	1.27	0.00	0.00
			$T = 8000$	100.00	93.26	2.49	2.75	1.50	0.00	0.00
		$L = 200$	$T = 1000$	100.00	99.46	0.20	0.30	0.05	0.00	0.00
			$T = 2000$	100.00	99.18	0.24	0.46	0.12	0.00	0.00
			$T = 4000$	100.00	99.10	0.28	0.46	0.16	0.00	0.00
			$T = 8000$	100.00	99.10	0.24	0.47	0.18	0.00	0.00

[a]Per simulation.
[b]Percentage of all neutral loci that have passed our filters preceding fitting the data.
[c]Out of all processed neutral loci.
[d]Out of all processed neutral loci.
[e]p_{d} denotes the difference in allele frequencies at the two habitat ends.

TABLE S1.6 Same as in Tab. S1.3 but for two additional values of the dispersal distance σ: $\sigma = 1.09$ and $\sigma = 1.70$. In both cases, the primary divergence model (i.e. Model 1) was simulated, and the number of individuals in each patch set to $N = 100$.

σ	Model	#Selected Loci[a] L	Sampling Time T	Percentile 95	Percentile 99
$\sigma = 1.09$	Model 1	$L = 10$	$T = 1000$	16.70	25.38
			$T = 2000$	21.54	31.48
			$T = 4000$	25.95	37.25
			$T = 8000$	27.58	39.22
		$L = 50$	$T = 1000$	16.86	25.37
			$T = 2000$	22.86	33.92
			$T = 4000$	27.91	40.40
			$T = 8000$	30.95	43.92
		$L = 200$	$T = 1000$	16.93	25.66
			$T = 2000$	23.20	34.17
			$T = 4000$	30.01	43.25
			$T = 8000$	35.04	47.48
$\sigma = 1.70$	Model 1	$L = 10$	$T = 1000$	12.68	19.42
			$T = 2000$	15.31	22.87
			$T = 4000$	16.79	25.29
			$T = 8000$	16.27	25.07
		$L = 50$	$T = 1000$	13.36	20.81
			$T = 2000$	17.30	25.61
			$T = 4000$	19.21	29.33
			$T = 8000$	19.70	29.00
		$L = 200$	$T = 1000$	12.60	19.26
			$T = 2000$	17.28	25.54
			$T = 4000$	20.42	30.23
			$T = 8000$	20.73	31.66

[a]Per simulation.

TABLE S1.7 Same as in Tab. S1.1 but for two additional values of the local population size N: $N = 50$ and $N = 200$. In both cases, the primary divergence model (i.e. Model 1) with $\sigma = 1.46$ and $L = 200$ was simulated.

Neutrally Evolving Loci										
N	Model	#Selected Loci[a] L	Sampling Time T	% Processed[b]	%Clinal Loci[c]				%Non-Clinal Loci[d]	
					Simple	Right Tail	Left Tail	Both Tails	$p_\mathrm{d} < 0.1$[e]	$p_\mathrm{d} > 0.1$
$N = 50$	Model 1	$L = 200$	$T = 1000$	98.38	67.61	0.17	0.15	0.10	24.85	7.12
			$T = 2000$	95.99	71.98	0.30	0.31	0.10	21.88	5.43
			$T = 4000$	88.02	73.65	0.44	0.37	0.18	20.88	4.48
			$T = 8000$	72.48	72.31	0.44	0.35	0.18	22.78	3.94
$N = 200$	Model 1	$L = 200$	$T = 1000$	100.00	41.13	0.01	0.02	0.02	39.76	19.07
			$T = 2000$	99.94	49.90	0.03	0.03	0.02	33.43	16.60
			$T = 4000$	99.48	55.04	0.02	0.04	0.02	30.45	14.44
			$T = 8000$	96.67	55.63	0.05	0.04	0.02	30.95	13.31

[a] Per simulation.
[b] Percentage of all neutral loci that have passed our filters preceding fitting the data.
[c] Out of all processed neutral loci.
[d] Out of all processed neutral loci.
[e] p_d denotes the difference in allele frequencies at the two habitat ends.

TABLE S1.8 Same as in Tab. S1.7 but for loci under selection.

Loci Under Selection										
N	Model	#Selected Loci[a] L	Sampling Time T	% Processed[b]	%Clinal Loci[c]				%Non-Clinal Loci[d]	
					Simple	Right Tail	Left Tail	Both Tails	$p_{\mathrm{d}} < 0.1$[e]	$p_{\mathrm{d}} > 0.1$
$N = 50$	Model 1	$L = 200$	$T = 1000$	100.00	97.89	1.12	0.77	0.20	0.01	0.00
			$T = 2000$	100.00	97.14	1.44	0.94	0.47	0.00	0.00
			$T = 4000$	100.00	96.84	1.66	0.94	0.56	0.00	0.00
			$T = 8000$	100.00	96.84	1.66	0.92	0.58	0.00	0.00
$N = 200$	Model 1	$L = 200$	$T = 1000$	100.00	99.62	0.10	0.22	0.06	0.00	0.00
			$T = 2000$	100.00	99.11	0.20	0.52	0.17	0.00	0.00
			$T = 4000$	100.00	98.93	0.32	0.53	0.22	0.00	0.00
			$T = 8000$	100.00	98.91	0.35	0.54	0.21	0.00	0.00

[a]Per simulation.
[b]Percentage of all neutral loci that have passed our filters preceding fitting the data.
[c]Out of all processed neutral loci.
[d]Out of all processed neutral loci.
[e]p_{d} denotes the difference in allele frequencies at the two habitat ends.

TABLE S1.9 Same as in Tab. S1.3 but for two additional values of the local population size N: $N = 50$ and $N = 200$. In both cases, the primary divergence model (i.e. Model 1) with $\sigma = 1.46$ and $L = 200$ was simulated.

N	Model	#Selected Loci[a] L	Sampling Time T	Percentile	
				95	99
$N = 50$	Model 1	$L = 200$	$T = 1000$	23.50	34.67
			$T = 2000$	31.61	45.01
			$T = 4000$	38.01	52.54
			$T = 8000$	41.04	55.72
$N = 200$	Model 1	$L = 200$	$T = 1000$	8.43	13.21
			$T = 2000$	11.63	18.17
			$T = 4000$	14.17	21.87
			$T = 8000$	15.47	23.55

[a]Per simulation.

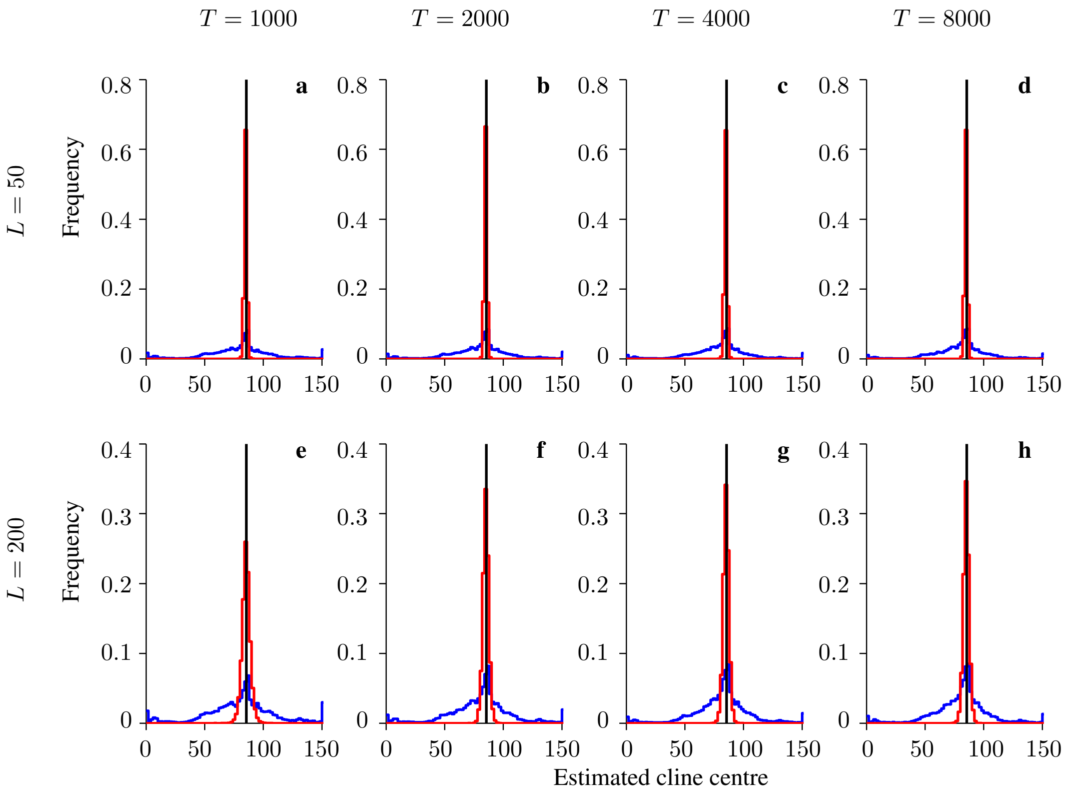

FIG. S1.1 Maximum-likelihood estimates of cline centres for simulated allele-frequency data under the primary divergence model with $\sigma = 1.46$. Shown are results for neutral loci (blue), and selected loci (red). Black line corresponds to the position of the change of selection pressure in the simulated habitat. Number of loci under selection: $L = 50$ (**a**-**d**), $L = 200$ (**e**-**h**). Panels differ by the sampling time: $T = 1000$ (**a**, **e**), $T = 2000$ (**b**, **f**), $T = 4000$ (**c**, **g**), and $T = 8000$ (**d**, **h**). In all cases, the number of individuals in each patch was set to $N = 100$.

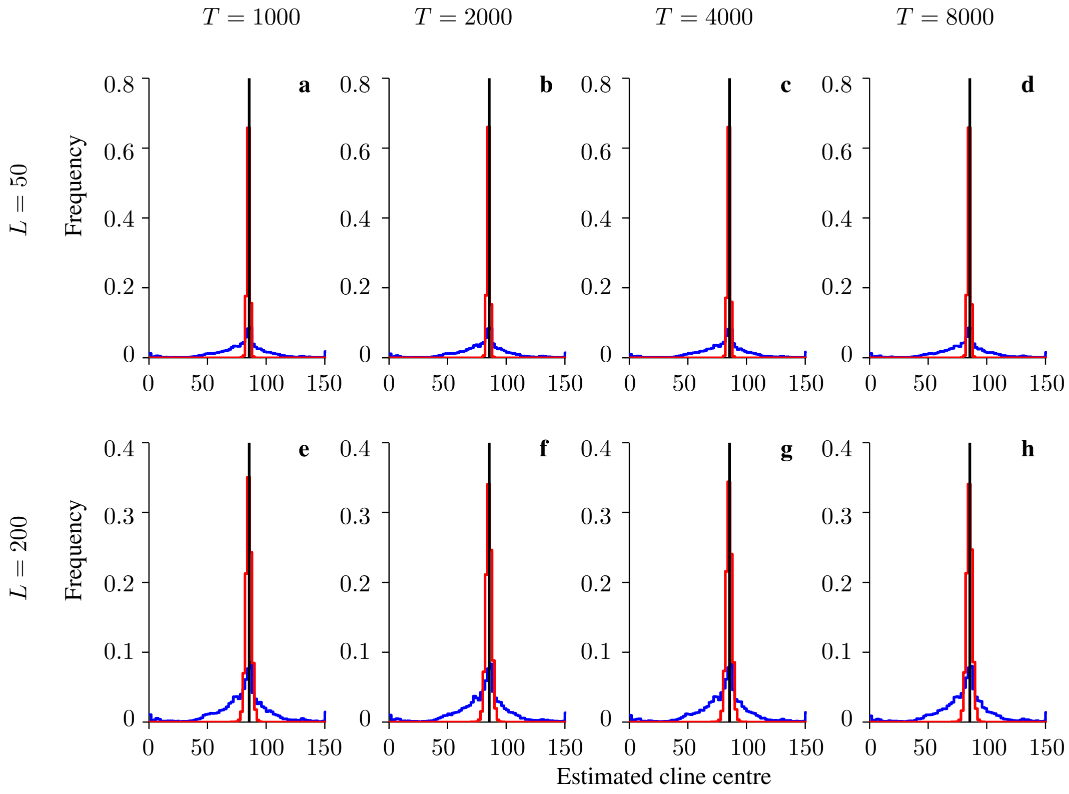

FIG. S1.2 Same as in Fig. S1.1, but for the secondary contact model.

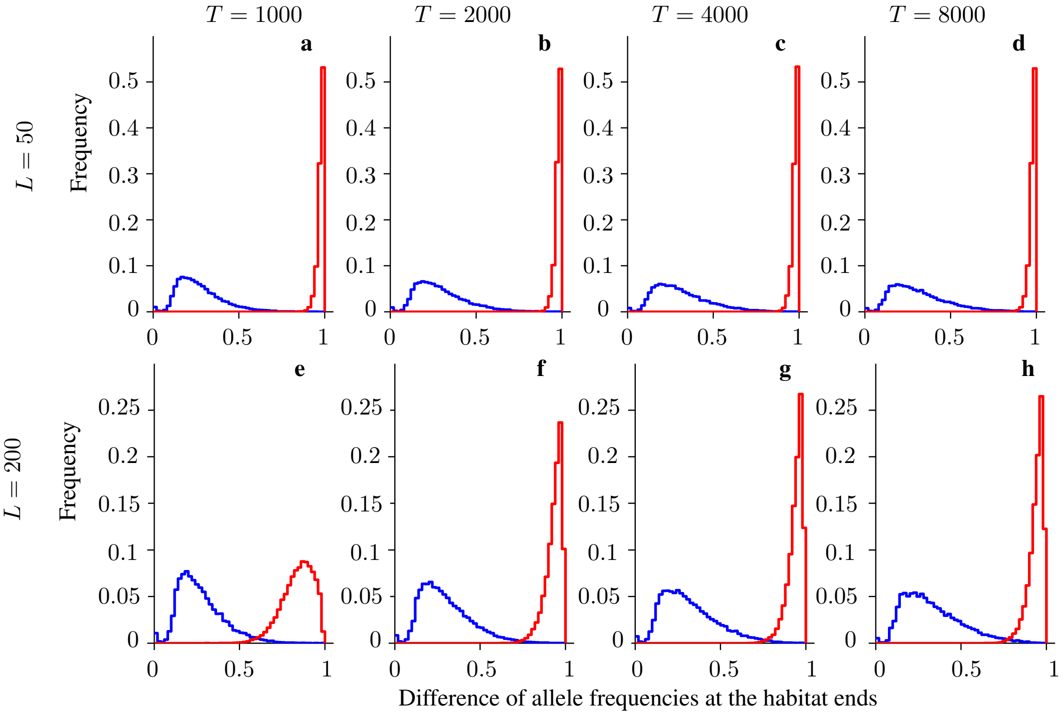

FIG. S1.3 Maximum-likelihood estimates of the difference of allele frequencies at the two habitat ends for simulated allele-frequency data under the primary divergence model with $\sigma = 1.46$. Shown are results for neutral loci (blue), and selected loci (red). Number of loci under selection: $L = 50$ (**a**-**d**), $L = 200$ (**e**-**h**). Panels differ by the sampling time: $T = 1000$ (**a**, **e**), $T = 2000$ (**b**, **f**), $T = 4000$ (**c**, **g**), and $T = 8000$ (**d**, **h**). In all cases, the number of individuals in each patch was set to $N = 100$.

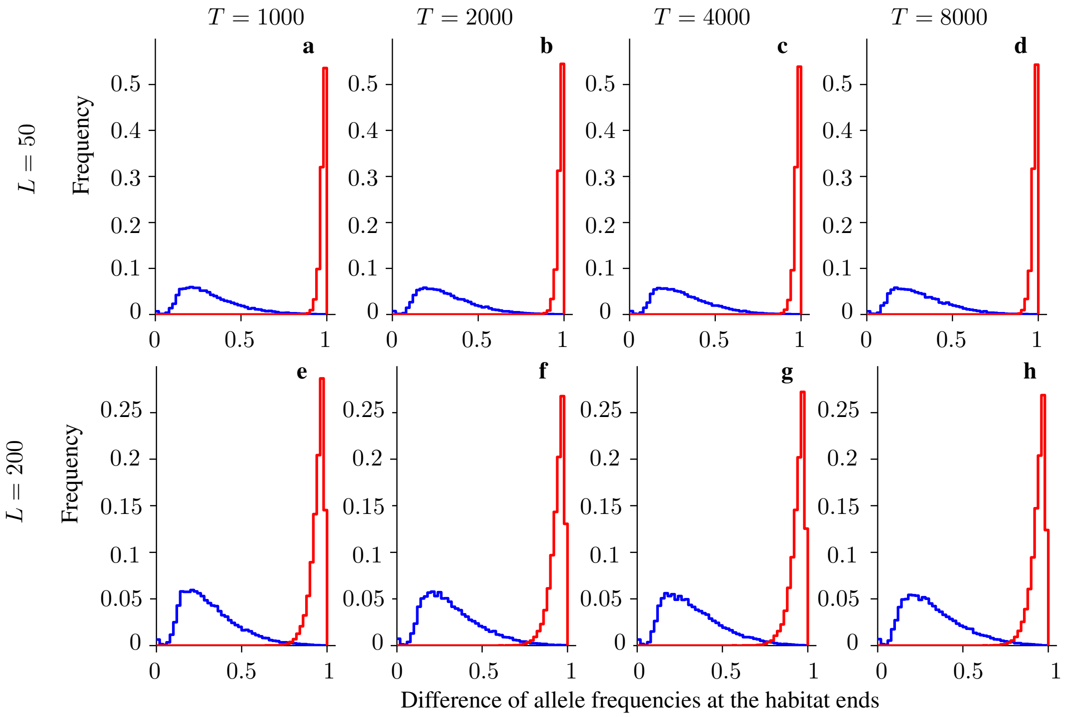

FIG. S1.4 Same as in Fig. S1.3, but for the secondary contact model.

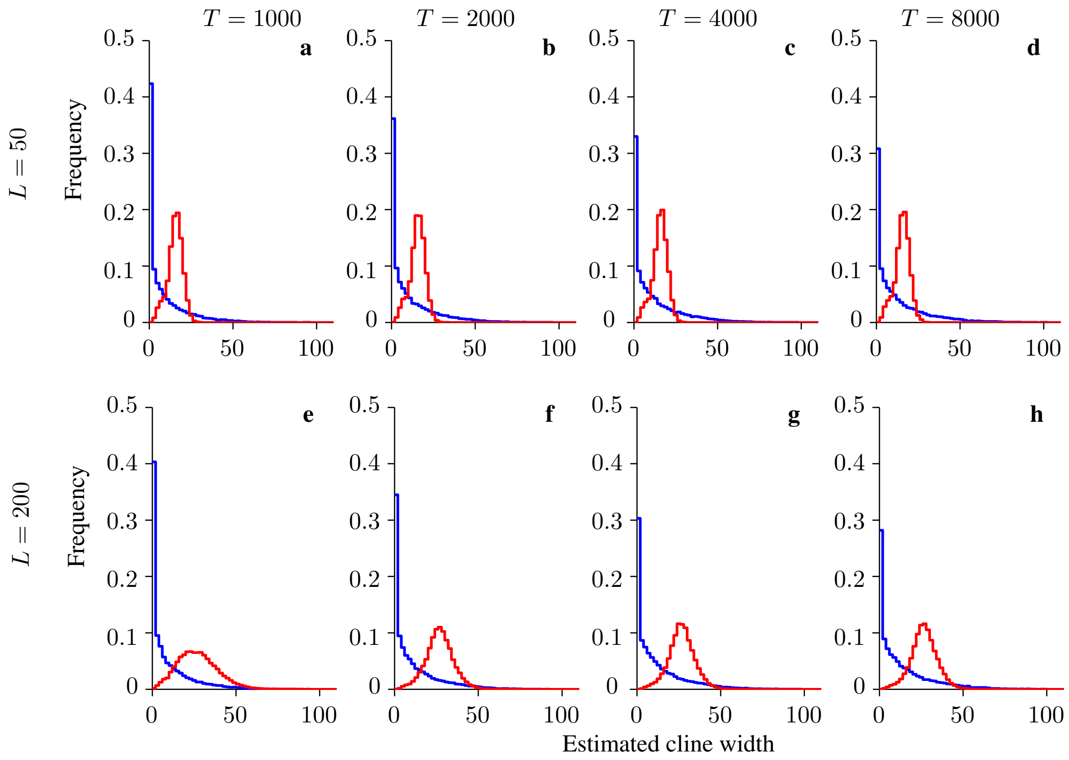

FIG. S1.5 Maximum-likelihood estimates of cline width for simulated allele-frequency data under the primary divergence model with $\sigma = 1.46$. Shown are results for neutral loci (blue), and selected loci (red). Number of loci under selection: $L = 50$ (**a**-**d**), $L = 200$ (**e**-**h**). Panels differ by the sampling time: $T = 1000$ (**a**, **e**), $T = 2000$ (**b**, **f**), $T = 4000$ (**c**, **g**), and $T = 8000$ (**d**, **h**). In all cases, the number of individuals in each patch was set to $N = 100$.

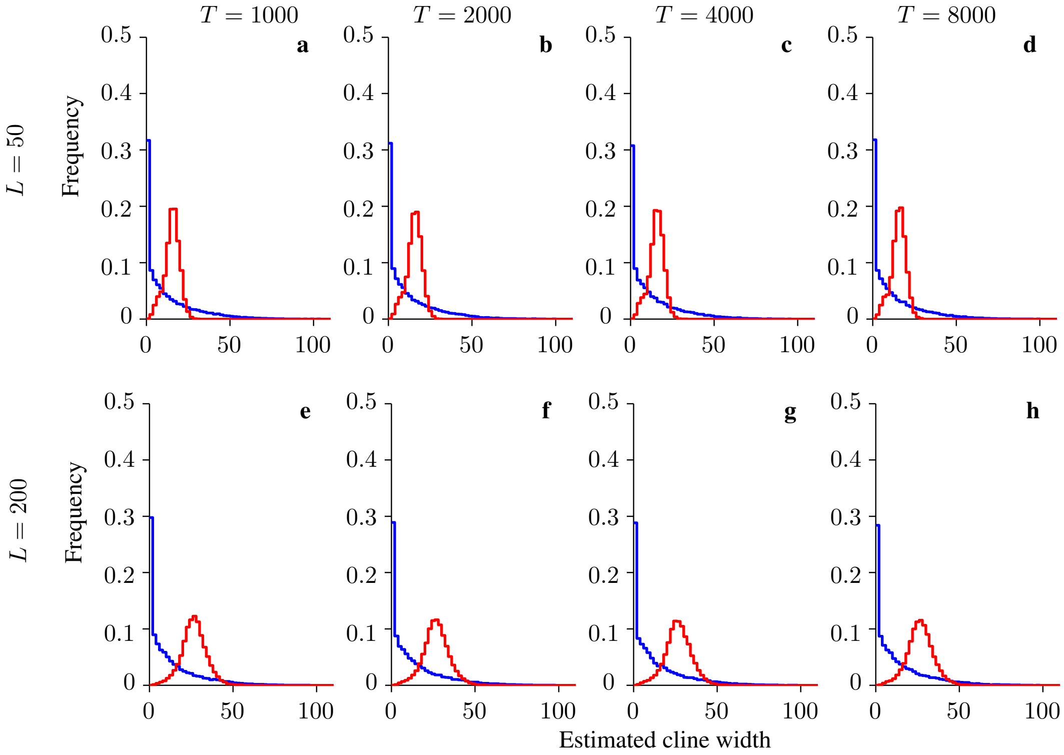

FIG. S1.6 Same as in Fig. S1.5, but for the secondary contact model.

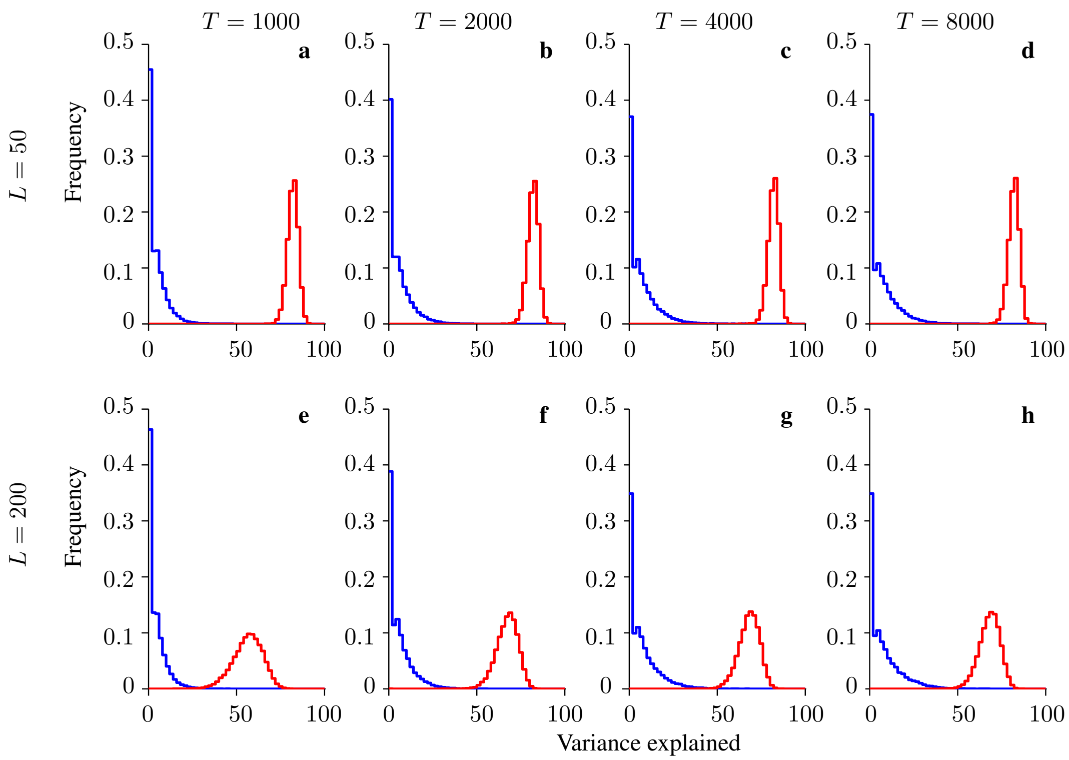

FIG. S1.7 Variance explained by the maximum-likelihood clinal fit for simulated allele-frequency data under the primary divergence model with $\sigma = 1.46$. Shown are results for neutral loci (blue), and selected loci (red). Number of loci under selection: $L = 50$ (**a**-**d**), $L = 200$ (**e**-**h**). Panels differ by the sampling time: $T = 1000$ (**a**, **e**), $T = 2000$ (**b**, **f**), $T = 4000$ (**c**, **g**), and $T = 8000$ (**d**, **h**). In all cases, the number of individuals in each patch was set to $N = 100$.

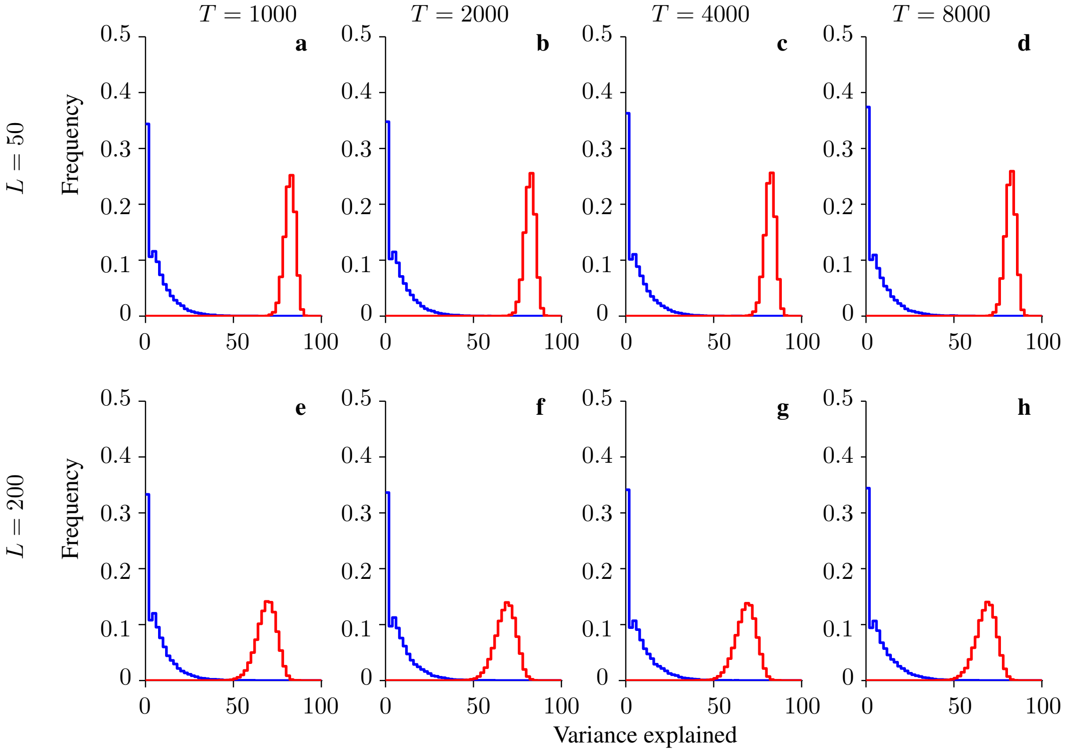

FIG. S1.8 Same as in Fig. S1.7, but for the secondary contact model.

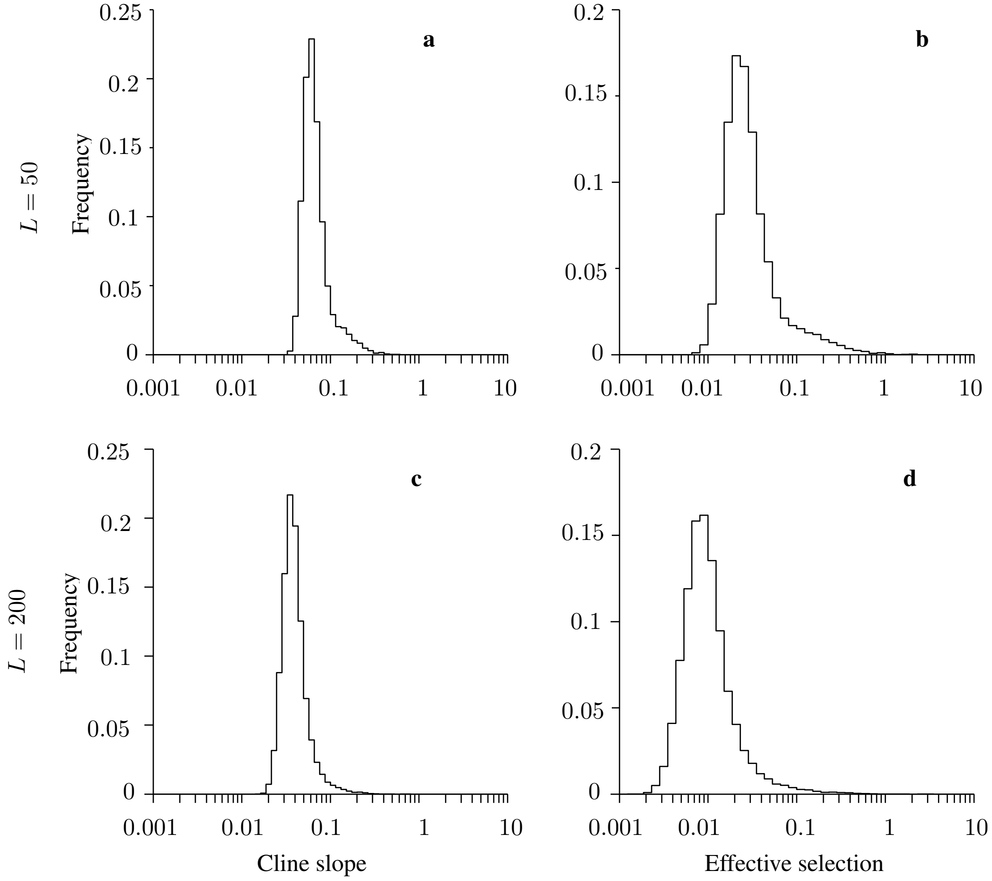

FIG. S1.9 Maximum-likelihood estimates of cline slopes (**a**, **c**), and effective selection coefficients (per locus) inferred from the estimated slopes (**b**, **d**) for loci under selection. Shown are only results for loci designated as clinal under the primary divergence model with $\sigma = 1.46$. Number of loci under selection: $L = 50$ (**a**-**b**), and $L = 200$ (**c**-**d**). Sampling time: $T = 4000$. In all cases, the number of individuals in each patch was set to $N = 100$.

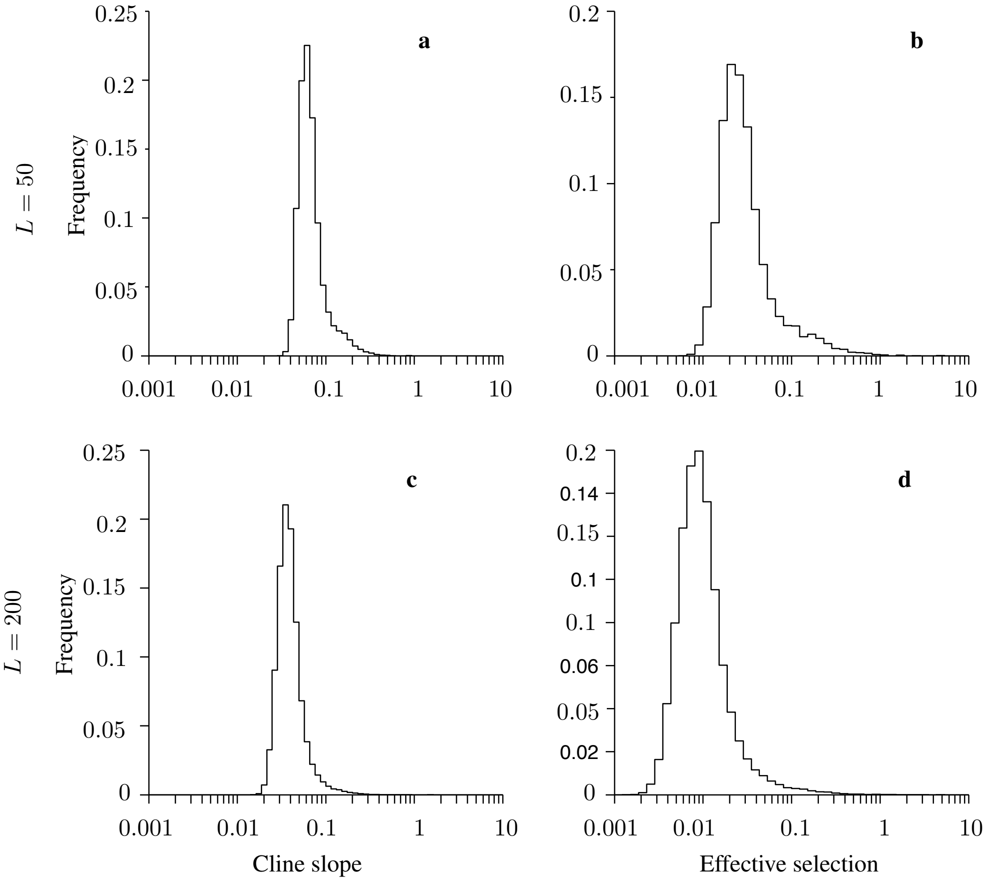

FIG. S1.10 Same as in Fig. S1.9, but for the secondary contact model.

References

(1) Akaike H (1987) Factor analysis and AIC. Psychometrika 52(3):317332.

(2) Barton NH (1979) Gene flow past a cline. Heredity 43(3):333.

(3) Butlin RK, et al. (2014) Parallel evolution of local adaptation and reproductive isolation in the face of gene flow. Evolution 68(4):935949.

(4) Panova M, Hollander J, Johannesson K (2006) Site-specific genetic divergence in parallel hybrid zones suggests nonallopatric evolution of reproductive barriers. Mol Ecol 15(13):40214031.

(5) Janson K (1983) Selection and migration in two distinct phenotypes of *Littorina saxatilis* in Sweden. Oecologia 59(1):5861.
